# Aspartate deficiency amplifies cGAS-STING signaling in antitumor immunity

**DOI:** 10.1172/JCI199716

**Published:** 2026-06-01

**Authors:** Yuheng Liao, Hanze Wang, Hengxin Liu, Xi Chen, Renqiang Sun, Xie Li, Zhen Yang, Chenying Liu, Wei Wu, Ziqian He, Yuzheng Zhao, Ying Mao, Dan Ye, Hui Yang

**Affiliations:** 1 Department of Neurosurgery, Huashan Hospital; and Molecular and Cell Biology Laboratory, Institutes of Biomedical Sciences, Shanghai Medical College, Fudan University, Shanghai, China.; 2 Institute for Translational Brain Research, Shanghai Key Laboratory of Brain Function Restoration and Neural Regeneration, MOE Frontiers Center for Brain Science, Shanghai Medical College, Fudan University, Shanghai, China.; 3 Optogenetics & Synthetic Biology Interdisciplinary Research Center, Shanghai Frontiers Science Center of Optogenetic Techniques for Cell Metabolism, State Key Laboratory of Bioreactor Engineering, School of Pharmacy, East China University of Science and Technology, Shanghai, China.; 4 Center for Medical Research and Innovation of Pudong Hospital and Intelligent Medicine Institute, Shanghai Medical College, Fudan University, Shanghai, China.; 5 Department of Colorectal and Anal Surgery, Xinhua Hospital, Shanghai Jiao Tong University School of Medicine, Shanghai, China.; 6 Key Laboratory of Multi-Cell Systems, Shanghai Institute of Biochemistry and Cell Biology, Center for Excellence in Molecular Cell Science, Chinese Academy of Sciences, University of Chinese Academy of Sciences, Shanghai, China.; 7 Department of Biostatistics, Mailman School of Public Health, Columbia University, New York, New York, USA.

**Keywords:** Metabolism, Oncology, Cellular immune response

## Abstract

Metabolic signals critically shape innate immune responses. Through pharmacological screening of metabolic pathways, we identified aspartate metabolism as a key regulator of cyclic GMP-AMP synthase (cGAS)–stimulator of interferon genes (STING) signaling. Genetically or aminooxyacetic acid–mediated (AOA-mediated) pharmacologically reducing aspartate levels markedly potentiated the cGAS-STING pathway, leading to stronger upregulation of type I interferons and interferon-stimulated genes. Mechanistically, disruption of de novo pyrimidine synthesis, a major downstream pathway of aspartate, induced mtDNA replication stress and increased mtDNA double-strand breaks, promoting mtDNA release into the cytosol. Cytosolic mtDNA synergized with cGAS-STING agonists to upregulate Z-DNA binding protein 1 (ZBP1), which recruits RIPK1/3 to sustain IRF3 phosphorylation, forming a positive feedback loop that amplifies innate immune signaling. In immunocompetent mouse models, AOA enhanced the antitumor efficacy of STING agonists, chemotherapy, or radiotherapy, whereas aspartate supplementation abrogated these effects. Consistently, aspartate levels negatively correlated with antitumor immunity in colorectal cancer patient samples. Together, our study identifies aspartate–pyrimidine metabolism as a critical metabolic checkpoint that licenses STING signaling by enabling mtDNA stress to cooperate with agonist stimulation, driving type I interferon–dependent ZBP1 induction and feed-forward amplification of STING signaling, thus offering a promising strategy to enhance antitumor immunity.

## Abstract 2

Aspartate deficiency enhances cGAS–STING signaling and antitumor immunity, revealing a metabolic mechanism that links cellular metabolism to innate immune activation.

## Graphical abstract

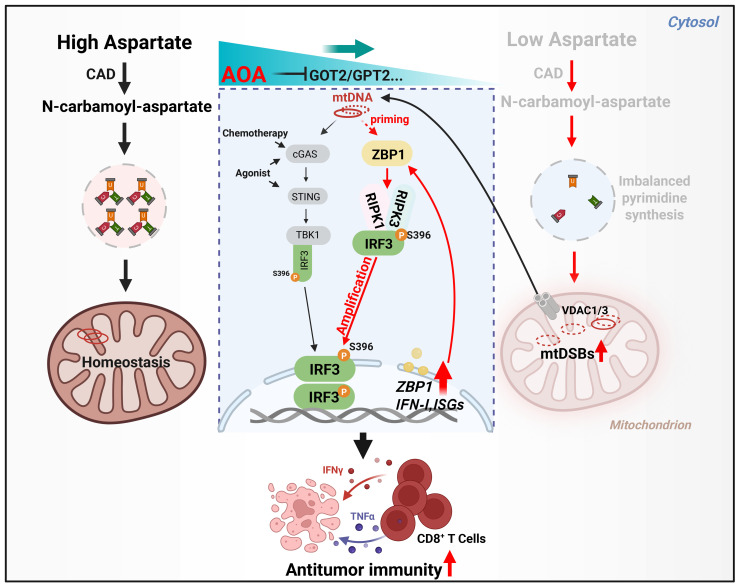

## Introduction

The cyclic GMP-AMP synthase (cGAS)–stimulator of interferon genes (STING) innate immune pathway is activated in response to abnormal cytosolic DNA exposure in many medical conditions (1). The cGAS enzyme specifically recognizes double-stranded DNA (dsDNA) in the cytosol, leading to the synthesis of cyclic GMP-AMP (cGAMP), a second messenger that activates STING. Upon activation, STING recruits TANK binding kinase 1 (TBK1) and interferon regulatory transcription factor 3 (IRF3), the latter of which undergoes phosphorylation, homodimerization, and nuclear translocation, culminating in the transcriptional upregulation of type I interferons (IFN-Is) and interferon-stimulated genes (ISGs) (1–3). The cGAS-STING pathway has essential roles in cancer immunity, activation of which in either tumor or immune cells can initiate T cell–dependent antitumor immunity (4).

Recent studies have shed light on the interplay between metabolic signals and the innate immune response, particularly in the context of the cGAS-STING pathway (5, 6). Aspartate, a nonessential amino acid, is crucial for intracellular protein manufacture, purine and pyrimidine biosynthesis, and cell proliferation (7–9). Recent studies have uncovered a novel role of aspartate beyond its classical function as a metabolic intermediate (10, 11). Intriguingly, the concentration of aspartate in the tumor interstitial fluid is several-fold higher than in plasma, suggesting an active role in shaping the tumor microenvironment (11, 12). These observations underscore the need to elucidate the mechanisms responsible for aspartate accumulation in tumors and to explore whether interfering with its intracellular biosynthesis could reshape antitumor immune responses via innate immune signaling.

In this study, we screened the impact of several metabolic pathways on the regulation of cGAS-STING signaling using small molecule metabolic inhibitors. This unbiased screen identified aminooxyacetic acid (AOA), an aminotransferase inhibitor, as the most dramatic amplifier of the cGAS-STING pathway. Mechanistically, AOA treatment leads to a profound reduction in intracellular aspartate levels by inhibiting a series of aminotransferases, such as glutamate oxaloacetate transaminase 2 (GOT2) and glutamate pyruvate transaminase 2 (GPT2), which are involved in key metabolic processes during aspartate synthesis. This in turn disrupts the carbamoyl-phosphate synthetase 2, aspartate transcarbamoylase, and dihydroorotase–mediated (CAD-mediated) de novo pyrimidine synthesis pathway, causing the release of mtDNA, a crucial event that amplifies the responsiveness of the cGAS-STING pathway to agonists, chemotherapy drugs, or radiotherapy via the Z-DNA binding protein 1–receptor interacting serine/threonine kinase 1/3 (ZBP1–RIPK1/3) axis. Moreover, a negative correlation between aspartate levels and T cell infiltration is identified in clinical samples of colorectal carcinoma. Together, our study unveils an essential connection between aspartate metabolism, the cGAS-STING pathway, and cancer immunity.

## Results

### The aminotransferase inhibitor AOA amplifies cGAS-STING signaling.

**Figure 1:**
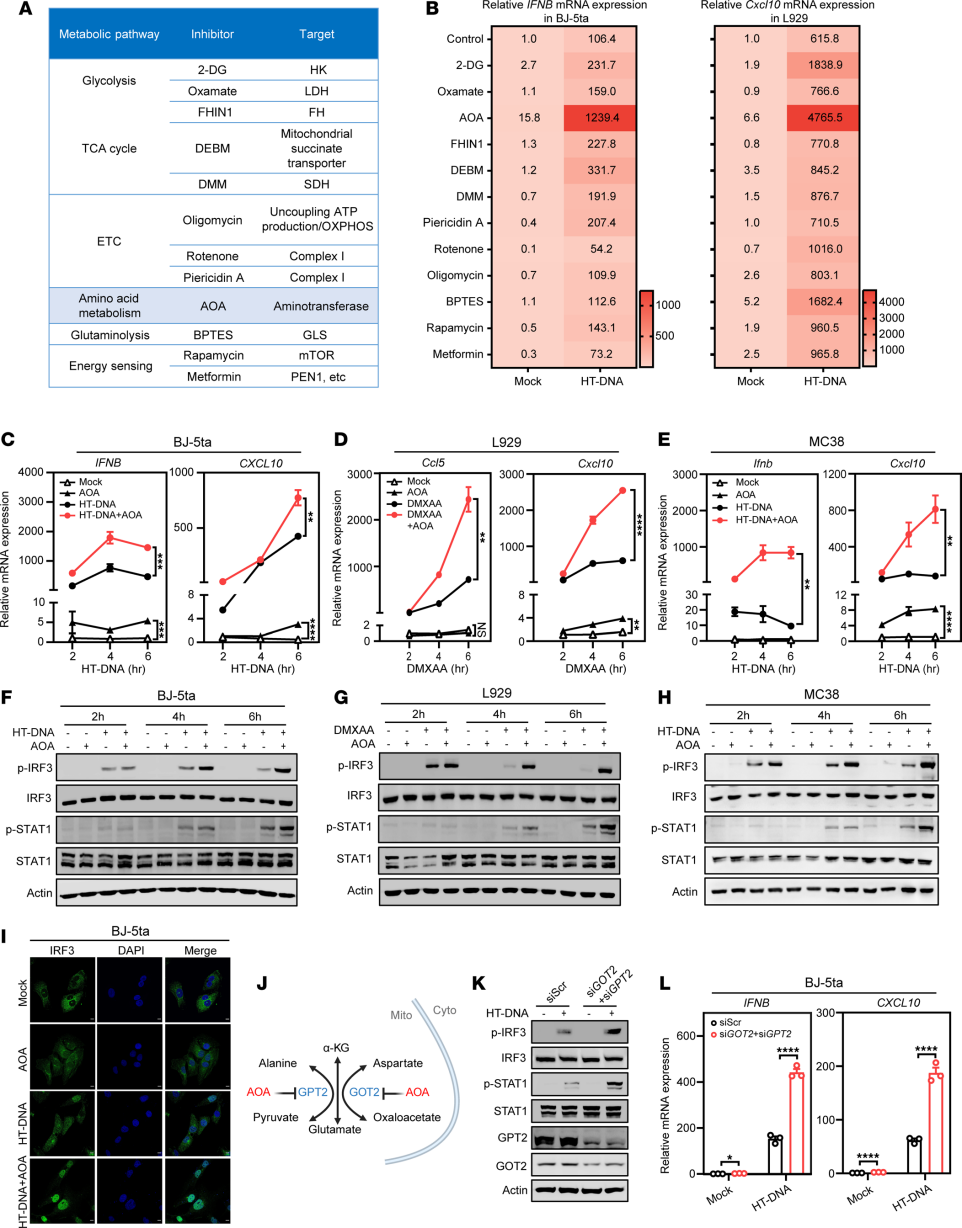
The aminotransferase inhibitor AOA amplifies cGAS-STING signaling. (**A**) Outline of metabolic inhibitors for screening. (**B**) Human hTERT-immortalized foreskin fibroblast (BJ-5ta, left) and mouse fibroblast (L929, right) cells were treated with inhibitors from **A** followed by 0.5 μg/mL HT-DNA transfection for 5 hours. Cells were harvested for qPCR analysis of human *IFNB* or mouse *Cxcl10*. (**C**) BJ-5ta cells were treated with 1 mM AOA for 1 hour followed by HT-DNA (0.5 μg/mL) transfection for the indicated time. Cells were harvested for qPCR analysis of IFN response gene expression. (**D** and **E**) L929 (**D**) and MC38 (**E**) were treated with 0.5 mM AOA for 1 hour followed by DMXAA (50 μM) or HT-DNA (0.5 μg/mL) stimulation for the indicated time. Cells were harvested for qPCR analysis of IFN response gene expression. (**F**–**H**) Western blot detected phosphorylated (p-) p-IRF3 and p-STAT1 levels in BJ-5ta, L929, and MC38 cells treated as in **C**–**E**. (**I**) Representative immunofluorescence images of IRF3 in BJ-5ta cells treated with AOA 1 hour followed by HT-DNA (0.5 μg/mL) transfection or not. Scale bars, 5 μm. (**J**) Schematic of the main targets of AOA. (**K**) Western blot detected p-IRF3, p-STAT1, GOT2, and GPT2 levels in BJ-5ta cells treated as indicated. (**L**) The relative *IFNB* and *CXCL10* mRNA expression in the control BJ-5ta cells versus GOT2 and GPT2 siRNA-silenced BJ-5ta cells after indicated treatment. Data are represented as means ± SEM. Representative data are shown from 2 or 3 independent experiments. Statistical analysis was performed by unpaired *t* test (**C**–**E**, **I**, and **L**). *P < 0.05; **P < 0.01; ***P < 0.001; ****P < 0.0001.

To explore the impact of metabolic changes on the cGAS-STING pathway, we screened the effects of small molecule inhibitors targeting various metabolic enzymes and pathways, encompassing glycolysis, the tricarboxylic acid (TCA) cycle, the electron transport chain, glutaminolysis, and energy sensing (Figure 1A). Three cell lines were used, including human hTERT-immortalized foreskin fibroblast cells (BJ-5ta), murine fibroblast cells (L929), and colon cancer cells (MC38). Cells were pretreated with each of the metabolic inhibitors and then transfected with herring testis DNA (HT-DNA), a commonly used dsDNA ligand for cGAS, following which the mRNA levels of *Ifnb* and *Cxcl10* were examined. In addition to the previously reported 2-deoxyglucose (13), we identified that aminotransferase inhibitor AOA exhibited the most profound effect on the upregulation of *Ifnb* and *Cxcl10* in distinct cell lines, particularly after HT-DNA transfection (Figure 1B and Supplemental Figure 1A; supplemental material available online with this article; https://doi.org/10.1172/JCI199716DS1). Although AOA alone was able to induce limited upregulation of *IFNB*/*ISG*s, such priming drastically increased responses to additional stimuli, such as the natural STING agonist 2′,3′-cGAMP or mouse STING-specific agonist DMXAA in murine macrophages (RAW264.7), murine melanoma cells (B16-F10), and human fibrosarcoma cells (HT1080) (Supplemental Figure 1, A–D). Such boosting effects exhibited a time-dependent manner upon stimulation with HT-DNA or DMXAA (Figure 1, C–E). Consistently, the transcriptional induction of *IFNB* was accompanied by a corresponding increase in its protein production and secretion (Supplemental Figure 1, E–G). Thus, the aminobutyrate transaminase inhibitor AOA robustly enhances the activation of the cGAS-STING pathway across multiple cell lines.

In light of IRF3 phosphorylation and IFN-I signaling (STAT1 phosphorylation) as a key downstream event in the cGAS-STING signaling cascade governing *ISG* production (2, 3, 14), we evaluated the impact of AOA on IRF3 and STAT1 phosphorylation. AOA dramatically prolonged the phosphorylation of IRF3 and STAT1 following agonist treatment in BJ-5ta, L929, and MC38 cells (Figure 1, F–H), a pattern also observed in RAW264.7, HT-1080, and B16-F10 (Supplemental Figure 1, H–J). However, no obvious IRF3 and STAT1 activation/phosphorylation was detected after AOA treatment alone (Figure 1, F–H, and Supplemental Figure 1, H–J). It is likely that the levels of phosphorylated IRF3 and STAT1 might be below detection, given the rather weak ISG upregulation by AOA alone (Figure 1, B–E). IRF3 phosphorylation by TBK1 is a major mechanism driving the dimerization and nuclear translocation of IRF3 (15, 16). Consistently, in unstimulated cells, we observed that IRF3 was primarily located in the cytoplasm (Figure 1I). AOA treatment alone was insufficient to trigger significant nuclear accumulation of IRF3. In contrast, compared with HT-DNA treatment alone, AOA cotreatment prolonged IRF3 nuclear localization, maintaining strong nuclear translocation even at 6 hours (Figure 1I). Furthermore, we observed that loss of cGAS, STING, or IRF3 not only eliminated ISG expression induced by AOA treatment alone but also completely abolished the enhancing effect of AOA in response to HT-DNA in L929 cells (Supplemental Figure 1, K and L), confirming the necessity of cGAS-STING signaling in the upregulation of *ISG* expression by AOA. In contrast, deficiency of RIG-I or MDA5 did not impair the AOA-mediated enhancement of ISG expression (Supplemental Figure 1, M–O), indicating a predominant role for DNA sensing rather than RNA sensing.

AOA is a pan-transaminase inhibitor against multiple metabolic enzymes, such as GOT1 and GOT2 (17–19). To identify the primary AOA target(s) responsible for the observed effect on amplifying cGAS-STING activation, we generated BJ-5ta cells depleted of *GOT1* or *GOT2*. Unexpectedly, individual deletion of either enzyme did not affect the transcriptional response of the DNA-sensing pathway (Supplemental Figure 2, A and B). Due to potential compensatory effects between metabolic pathways, it might be difficult to mimic the rapid remodeling of cell metabolism by AOA through single gene deletion (18, 20). Indeed, the mRNA expression of *GPT2*, another important transaminase targeted by AOA (18, 19), was upregulated in BJ cells with *GOT2* knockdown (Supplemental Figure 2C). As expected, when we constructed a cell line with *GPT2* knockdown alone, we were still unable to replicate the enhancement of ISG expression induced by AOA (Supplemental Figure 2, D and E). We hypothesized that GPT2 might partially compensate for the function of GOT2 in cells when *GOT2* was knocked down. This prompted us to generate cells with codepletion of GOT2 and GPT2 in BJ-5ta and MC38 cells (Figure 1, J–L, and Supplemental Figure 2, F and G). Strikingly, these double-knockdown cells exhibited slightly increased ISG expression in resting conditions but showed profound amplification of DNA-stimulated ISG expression, accompanied by increased phosphorylation of IRF3 and STAT1 (Figure 1, J–L, and Supplemental Figure 2, F and G). Consistently, AOA treatment failed to further augment ISG expression or downstream effector activation in *GOT2*/*GPT2* double-knockdown cells (Supplemental Figure 2, H and I), suggesting that inhibition of GOT2/GPT2 is a principal mechanism by which AOA enhances cGAS-STING–mediated innate immune signaling. Thus, AOA treatment or codepletion of GOT2 and GPT2 triggers a low level of ISG expression on their own, but each of them boosts the cGAS-STING pathway in response to agonists.

### Cellular aspartate deficiency augments cGAS-STING–mediated IFN response.

**Figure 2:**
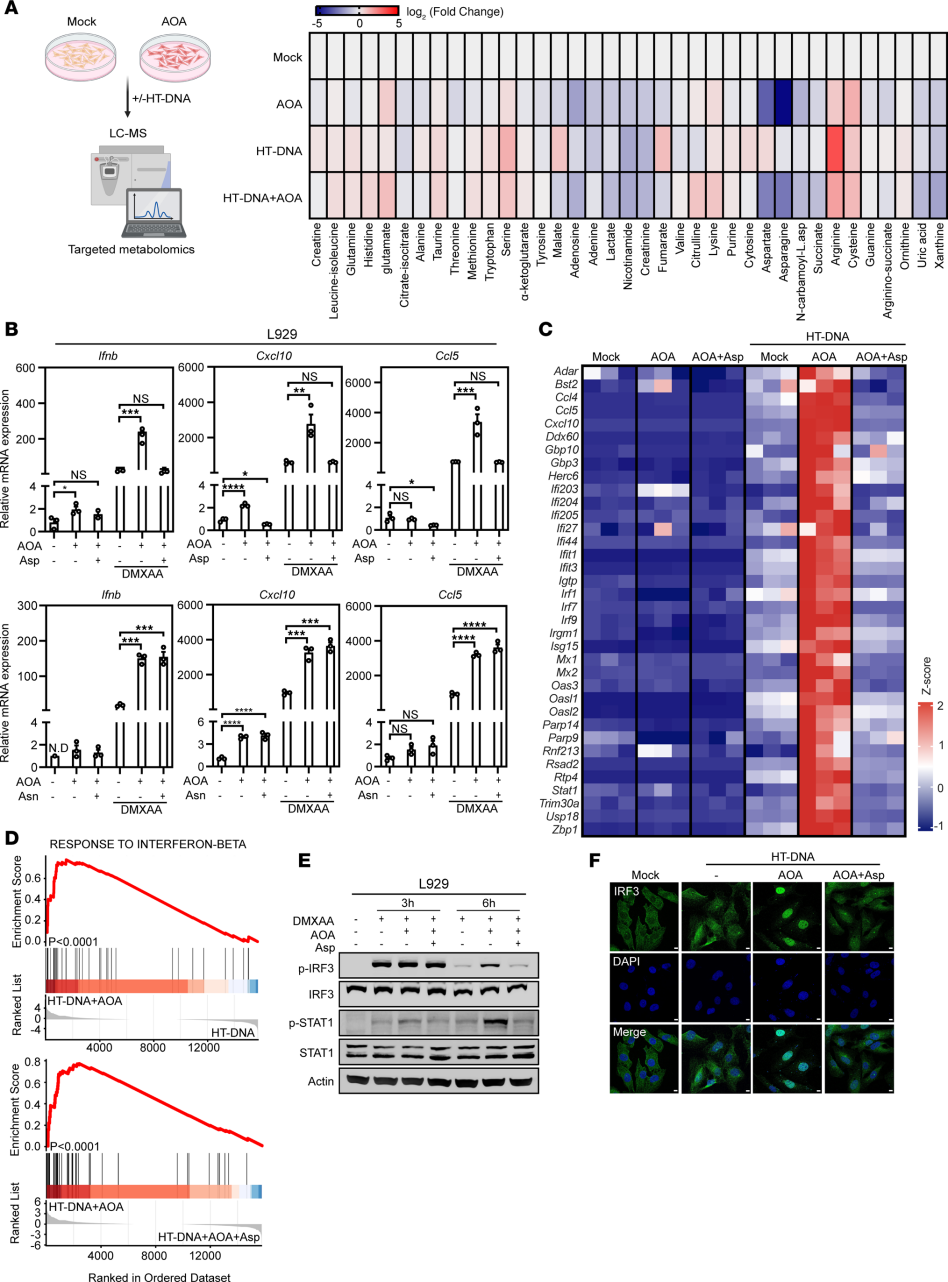
AOA augments cGAS-STING–mediated IFN response by inducing aspartate deficiency. (**A**) Heatmap of metabolite changes in mock- and AOA-treated L929 cells followed with HT-DNA transfection or without HT-DNA transfection. The fold-change of metabolites abundances was normalized to the mock group. Each square represents the mean of 3 replicates (*n* = 3 independent cultures). (**B**) L929 cells were treated with 0.5 mM AOA for 1 hour followed by DMXAA (50 μM) stimulation for 6 hours in the absence or presence of 20 mM aspartate or asparagine, and then cells were harvested for qPCR analysis of IFN response gene expression. (**C**) Heatmap from RNA-seq revealing the expression of ISGs in L929 cells with indicated treatment compared with mock group [*z*-score–normalized log_2_(fold per million reads) values, *n* = 3 independent cultures]. (**D**) GSEA of interferon-beta response gene. (**E**) Western blot detected p-IRF3 and p-STAT1 levels in L929 cells treated as indicated. (**F**) Representative immunofluorescence images of IRF3 in BJ-5ta cells treated with AOA 1 hour followed by HT-DNA (0.5 μg/mL) transfection in the absence or presence of 20 mM aspartate. Scale bars, 5 μm. Data are represented as means ± SEM. Representative data are shown from 2 or 3 independent experiments. Statistical analysis was performed by 1-way ANOVA followed by Tukey’s test (**B**). *P < 0.05; **P < 0.01; ***P < 0.001; ****P < 0.0001.

Next, we explored the specific cellular metabolites AOA influences for amplifying the cGAS-STING pathway. A targeted metabolomics approach utilizing liquid chromatography–mass spectrometry (LC-MS) characterized the metabolome of L929 cells pretreated with AOA and transfected with HT-DNA or not (Figure 2A). Importantly, aspartate and asparagine were identified as 2 of the most significantly downregulated metabolites, both reduced by approximately 80%, respectively, in L929 cells 6 hours after AOA treatment (Figure 2A and Supplemental Figure 3A). Consistently, a sharp decrease in aspartate was also found in *GOT2* and *GPT2* double-knockdown cells (Supplemental Figure 3B). To assess the functional significance of aspartate and asparagine in the cGAS-STING pathway, these 2 metabolites were individually added along with AOA to the culture medium, followed by DMXAA stimulation for 6 hours. Interestingly, aspartate, but not asparagine, fully reversed the amplification effect of AOA on STING-mediated immune response (Figure 2B and Supplemental Figure 3, C–G).

RNA-seq analysis further corroborated that although aspartate deficiency caused by AOA alone only induced weak ISG expression, it acts as a fuel to sensitize the cGAS-STING signaling in response to agonists in L929 cells (Figure 2C). Gene set enrichment analysis (GSEA) revealed that genes involved in IFN-I response were substantially enriched in aspartate-deficient cells following HT-DNA stimulation compared with aspartate-sufficient groups (Figure 2D). Functional pathway enrichment analysis highlighted the alteration of cytosolic DNA–sensing pathway as one of the top enriched pathways in aspartate-deficient cells compared with those with aspartate supplementation (Supplemental Figure 3H). Consistent with these findings, aspartate could also reverse the effect of AOA on prolonging IRF3 and STAT1 phosphorylation and IRF3 nuclear translocation in response to agonists in L929 cells (Figure 2, E and F). Similar results were also observed in other human or murine cell lines, such as BJ-5ta, HT-1080, RAW264.7, B16-F10, and MC38 cells (Supplemental Figure 3, I–M). Collectively, these results indicate that AOA enhances the immune response by substantially reducing cellular aspartate levels, suggesting that aspartate deficiency can augment cGAS-STING–mediated IFN-I responses.

### AOA amplifies IFN response via disrupting aspartate-dependent pyrimidine synthesis.

**Figure 3:**
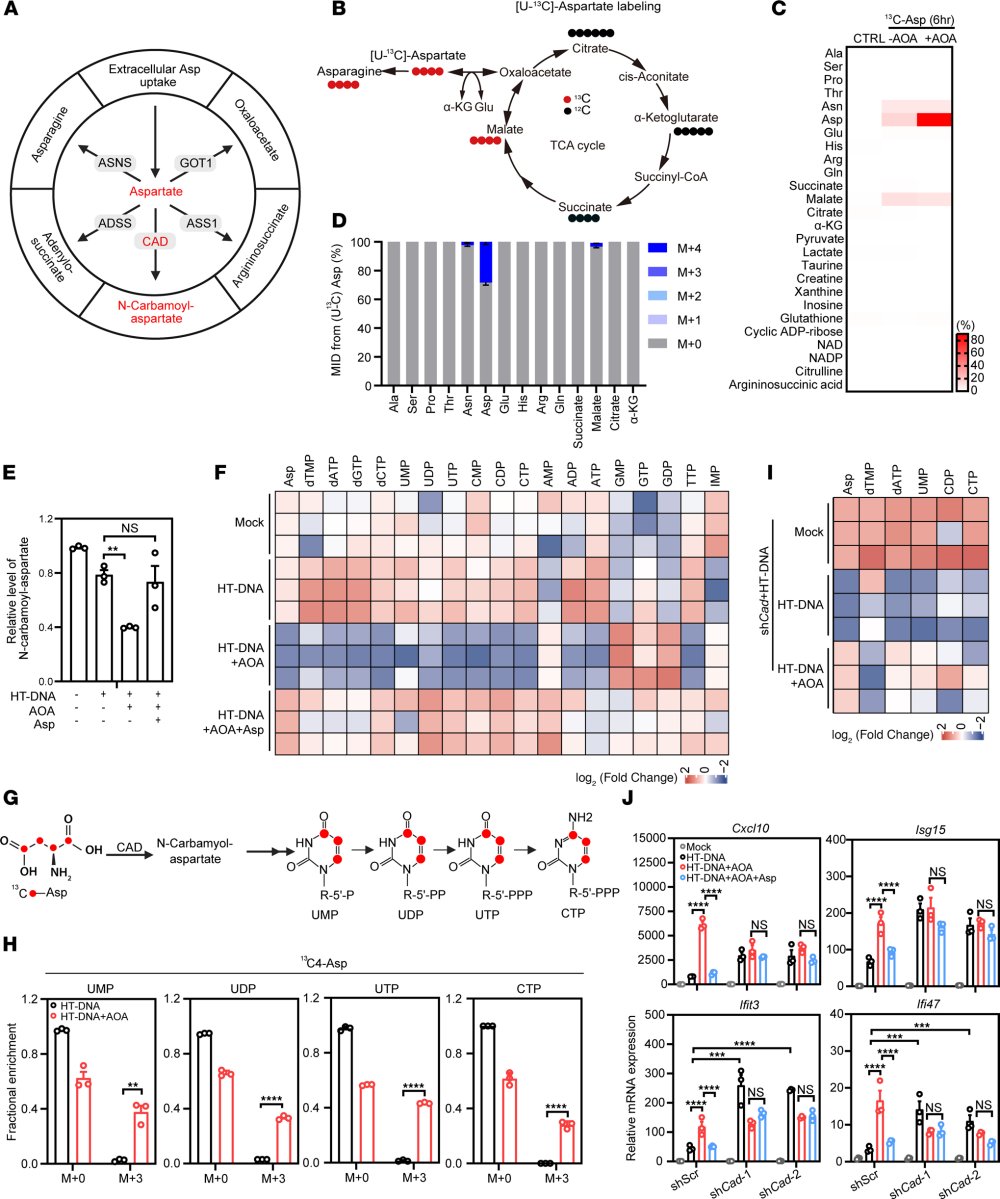
AOA amplifies IFN response via disrupting aspartate-dependent pyrimidine synthesis. (**A**) Schematic illustration of cellular aspartate uptake and catabolism. (**B**) Schematic showing ^13^C_4_-aspartate flux in TCA cycle and asparagine synthesis. (**C**) L929 cells were treated with or without AOA in medium containing 20 mM ^13^C_4_-aspartate. The abundances of metabolites labeled with the stable isotope ^13^C in cells were analyzed by liquid chromatography–tandem mass spectrometry (LC–MS/MS). The heatmap shows the percentage of isotope-labeled metabolite relative to the total content of each metabolite. (**D**) Mass isotopologue distribution (MID) of cellular metabolites in TCA and amino acids after AOA+^13^C_4_-aspartate treatment. (**E**) Relative abundance of N-carbamoyl-aspartate (*n* = 3 independent cultures). L929 cells were treated with AOA in the absence or presence of 20 mM aspartate followed by HT-DNA transfection and then collected for LC–MS/MS analysis. (**F**) Heatmap of log_2_ (fold-change) indicates nucleotide levels of L929 cells treated as **E** performed by LC–MS/MS. Heatmap of log_2_ (fold-change) in the indicated nucleotide levels in treatment groups compared with mock. Each square represents an individual replicate (*n* = 3 independent cultures). (**G**) Schematic diagram shows ^13^C_4_-aspartate as a tracer to pyrimidines. (**H**) Pyrimidines from ^13^C_4_-aspartate in L929 cells treated with or without AOA followed by HT-DNA transfection (*n* = 3 independent cultures). (**I**) Heatmap of log_2_ (fold-change) of the indicated nucleotide levels in AOA- and AOA+Asp–treated groups compared with the mock group after HT-DNA stimulation in *Cad*-knockdown L929 cells (*n* = 3 independent cultures). (**J**) ISGs expression in Scr and *Cad*-knockdown L929 cells treated as **E**. Data are represented as means ± SEM. Representative data are shown from 2 or 3 independent experiments. Statistical analysis was performed by unpaired *t* test (**H**) and 1-way ANOVA (**E**) or 2-way ANOVA followed by Tukey’s test (**J**). **P < 0.01; ***P < 0.001; ****P < 0.0001.

Intracellular aspartate is known to play a significant role in metabolic switching (7, 9) (Figure 3A). To investigate the regulatory roles of aspartate metabolism in the cGAS-STING pathway, we employed stable isotope–tracing technology with [U-13C]-aspartate to determine if aspartate is converted into other downstream metabolites to achieve its reversal effect (Figure 3B). Compared with the control group, cells treated with AOA exhibited increased uptake of [U-13C]-aspartate (Figure 3C). However, aspartate did not undergo further metabolism into adjacent amino acids or intermediates of the TCA cycle and urea cycle (Figure 3, C and D, and Supplemental Figure 4A). Moreover, supplementing metabolites in the urea cycle and TCA cycle, such as malate, α-ketoglutarate, succinate, fumarate, arginine, citrulline, and argininosuccinate, failed to reverse AOA-mediated enhancement of ISG expression (Supplemental Figure 4, B–J). These findings indicate that the augmentation of the cGAS-STING pathway mediated by aspartate deficiency is not due to its impact on metabolites in the TCA cycle or urea cycle. Previous studies reported that AOA can also change cellular NAD^+^/NADH ratio and increase ROS production (Supplemental Figure 4K) (21, 22). We confirmed that AOA indeed reduced the intracellular NAD to NADH ratios, but supplementation of aspartate did not reverse such change (Supplemental Figure 4L). Supplementation of N-acetyl-cysteine, which suppresses ROS production, failed to change the expression of ISGs in L929 cells (Supplemental Figure 4M). Thus, changes in redox homeostasis do not appear to contribute to the effect of AOA on the cGAS-STING pathway.

Besides its role in metabolic pathways like amino acid and protein synthesis, aspartate serves as a key precursor of purines and pyrimidines (23–25). Accordingly, metabolomic analysis revealed that N-carbamoyl-aspartate, an intermediate in de novo pyrimidine synthesis pathway, was reduced by AOA treatment, which was reversed by aspartate supplementation (Figure 3E). Furthermore, nucleotides containing pyrimidines were remarkably downregulated in AOA-treated cells, and this effect was completely rescued through aspartate supplementation (Figure 3F). It is known that aspartate contributes 3 carbon atoms and 1 nitrogen atom of the rings in uracil, cytosine, and thymine (26) (Figure 3G). [U-13C]-aspartate tracing experiment demonstrated that exogenous aspartate carbon could serve as a source for pyrimidine synthesis in L929 cells during cellular aspartate deficiency (Figure 3H). Collectively, our data suggest that in the examined cells experiencing aspartate deficiency, supplemented aspartate was primarily used to facilitate the synthesis of pyrimidine nucleotides. We next examined whether pyrimidine supplementation modulates the AOA-enhanced activation of the cGAS-STING pathway. Supplementing AOA-treated L929 and MC38 cells with a combination of thymidine, cytidine, and uridine nucleosides was sufficient to reverse the enhancement of *ISG* expression (Supplemental Figure 5, A and B), confirming that the reversal effect of aspartate on *ISG* expression is through its participation in pyrimidine nucleotide synthesis. Furthermore, depletion of carbamoyl-phosphate synthetase 2, aspartate transcarbamoylase, and dihydroorotase (CAD; Supplemental Figure 5C), a key enzyme in pyrimidine synthesis from aspartate (27), abolished the capability of aspartate supplementation to rescue cellular pyrimidine nucleotide levels (Figure 3I) and to reverse the potentiating effects of AOA on the cGAS-STING pathway (Figure 3J). These findings indicate the crucial role of de novo pyrimidine synthesis in aspartate-mediated regulation of ISG expression.

### Aspartate deficiency induces mtDNA double-strand breaks, leading to its release into the cytoplasm.

**Figure 4:**
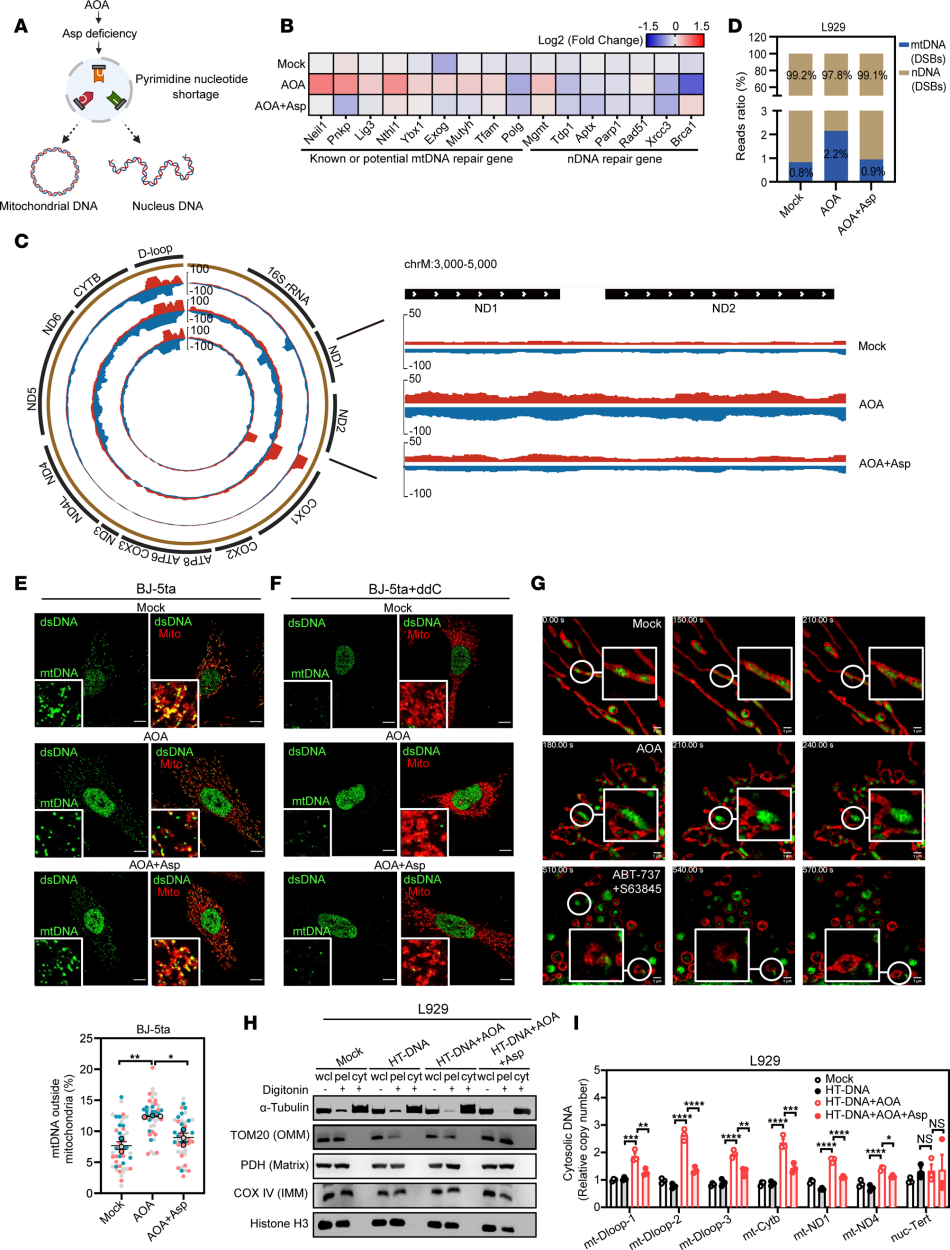
Aspartate deficiency induces mtDNA DSBs, leading to its release into the cytoplasm. (**A**) Schematic showing that aspartate-mediated pyrimidine nucleotide synthesis is essential for intracellular DNA homeostasis. (**B**) Heatmap from RNA-seq revealing the expression of DNA repair enzymes in L929 cells with indicated treatment. The fold-change of expression was normalized to the mock group (*n* = 3 independent cultures). (**C**) Genome browser screenshots of END-seq on mitochondria from L929 cells with indicated treatment. The expanded view of the indicated region shows more detailed genomic features. (**D**) The proportion of END-seq reads in mitochondrial and nuclear chromosomal regions. (**E**) Representative immunofluorescence images of mitochondrial (TOM20, red) or dsDNA (green) in BJ-5ta cells treated with AOA in the absence or presence of 20 mM aspartate for 6 hours. Scale bars, 10 μm. Lower panel: quantification of cytosolic dsDNA in BJ-5ta cells with indicated treatment (*n* = 39–51 fields per group from 3 biological replicates). (**F**) Representative immunofluorescence images of BJ-5ta cells treated with ddC (100 μM) for 72 hours, followed by 6 hours of AOA treatment in the absence or presence of 20 mM aspartate. (**G**) 2D–structured illumination microscopy imaging of mtDNA externalization under the indicated conditions. (**H**) Western blot detected α-tubulin (cytosol), TOM20 (OMM), PDH (Matrix), COX IV (IMM), and histone H3 (nuclei) to validate protocol from Supplemental Figure 7A in L929 cells treated as indicated. wcl, whole cell lysate; pel, pellet; cyt, cytosolic fraction. (**I**) L929 cells were treated with AOA in the absence or presence of 20 mM aspartate followed by HT-DNA transfection, and then cells were harvested for qPCR analysis of mtDNA or nDNA levels in cytosolic fractions. Data are represented as means ± SEM. Representative data are shown from 2 or 3 independent experiments. Statistical analysis was performed by 1-way ANOVA followed by Tukey’s test (**E** and **I**). *P < 0.05; **P < 0.01; ***P < 0.001; ****P < 0.0001. DSBs, double-strand breaks.

The pyrimidine nucleotide pool serves as an essential precursor for intracellular DNA synthesis (9). Since DNA is primarily stored in the nucleus and mitochondria, we sought to determine whether aspartate deficiency could induce DNA damage in these compartments (Figure 4A). Upon AOA treatment, we observed a moderate upregulation of genes known or potentially involved in mtDNA damage repair (28), which can be reversed by aspartate supplementation. In contrast, the expression of genes associated with nuclear DNA (nDNA) damage repair showed no significant change (Figure 4B). Consistently, no significant elevation of the nuclear DNA damage marker γH2AX was detected following AOA treatment (Supplemental Figure 6, A and B), suggesting that nDNA integrity remains largely preserved under aspartate-deficient conditions. To further explore the location of DNA damage, we performed END-seq to map the distribution of DNA DSBs. Remarkably, AOA treatment led to a marked increase in DSB frequency in mtDNA but not in nDNA (Figure 4, C and D). This increase was reversed upon aspartate supplementation (Figure 4, C and D), indicating that aspartate deficiency specifically compromises mtDNA integrity. Consistent with this notion, AOA treatment caused a more pronounced reduction of aspartate levels in mitochondria, whereas nuclear aspartate levels were relatively preserved (Supplemental Figure 6, C–F), suggesting that under conditions of limited nucleotide availability, cells prioritize the maintenance of nuclear genome integrity, rendering mtDNA more susceptible to replication stress and fragmentation. These findings raise the intriguing question of whether the damaged mtDNA is subsequently released into the cytosol. We performed antibody-based immunofluorescence staining of dsDNA. AOA-treated BJ cells exhibited a markedly increased amount of cytosolic dsDNA outside of the mitochondria, a phenomenon reversed by aspartate supplementation (Figure 4E). Furthermore, depletion of cellular mtDNA by 2′,3′-dideoxycytidine (ddC) in treated BJ-5ta cells substantially reduced the release of cytosolic dsDNA outside of the mitochondria (Figure 4F). We further found that the release of mtDNA induced by aspartate deficiency is mechanistically distinct from the classical apoptosis-mediated mtDNA release. Notably, it occurs without overt disruption of mitochondrial ultrastructure (Figure 4G and Supplemental Videos 1–3). Additionally, Bcl-2-associated X protein/Bcl-2 homologous antagonist/killer (BAK/BAX) macropores, known to facilitate mitochondrial herniation and the efflux of mtDNA during apoptosis (29–31), were not involved in aspartate deficiency–induced mtDNA release, since *ISG* expression was still enhanced by AOA treatment when BAX/BAK were codepleted (Supplemental Figure 6, G and H). Consistently, we measured cell death by FVS780 and annexin V staining and found that low-dose AOA treatment did not induce substantial apoptosis (Supplemental Figure 6, I and J). These findings suggest that the mtDNA release induced by AOA-mediated metabolic stress is limited and reversible, leading to a comparatively weak ISG response relative to apoptosis-induced mtDNA release. To verify this observation, L929 and MC38 cells were treated with AOA in the presence or absence of aspartate, followed with HT-DNA transfection or without, and subsequently fractionated to examine cytosolic mtDNA content (Supplemental Figure 7A). The successful isolation of a pure cytosolic fraction was confirmed by immunoblot analysis (Figure 4H and Supplemental Figure 7B). qPCR analysis revealed that AOA-treated cells displayed a significant enrichment of mtDNA in the cytosolic fraction, which contains mitochondrial genes (e.g., *Dloop1*, *Dloop2*, *Dloop3*, *Nd1*, *Nd4*, *CytB*) (Figure 4I and Supplemental Figure 7, C–E). More importantly, this increase could be reversed by aspartate supplementation, reaffirming that cellular aspartate deficiency can trigger the release of mtDNA fragments (Figure 4I and Supplemental Figure 7, C–E). Consistently, codepletion of *GOT2* and *GPT2* in L929 and MC38 cells led to increased mtDNA release to the cytosol (Supplemental Figure 7, F and G). To further examine whether the enhanced innate immune response observed in AOA-pretreated cells depends on mtDNA release, we depleted mtDNA from wild-type and AOA-treated cells using ddC. Treatment with ddC efficiently depleted mtDNA from cells over time and resulted in strong suppression of ISG expression in AOA-treated cells upon stimulation (Supplemental Figure 7, H and I), suggesting a synergistic response of mtDNA release and agonists of cGAS-STING pathway. Notably, no detectable leakage of mitochondrial RNA (mtRNA) was observed under these conditions (Supplemental Figure 7J), indicating that the potentiation of innate immune signaling by AOA is specifically associated with mtDNA, rather than mtRNA release.

Mitochondrial voltage-dependent anion channel (VDAC) oligomers are known to form mitochondrial pores for the release of mtDNA fragments (32). Strikingly, in the presence of VBIT-4, a VDAC inhibitor, or upon *VDAC1/3* knockdown, AOA treatment no longer induced mtDNA release or enhanced agonist-induced *ISG* expression or IRF3 and STAT1 phosphorylation in cells (Supplemental Figure 8, A–G). Collectively, our data strongly suggest that a deficiency in aspartate or pyrimidine nucleotides leads to an increased incidence of mtDNA DSBs rather than nDNA DSBs. Given that mtDNA lacks the robust DSB repair mechanisms present in the nucleus, this deficiency results in the release of fragmented mtDNA into the cytoplasm via mitochondrial VDAC oligomerization, thereby activating the innate immune response.

### AOA prolongs IRF3 phosphorylation via the ZBP1–RIPK1/3 axis.

**Figure 5:**
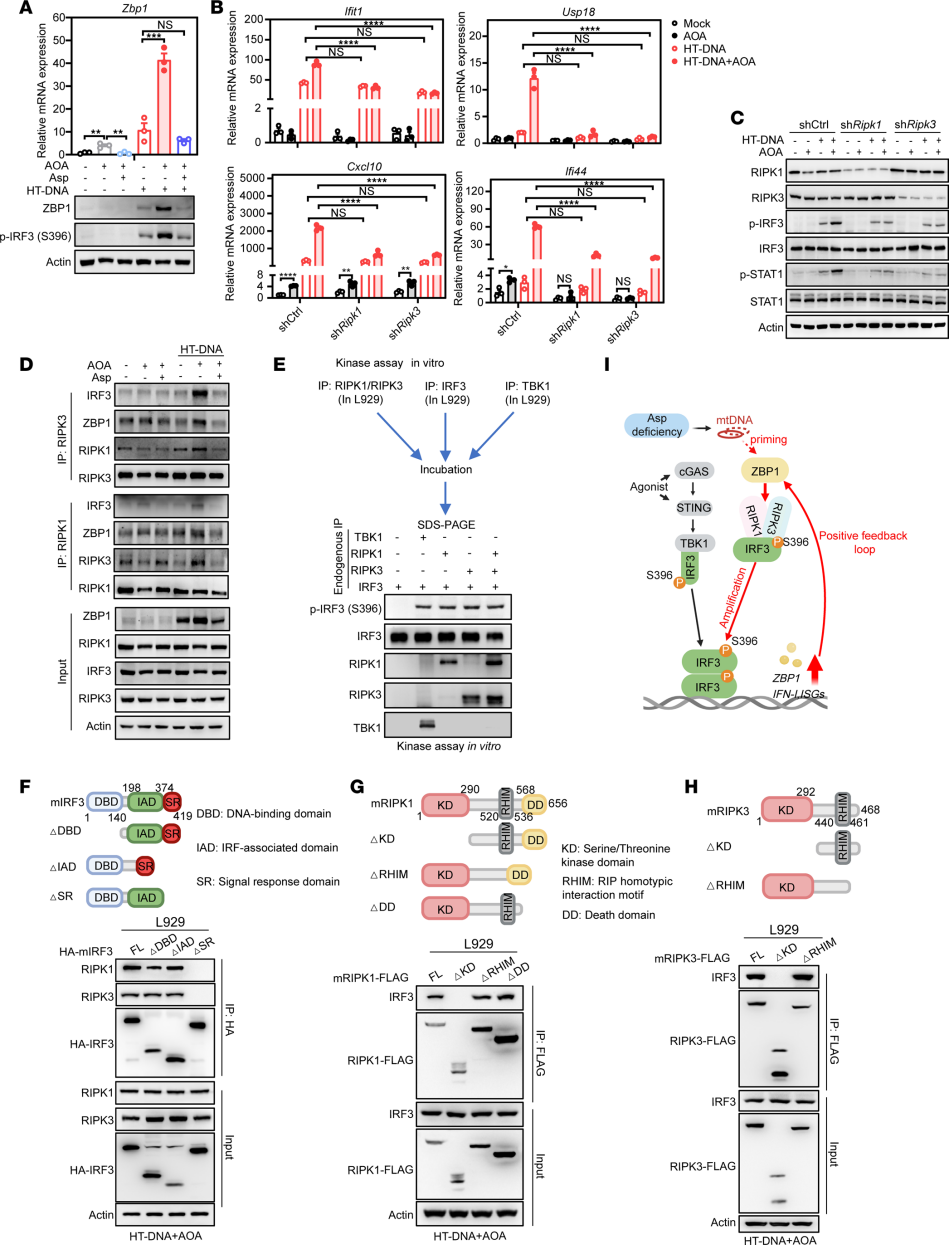
AOA prolongs IRF3 phosphorylation via the ZBP1–RIPK1/3 axis. (**A**) L929 cells were treated with 0.5 mM AOA for 1 hour followed by HT-DNA stimulation in the absence or presence of aspartate, and then cells were harvested for qPCR analysis of *Zbp1* gene expression (top) or Western blot for detecting p-IRF3 levels. (**B**) qPCR analysis of Scr and *Ripk1*- or *Ripk3*-knockdown L929 cells with indicated treatment. (**C**) Western blot of Scr and *Ripk1*- or *Ripk3*-knockdown L929 cells with indicated treatment. (**D**) Endogenous co-IP in L929 cells with indicated treatment. (**E**) An in vitro kinase assay was performed by purified indicated proteins from L929 cells, revealing that RIPK1/3 directly phosphorylated S396 residues of IRF3. (**F**–**H**) Schematic representation of mouse IRF3 (**F**), mouse RIPK1 (**G**), mouse RIPK3 (**H**) full-length and truncations (top). Co-IP showing interactions between mouse IRF3 (**F**) and mouse RIPK1 (**G**) or mouse RIPK3 (**H**) full-length and truncations in L929 cells upon HT-DNA+AOA stimulation (bottom). (**I**) The proposed model: aspartate deficiency–induced mtDNA release initiates the ZBP1–RIPK1/RIPK3 module to prolong IRF3 phosphorylation at Ser396. Statistical analysis was performed by 1-way ANOVA (**A**) or 2-way ANOVA followed by Tukey’s test (**B**). *P < 0.05; **P < 0.01; ***P < 0.001; ****P < 0.0001.

Synergistic response to mtDNA and pathogen-associated molecular patterns has been well elucidated, but the mechanism is unclear. Notably, we observed a significant increase in the expression of another nucleic acid sensor, ZBP1, upon agonist stimulation in AOA-pretreated cells, which was abolished when aspartate was supplemented (Figure 5A). To test whether ZBP1 promotes agonist-mediated ISG production during AOA-induced aspartate deficiency, we deleted ZBP1 in L929 cells. Remarkably, loss of ZBP1 abolished ISG amplification by AOA (Supplemental Figure 9A). ZBP1 deletion also completely abrogated the amplification effect of AOA on the phosphorylation of IRF3^S396^ and STAT1^Y701^ (Supplemental Figure 9B), without affecting the normal activation of the cGAS-STING pathway by agonists (Supplemental Figure 9, A and B), consistent with previous reports (33).

Although TBK1 and IRF3 are the common downstream effectors in the cGAS-STING pathway for IFN-I production, we noticed that AOA-induced aspartate deficiency specifically affected the phosphorylation of IRF3 but not TBK1, implying that other kinases may be involved in prolonging IRF3 phosphorylation (Supplemental Figure 1, H–J). Two serine/threonine kinases, RIPK1 and RIPK3, are established downstream targets of ZBP1 (33, 34). Remarkably, knockdown of either RIPK1 or RIPK3 abolished AOA-mediated amplification of agonist-stimulated ISG production (Figure 5B). Similarly, pretreatment with a selective RIPK1 inhibitor (Necrostatin-1) or RIPK3 inhibitor (GSK′872) also prevented the impact of AOA (Supplemental Figure 9C). Moreover, we observed that knockdown of RIPK1 or RIPK3 was sufficient to block AOA-mediated amplification of IRF3^S396^ phosphorylation (Figure 5C). We therefore focused on how RIPK1/3 regulated IRF3 activation for ISG production in response to multiple stimuli. Interestingly, endogenous immunoprecipitation (IP) revealed an interaction between IRF3 and RIPK1/3 upon the combination of AOA and HT-DNA treatment (Figure 5D and Supplemental Figure 9D). In vitro kinase assays revealed that RIPK1 and RIPK3 can directly phosphorylate IRF3 at Ser396, with TBK1 serving as a positive control (Figure 5E). We further conducted comprehensive domain-mapping experiments on both RIPK1/3 and IRF3 (Figure 5, F–H). Our results demonstrated that the signal response domain of IRF3 and the kinase domain of RIPK1/3 are essential for their interaction (Figure 5, F–H). These data indicate that along with agonists, mtDNA upregulates ZBP1, which recruits RIPK1/3 to phosphorylate IRF3 at Ser396, prolonging its activation and creating a positive feedback loop to amplify the cGAS-STING response via the ZBP1–RIPK1/3 axis (Figure 5I).

ZBP1 is an established initiator of necroptosis driven by RIPK3 and mixed lineage kinase domain-like pseudokinase (MLKL), an inflammatory form of cell death that can be triggered by IFN, subsequently amplifying IFN-I and pro-inflammatory reactions through positive feedback loops (35). However, MLKL knockdown did not affect the impact of AOA treatment (Supplemental Figure 9, E and F), ruling out the role of MLKL-mediated necroptosis in cGAS-STING amplification. This indicates that in the absence of aspartate, the ZBP1–RIPK1/3 axis, which prolongs IRF3^S396^ phosphorylation, amplifies ISG expression independently of MLKL-mediated necroptosis. Taken together, the mtDNA–ZBP1–RIPK1/3–IRF3 axis is critical for the amplification of agonist-stimulated IFN production during AOA-induced aspartate deficiency.

### AOA augments low-dose cGAMP–mediated antitumor immunity.

**Figure 6:**
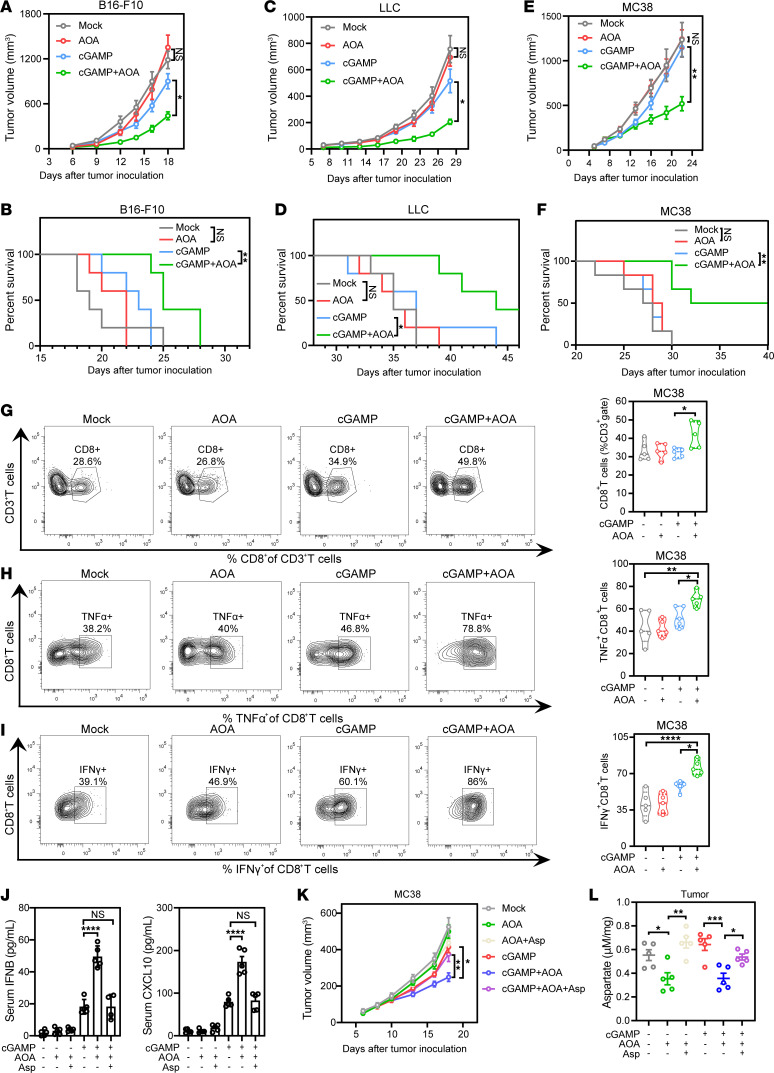
AOA augments low-dose cGAMP–mediated antitumor immunity. (**A**–**F**) Tumor volume (**A**, **C**, **E**) and Kaplan-Meier survival curves (**B**, **D**, and **F**) for C57BL/6 mice inoculated with approximately 2 × 10^5^ B16-F10 cells (**A**, *n* = 5), 2 × 10^5^ LLC cells (**C**, *n* = 5), or 8 × 10^5^ MC38 cells (**E**, *n* = 6). Mice were treated with 3 μg/mouse cGAMP combined with daily injections of AOA (5 mg/kg, i.p.) or PBS on days 7, 10, and 13. (**G**) AOA enhances cGAMP-mediated antitumor response. Quantification analysis of the percentage of CD8^+^ T cells in gated CD3^+^ T cells. (**H** and **I**) TNF-α (**H**) and IFN-γ (**I**) production on CD8^+^ T cells isolated from tumors of MC38 tumor–bearing mice. FACS analysis was performed 6 hours after the last cGAMP injection (*n* = 5). (**J**) MC38 tumor–bearing mice were administered Asp (100 mg/kg) on days 7–13 by intraperitoneal injection, with cGAMP and AOA treatment as mentioned before. Serum IFNB and CXCL10 levels were measured 6 hours after the last cGAMP injection (*n* = 4–5). (**K** and **L**) MC38 cells were subcutaneously transplanted into C57BL/6J mice (*n* = 5). AOA (5 mg/kg) and aspartate (100 mg/kg) were administered intraperitoneally daily along with 3 doses of cGAMP as mentioned above. Tumor volumes (**K**) were measured every 2–3 days, and intratumoral aspartate levels (**L**) were quantified. Data are represented as means ± SEM. Representative data are shown from 2 or 3 independent experiments. Statistical analysis was performed by 1-way ANOVA followed by Tukey’s or Bonferroni’s test (**A**, **C**, **E**, and **G**–**L**) or log-rank test (**B**, **D** and **F**). *P < 0.05; **P < 0.01; ***P < 0.001; ****P < 0.0001.

The cGAS-STING pathway offers significant therapeutic advantages, through the innate immune system and an antitumor immune response (36, 37). However, the transient nature of STING-mediated IFN signaling often requires high-dose administration of STING agonists to achieve an optimal response (36, 38, 39). In addition, tumor cells with low expression of STING, such as MC38 cells, have poor response to immunotherapy targeting the cGAS-STING pathway (40, 41). Since AOA can amplify and prolong STING signaling, we hypothesized that treatment with AOA could enhance the antitumor effects of cGAMP, a STING agonist that sensitizes immunotherapy by targeting the cGAS-STING pathway. To test this hypothesis, we established B16-F10, LLC, and MC38 tumor models and found that the combination of a low dose of cGAMP, which exhibits only a weak antitumor effect at this concentration, and AOA had a substantial inhibitory effect on tumor growth and significantly extended the survival of tumor-bearing mice (Figure 6, A–F). As AOA alone only slightly induced ISG expression in vitro with undetectable phosphorylation of IRF3 or STAT1 (Figure 1), we did not observe a significant inhibitory effect of AOA alone on tumor growth in the 3 subcutaneous tumor models (Figure 6, A, C, and E). Importantly, we observed no significant changes in the body weight of the treated mice, indicating that AOA treatment was well tolerated (Supplemental Figure 10, A–C).

To gain insights into the immune response involved in the suppression of tumor growth, we examined the proportion of CD8^+^ cytotoxic T cells in the tumor microenvironment (TME) (Supplemental Figure 10D). The combination of AOA and cGAMP significantly increased the percentage of CD8^+^ T cells among tumor-infiltrating lymphocytes in both MC38 and B16-F10 tumor models (Figure 6G and Supplemental Figure 10E). Moreover, intracellular cytokine staining of CD8^+^ T cells stimulated with PMA and ionomycin indicated that a higher proportion of tumor-infiltrating CD8^+^ T cells in the combination treatment group expressed IFN-γ and TNF-α, 2 key T cell effector molecules, compared with the cGAMP alone group (Figure 6, H and I, and Supplemental Figure 10F). Consistently, AOA administration alone did not induce significant changes in the infiltration of activated CD8^+^ T cells (Figure 6, G–I, and Supplemental Figure 10, E and F). Thus, AOA augments cGAMP-mediated antitumor immunity in vivo. Consistent with aspartate deficiency as the mechanism of action for AOA-enhanced antitumor activity of cGAMP, AOA-increased, cGAMP-induced secretion of IFNB and CXCL10 was reversed by aspartate administration in vivo (Figure 6J). Most importantly, the administration of aspartate dramatically attenuated the tumor suppression effect of the combination treatment in MC38 tumor model (Figure 6K) and reversed downregulated aspartate production induced by AOA in the tumors (Figure 6L). These findings collectively suggest that AOA can enhance the antitumor immunity of low-dose cGAMP, which is counteracted by aspartate supplementation.

To determine whether AOA directly affects CD8^+^ T cells, we employed complementary in vitro and in vivo approaches. CD8^+^ T cells isolated from AOA-treated mice exhibited intracellular aspartate levels comparable to those from control mice (Supplemental Figure 11, A–C). Consistently, AOA treatment of cultured CD8^+^ T cells failed to affect intracellular aspartate abundance, CD69 expression, or IFN-γ and TNF-α production (Supplemental Figure 11, D–I), indicating that CD8^+^ T cells are intrinsically resistant to AOA-mediated aspartate depletion. In contrast, tumor cells display elevated anabolic and nucleotide demands and are therefore more sensitive to perturbations in aspartate metabolism, whereas CD8^+^ T cells retain greater metabolic flexibility, as previously reported (42). Supporting this model, CD8^+^ T cells exposed to conditioned medium from AOA- and cGAMP-cotreated tumor cells showed enhanced activation and IFN-γ production (Supplemental Figure 12, A–D). Importantly, the enhanced antitumor effects of AOA were largely abolished in CD8^+^ T cell–depleted mice (Supplemental Figure 12, E and F), indicating that CD8^+^ T cell–mediated immunity is required for its therapeutic efficacy. In parallel, we evaluated the antitumor efficacy of cGAMP+AOA in interferon alpha and beta receptor subunit 1–deficient (*Ifnar1*-deficient) mice, as well as in mice treated with a blocking antibody against IFNAR1 (Supplemental Figure 12, G–J). In both settings, the antitumor effects of cGAMP+AOA were significantly impaired, as reflected by increased tumor growth and reduced survival (Supplemental Figure 12, G–J), demonstrating a critical requirement for IFN-I signaling. Collectively, these data clearly support a model in which AOA primarily targets tumor cells, leading to aspartate depletion and induction of IFN-I responses, which in turn indirectly enhance CD8^+^ T cell activation and function.

### cGAS–ZBP1–RIPK1/3 axis and GOT2/GPT2 are essential for cGAMP+AOA–induced antitumor immune response.

**Figure 7:**
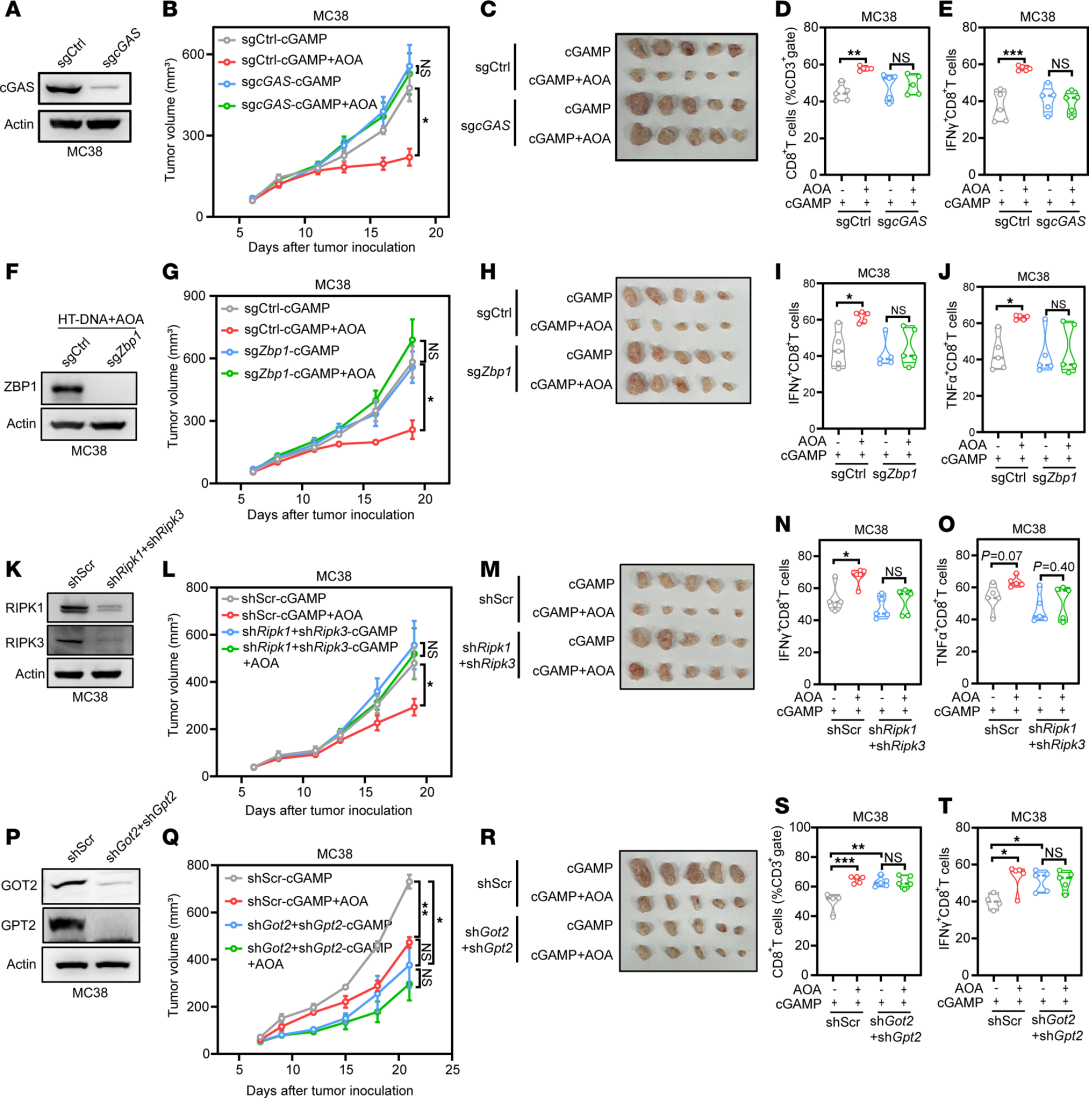
cGAS–ZBP1–RIPK1/3 axis and GOT2/GPT2 are essential for cGAMP+AOA–induced antitumor immune response. (**A**) MC38 cells were transduced with control sgRNA or sgRNA targeting *cGAS*. Whole cell lysates (WCLs) were analyzed by immunoblotting with the indicated antibodies. (**B** and **C**) MC38 cells as described in **A** were subcutaneously transplanted into C57BL/6J mice and administered according to the protocol mentioned above. Tumor volume (**B**) and tumor photos (**C**) were recorded. (**D** and **E**) MC38 cells described in **A** were subcutaneously transplanted into C57BL/6J mice and administered according to the protocol mentioned above. The percentage of CD8^+^ T cells in gated CD3^+^ T cells (**D**) and IFN-γ production (**E**) on CD8^+^ T cells. (**F**) MC38 cells were transduced with control sgRNA or sgRNA targeting *Zbp1*. WCLs were analyzed by immunoblotting with the indicated antibodies. (**G** and **H**) MC38 cells as described in **F** were subcutaneously transplanted into C57BL/6J mice and administered according to the protocol mentioned above. Tumor volume (**G**) and tumor photos (**H**). (**I** and **J**) MC38 cells described in **G** were subcutaneously transplanted into C57BL/6J mice and administered according to the protocol mentioned above. The production of IFN-γ (**I**) and TNF-α (**J**) on isolated CD8^+^ T cells. (**K**) MC38 cells were transduced with control shRNA or shRNA targeting *Ripk1*, and the stable cells were further transduced with control shRNA or sh*Ripk3* to generate double-knockdown (double-KD) cells. WCLs were analyzed by immunoblotting with the indicated antibodies. (**L** and **M**) MC38 cells described in **K** were subcutaneously transplanted into C57BL/6J mice and administered according to the protocol mentioned above. Tumor volume (**L**) and tumor photos (**M**). (**N** and **O**) The production of IFN-γ (**N**) and TNF-α (**O**) on isolated CD8^+^ T cells. (**P**) MC38 cells were transduced with control shRNA or shRNA targeting *Got2*, and the stable cells were further transduced with control shRNA or sh*Gpt2* to generate double-KD cells. WCLs were analyzed by immunoblotting with the indicated antibodies. (**Q** and **R**) MC38 cells described in **P** were subcutaneously transplanted into C57BL/6J mice and administered according to the protocol above. Tumor volume (**Q**) and tumor photos (**R**). (**S** and **T**) The percentage of CD8^+^ T cells in gated CD3^+^ T cells (**S**) and IFN-γ production (**T**) on CD8^+^ T cells. Statistical analysis was performed by 2-way ANOVA and Tukey’s or Bonferroni’s test (**B**–**T**). *P < 0.05; **P < 0.01; ***P < 0.001.

In vitro experiments have demonstrated that AOA-induced mtDNA release to activate cGAS serves as a priming event for augmenting agonist-mediated innate immune responses. We sought to investigate whether AOA similarly enhances low-dose cGAMP–mediated antitumor immune responses in a cGAS-dependent manner. To test this, we established engrafted mouse models using MC38 cells with deletion of cGAS (Figure 7A), and we observed that loss of cGAS in tumor cells significantly diminished the ability of AOA to enhance tumor growth suppression of low-dose cGAMP (Figure 7, B and C). To gain insight into this observation, we performed immune phenotyping to detect the presence of tumor-infiltrating immune cells. Consistently, the combined administration of AOA and cGAMP enhanced CD8^+^ T cell infiltration and activation within tumors (Figure 7, D and E). However, this enhancement was abolished upon cGAS knockout (Figure 7, D and E), indicating that cGAS is essential for AOA-mediated augmentation of low-dose cGAMP–induced antitumor immune responses. To further investigate the involvement of the ZBP1–RIPK1/3 axis in vivo, we generated ZBP1-knockout and RIPK1/3 double-knockdown MC38 cells, respectively (Figure 7, F and K). Similar to the cGAS-knockout data above, loss of either ZBP1 or RIPK1/3 in tumor cells could attenuate the inhibitory effect of AOA combined with cGAMP on tumor growth, accompanied by a decrease in CD8^+^ T cell activity (Figure 7, G–J and L–O). These results further support our conclusion that the ZBP1–RIPK1/3 axis mediates the role of AOA in potentiating the antitumor effect of low-dose cGAMP.

Since we observed that AOA promoted ISG expression mainly by targeting GOT2 and GPT2, we hypothesized that the double knockdown of *Got2* and *Gpt2* might recapitulate the augmentation of the antitumor efficacy of low-dose cGAMP observed with AOA treatment. We generated stable MC38 cells with double knockdown of *Got2* and *Gpt2* and found that depletion of these 2 metabolic enzymes mimicked the effect of AOA, enhancing the antitumor effect of low-dose cGAMP in vivo (Figure 7, P–R). This effect was associated with increased infiltration and activation of CD8^+^ T cells (Figure 7, S and T). Notably, treatment of AOA and low-dose cGAMP did not further suppress tumor cell growth in the presence of GOT2 and GPT2 depletion (Figure 7Q), suggesting that AOA potentiates the antitumor effect of low-dose cGAMP primarily by inhibiting GOT2 and GPT2 in tumor cells. Taken together, our study reveals that tumor cell–intrinsic cGAS, ZBP1–RIPK1/3 signaling axis, and GOT2/GPT2 are essential for the antitumor immune response by the combined treatment of cGAMP and AOA.

### AOA synergizes with chemotherapy-mediated antitumor immunity.

**Figure 8:**
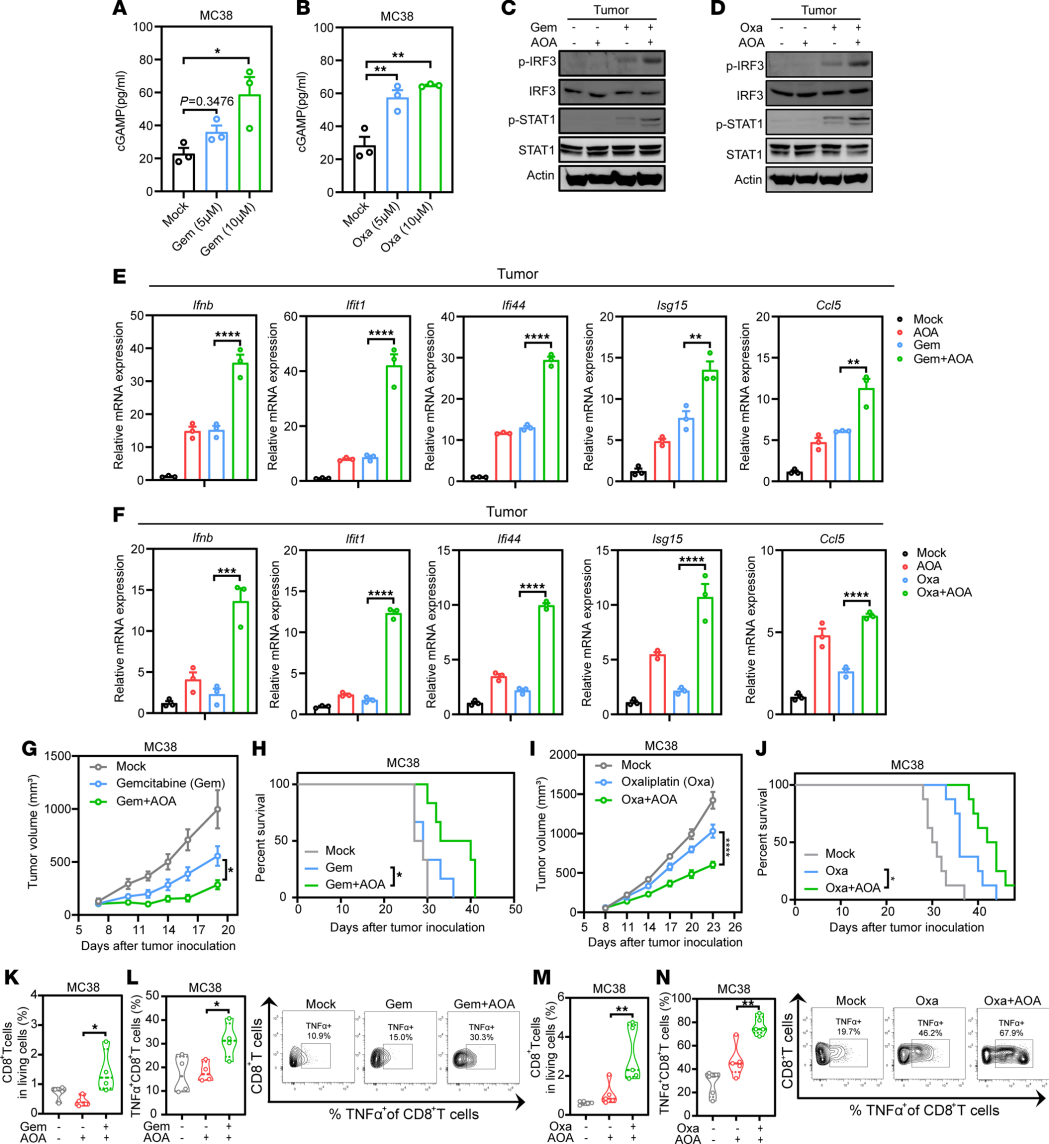
AOA synergizes with chemotherapy-mediated antitumor immunity. (**A** and **B**) Quantitative analysis of intracellular cGAMP in MC38 cells with or without gemcitabine or oxaliplatin treatment by ELISA. (**C** and **D**) MC38 tumor tissues were treated with gemcitabine (**C**) or oxaliplatin (**D**) combined with or without AOA for 24 hours, and the tissular protein expression of p-IRF3 and p-STAT1 was then detected by immunoblotting. (**E** and **F**) MC38 tumor tissues were treated as in **C** and **D** and the tissular mRNA expression of *Ifnb*, *Ifit1*, *Ifi44*, *Isg15*, and *Ccl5* was detected. (**G** and **H**) Tumor volume (**G**) and Kaplan-Meier survival curves (**H**) for C57BL/6 mice inoculated with approximately 8 × 10^5^ MC38 cells. Mice were treated with gemcitabine (50 mg/kg, i.p.) combined with daily injections of AOA (5 mg/kg, i.p.) or PBS on days 7, 10, 13, and 16 (*n* = 6). (**I** and **J**) Tumor volume (**I**) and Kaplan-Meier survival curves (**J**) for C57BL/6 mice inoculated with approximately 8 × 10^5^ MC38 cells. Mice were treated with oxaliplatin (5 mg/kg, i.p.) combined with daily injections of AOA (5 mg/kg, i.p.) or PBS on day 8 and 12. Tumor volume and mouse survival were measured 2–3 times a week (*n* = 8). (**K**–**N**) MC38-bearing mice were treated as in **G** and **I**; lymphocyte infiltration was measured by FACS. Quantification analysis of the percentage of CD8^+^ T cells in living cells from MC38 tumors after indicated treatment (**K** and **M**) (*n* = 4–5). Representative data and quantification analysis of the percentage of TNF-α^+^CD8^+^ T cells from MC38 tumors after indicated treatment (**L** and **N**) (*n* = 4–5). Statistical analysis was performed by 1-way ANOVA followed by Tukey’s test (**A**, **B**, **E**–**G**, **I**, and **K**–**N**) or log-rank test (**H** and **J**). *P < 0.05; **P < 0.01; ***P < 0.001; ****P < 0.0001.

Several chemotherapy drugs have been reported to induce the damage and leakage of nuclear DNA in tumor cells, which are sensed by cGAS, thereby triggering low-level cGAMP production (43, 44). However, the resulting immune activation is often modest. Given our finding that AOA significantly enhances the antitumor effect of low-dose cGAMP, we propose that coadministration of AOA with chemotherapy may represent an effective strategy to amplify antitumor immune responses while minimizing chemotherapeutic toxicity. Gemcitabine (Gem) and Oxaliplatin (Oxa) are FDA-approved clinical first-line chemotherapy drugs and have been reported to activate the cGAS-STING pathway (43, 44). Consistent with previous studies, we found that treating MC38 cells with Gem or Oxa produced cGAMP in a dose-dependent manner (Figure 8, A and B). Next, we tested whether combination treatment could further enhance the activation of the cGAS-STING pathway induced by Gem or Oxa. Fresh tumor tissues were cut into pieces (4–6 mm) in a sterile plate and were subjected to the combination treatment with gemcitabine or oxaliplatin and AOA. As expected, the phosphorylation levels of IRF3 and STAT1 were upregulated in the combination group (Figure 8, C and D). As a consequence, the mRNA expression of *Ifnb*, *Ifit1*, *Ifi44*, *Isg15*, and *Ccl5* was also increased markedly, indicating the superior immunostimulatory activity of the combination group (Figure 8, E and F). To determine whether AOA synergized with chemotherapy drugs in vivo, we gave MC38 tumor–bearing mice combined treatment. Intraperitoneal injection of the chemotherapy drugs markedly retarded the growth rate of MC38-formed tumors (Figure 8, G–J). Intriguingly, the combination treatment with Gem or Oxa and AOA further impaired the growth of MC38-formed tumors and significantly prolonged the survival of mice (Figure 8, G–J). In agreement, after combined treatment, both CD8^+^ T cell infiltration and functional state were further improved (Figure 8, K–N). We next employed the azoxymethane (AOM)/dextran sodium sulfate (DSS) colorectal cancer model to evaluate whether AOA enhances chemotherapy-induced antitumor responses in an orthotopic and immunocompetent setting (Supplemental Figure 13, A and B). Oxa treatment showed a trend toward reduced colon tumor burden, as evidenced by decreased macroscopic tumor numbers and sizes (Supplemental Figure 13, C and D). Notably, combined treatment with Oxa and AOA significantly suppressed tumor growth compared with the control group and appeared more effective than either monotherapy (Supplemental Figure 13, C and D). Tumors from mice receiving the combination therapy exhibited significantly increased frequencies of IFN-γ– and TNF-α–producing CD8^+^ T cells, indicating enhanced cytotoxic T cell functionality within the TME (Supplemental Figure 13, E and F). Together, these results demonstrate that AOA synergizes with chemotherapy to potentiate antitumor immune responses.

To extend these findings beyond chemotherapy, we next assessed whether AOA also synergized with ionizing radiation (IR), a STING-dependent cancer treatment modality (41). First, AOA markedly enhanced IR-induced expression of IFN-I and ISGs, including *Ifnb*, *Ifit1*, *Ccl5*, and *Cxcl10*, at both 24 and 48 hours (Supplemental Figure 14A). Consistent with these molecular changes, combined AOA and local IR treatment significantly suppressed tumor growth and prolonged survival compared with either treatment alone, without additional body weight loss (Supplemental Figure 14, B–D). Collectively, these in vitro and in vivo data indicate that AOA broadly potentiates cGAS-STING pathway activation and antitumor immune responses in the context of cancer treatment, including chemotherapy and radiotherapy.

Given the promising results of cotargeting the aspartate/pyrimidine synthesis pathway and cGAS-STING signaling in mouse tumor models, we investigated whether a similar regulatory mechanism operated in human cancers using data from The Cancer Genome Atlas (TCGA) database. To this end, we assessed the average mRNA expression level of key metabolic genes encoding enzymes in the aspartate-related pyrimidine synthesis pathway, including *GOT2*, *GPT2*, *CAD*, *DHODH*, and *UMPS*, an indication of pathway activity. Our analysis revealed a negative correlation between the activity of aspartate-related pyrimidine synthesis pathway and the expression of ISGs (e.g., *cGAS*, *STING1*, *IRF3*, and *ISG15*) in kidney renal clear cell carcinoma (*n* = 524), kidney renal papillary cell carcinoma (*n* = 284), and liver hepatocellular carcinoma (*n* = 363) (Supplemental Figure 15A). Furthermore, we observed a negative association between the CD8^+^ T cell fraction and aspartate-related pyrimidine synthesis activity across various types of cancers from TCGA datasets (Supplemental Figure 15B). This suggests that the de novo pyrimidine synthesis pathway may exert an inhibitory effect on cytosolic DNA sensing in human tumors, consequently hindering CD8^+^ T cell infiltration.

To further examine the clinical relevance of this pathway using patient samples, we analyzed a cohort of human colorectal adenocarcinoma (CRC) samples, which revealed variable concentrations of aspartate (Supplemental Figure 15C). IHC analysis of these samples indicated that CRC cases with low aspartate levels exhibited higher levels of T cell infiltration and CD8^+^ T cell infiltration compared with those with high aspartate levels (Supplemental Figure 15, D and E). Moreover, a significant negative correlation was observed between aspartate levels and the presence of T cells, CD8^+^ T cell infiltration, and the activation of CD8^+^ T cells, as indicated by granzyme B expression (Supplemental Figure 15, D and E). These findings underscore the potential therapeutic benefit of targeting aspartate metabolism or pyrimidine synthesis for patients with CRC.

## Discussion

Aspartate is intricately involved in the metabolic adaptations of cancer cells, including nucleotide and protein synthesis, energy production, and redox regulation (45–47). Recent studies have unveiled additional functions of aspartate, expanding our understanding beyond its traditional role in metabolism (10, 48–50). Nevertheless, the relationship between aspartate metabolism and innate immune pathways has remained elusive. In this study, we have provided compelling in vitro and in vivo evidence demonstrating that manipulating the availability of aspartate can markedly amplify the cGAS-STING pathway, leading to sustained upregulation of *ISG* expression. Our results demonstrate that aspartate deficiency disrupts CAD-mediated pyrimidine synthesis, resulting in an increase in mtDNA DSBs. This, in turn, facilitates fragmented mtDNA release into the cytoplasm via VDAC oligomerization, thereby triggering the activation of the innate immune response. mtDNA cooperating with agonist upregulates ZBP1, which recruits RIPK1/3 to phosphorylate IRF3 at Ser396, prolonging its activation and creating a positive feedback loop to amplify the cGAS-STING response via the ZBP1–RIPK1/3 axis. This model highlights how metabolic dysregulation and mitochondrial dynamics influence cGAS-STING pathway activation and immune signaling (Supplemental Figure 15F). Given that the mechanism we identified acts on IRF3, a common downstream effector in various innate immune pathways, further investigation is warranted to confirm and extend the direct phosphorylation of IRF3 by RIPK1/3 across other innate immune pathways.

We propose that the innate immune response triggered by metabolic interventions leading to mtDNA release is mild and reversible. In our model, AOA treatment induces a modest upregulation of ISGs, which is reversible upon aspartate replenishment but insufficient to observe detectable downstream proteins of cGAS-STING pathway, such as phosphorylated IRF3 and phosphorylated STAT1. Furthermore, AOA alone does not elicit significant antitumor immune effects in vivo. This contrasts with studies using more extreme methods to induce mtDNA release, such as DNA repair enzyme knockouts (51, 52) or radiation (53), which cause severe and irreversible DNA damage, resulting in substantial dsDNA accumulation in the cytoplasm and stronger cGAS-STING activation. In contrast, AOA treatment did not induce detectable nDNA damage or apoptosis, thus failing to trigger a strong innate immune response. In summary, aspartate deficiency–induced mtDNA release promotes moderate ISG upregulation, with a weak immune effect, but sensitizes cells to agonists. Similar findings have been reported where ATR kinase inhibition enhanced the IFN response to IR but did not directly induce significant ISG production (54). These results suggest that the innate immune response to mtDNA release is context dependent, with varying immune responses based on the intensity of the external stimulus or stressor. Strong, irreversible stress may elicit a potent innate immune response, but it also causes significant cellular or organismal damage. In contrast, milder, reversible environmental changes, while inducing a weaker innate immune response, can prevent irreversible damage to cells or tissues. Moreover, the generation of this weaker immune response can function akin to an “accelerator” greatly amplifying the effects of immune agonists and the immune responses induced by radiotherapy or chemotherapy, thus providing a dual benefit.

Recent studies have demonstrated that cGAS can physically and functionally associate with ZBP1 to cooperatively sense mtDNA and initiate downstream innate immune signaling (33). Building on this emerging concept, our study further reveals that cGAS and ZBP1 function cooperatively to establish a positive feedback circuit that sustains IRF3 phosphorylation and amplifies IFN-I production. Although cGAMP is downstream of cGAS, cGAS itself plays an essential priming and scaffolding role that enables efficient ZBP1–RIPK1/3–IRF3 signaling. In this model, mtDNA release engages a cooperative cGAS/ZBP1 signaling axis that primes and amplifies STING agonist–driven IRF3 activation, forming a sustained positive feedback loop.

Furthermore, we explored the potential involvement of mitochondrial permeability transition pore (mPTP) opening in our experimental settings. Pharmacological inhibition of mPTP with cyclosporin A attenuated the AOA-mediated enhancement of ISG expression, suggesting that mPTP activity may at least in part contribute to this process (data not shown). However, we did not detect a measurable increase in global mPTP opening upon AOA treatment. This discrepancy raises the possibility that AOA induces low-level or transient (“flickering”) mPTP activity that falls below the detection limit of current assays (55). Our study does have certain limitations. While our study supported that RIPK1/3 can directly phosphorylate IRF3 in the context of agonist and mtDNA synergy, we have yet to clarify the temporal sequence and predominant roles of TBK1 and RIPK1/3 in modulating IRF3. Additionally, while double knockdown of GOT2 and GPT2 further reduces aspartate, the compensatory mechanisms underlying this metabolic alteration are yet to be elucidated. Although the negative correlation between the aspartate levels and infiltration of CD8^+^ T cells is observed in human CRC samples with treatment naivety, whether the correlation implies that all those tumors should have a better response to chemotherapy needs to be further investigated. Furthermore, whether cancer patients with impaired de novo synthesis pathways of aspartate and pyrimidine will benefit from anti–programmed cell death ligand 1 immunotherapy remains an important and unresolved question.

## Methods

### Sex as a biological variable.

In this study, sex was not considered as a biological variable in the animal experiments. In human tumor tissue samples studies, sex was not considered as a biological variable.

### In vivo animal experiments.

Six- to 8-week-old C57BL/6J mice were purchased from Beijing Vital River Laboratory Animal Technology Co., Ltd, and age-matched *Ifnar1*-KO C57BL/6J mice were obtained from Cyagen Biosciences (Suzhou) Inc. BJ-5ta and L929 cells were obtained from ATCC, while B16-F10 and MC38 cells were purchased from the National Collection of Authenticated Cell Cultures. A total of 2 × 10^5^ B16-F10 or 8 × 10^5^ MC38 cells resuspended in PBS were injected into the shaved flanks subcutaneously to establish tumors. At 6 or 7 days after tumor inoculation, mice were treated with 2′3′-cGAMP/Gem/Oxa and/or AOA as previously described. For CD8^+^ T cell depletion experiments, mice received intraperitoneal injections of anti-CD8–depleting antibody (Bio X Cell, BE0223; 10 mg/kg) or isotype control on days 0, 3, 6, 9, and 12. For IFNAR1 blockade, mice were treated with an anti-IFNAR1 antibody (Bio X Cell, BE0241) intraperitoneally according to the indicated schedule. For in vivo irradiation, mice were anesthetized and shielded with lead plates, with only the subcutaneous tumor area exposed to receive 6 Gy irradiation using a Cs-137 γ-irradiator (Gammacell 40 Exactor). Tumor volumes were measured every 2–3 days with a digital caliper and were calculated using the following formula: length × width × width/2. Body weight was weighed every 2–3 days. Mice were sacrificed when the tumor size reached 2,000 mm^3^. For the AOM/DSS-induced colorectal tumor model, mice received an intraperitoneal injection of 8 mg/kg AOM followed by 3 cycles of DSS in drinking water for 7 days and regular water for 14 days. Mice were treated with Oxa or/and AOA according to the indicated schedule.

### RNA-seq.

L929 cells were treated as mentioned above, and total RNA from each sample was extracted using TRIzol Reagent (Invitrogen). Both quality and concentration of RNA samples were detected with a NanoDrop 2000 (Thermo Fisher Scientific) and Agilent 2100 Nano. Qualified RNA samples were used for RNA-seq (Majorbio). R software (R Foundation for Statistical Computing) was used for data analysis. RNA-seq data generated in this paper were deposited at the NCBI Gene Expression Omnibus (accession number GSE276917).

### Metabolite extraction and targeted metabolomics analysis.

The L929 cells were plated in 6 cm dishes at equal densities and grown to 60%–70% confluence in log-phase in the cell culture plates. Then, L929 cells were treated with [U-13C]-aspartate/aspartate (Cambridge Isotope Laboratories/Sigma) and/or AOA for 1 hour followed by HT-DNA transfection for 6 hours. Cells were washed twice with 1 mL of cold PBS (Meilunbio), and intracellular metabolites were extracted by adding 80% methanol (MeOH: H_2_O = 4/1, v/v, 500 μL).

The extraction was centrifuged for 20 minutes at 12,000*g* and 4°C to precipitate the insoluble materials. The supernatant was transferred to a new 1.5 mL Eppendorf tube and evaporated to dryness with a vacuum concentrator at 4°C. The dried extracts were resuspended in acetonitrile/water (1:1, v/v), sonicated for 5 minutes, and centrifuged for 20 minutes at 12,000*g*, and we subjected the supernatant to LC–MS/MS on a QTRAP 6500+ mass spectrometer coupled to an ExionLC system (AB SCIEX). Chromatographic separation was performed on a Waters Corporation XBridge Amide column (particle size, 3.5 μm; 150 mm × 4.6 mm). The injected volume was 5 μL. The mobile phase consisted of water (A) and acetonitrile/water (95:5; B), and the gradient elution was set as follows: 0.0–3.0 minutes, 85% B; 3.0–12.0 minutes, 85%–30% B; 12.0–15.0 minutes, 30%–2% B; 15.0–16.0 minutes, 2% B; 16.0–23 minutes, 85% B. The flow rate was 0.45 mL/min and column temperature 40°C. Metabolites were detected by electrospray ionization using multiple reaction monitoring in positive and negative ion mode. The MS parameters were as follows: 5,500 V of capillary voltage, 80 V of declustering potential. Analyst 1.6.3 (AB SCIEX) software was used for data acquisition and analysis.

### Flow cytometry.

Tumors were harvested 6 hours after 2′3′-cGAMP injection and cut into small pieces with ophthalmic scissors in 1 mL DMEM containing 10% FBS, digested with DNase I (0.2 mg/mL; Sigma-Aldrich, catalog DN25) and collagenase I (1 mg/mL; Worthington Biochemical, catalog LS004196) in cell culture incubator for 20 minutes, and filtered with 70 μm cell strainers (BD Biosciences, now Waters Biosciences, 352340) to prepare single-cell suspensions followed by incubating PMA/ionomycin (Invitrogen 4975) for 3 hours. Briefly, cells were blocked with anti-FcR (CD32/16 blocker) (BioLegend, catalog 101320) at 4°C for 30 minutes and washed once with FACS buffer (PBS containing 2% FBS). Cell surface markers were stained with antibody cocktail against anti–CD45-PB (BioLegend, catalog 103126), anti–CD3-APC (BioLegend, catalog 100236), anti–CD8a-V500 (BD Biosciences, catalog 560776), anti–CD69-PE-Cy5 (BioLegend, catalog 104510), and live/dead (Fixable Viability Stain 780, BD Biosciences, catalog 565388) at 4°C for 30 minutes. Cells were then fixed with fixation/permeabilization buffer (BD Biosciences, catalog 554714) at 4°C for 20 minutes and washed twice with permeabilization wash buffer (BD Biosciences, catalog 554714) followed by intracellular staining with anti–IFNγ-BV650 (BioLegend, catalog 505832) and anti–TNFα-PE-D594 (BioLegend, catalog 506346) at 4°C for 1 hour. The stained cells were analyzed using a BD FACSCelesta and further analyzed with FlowJo software.

### Statistics.

Experiments were repeated 2 to 3 times. No statistical method was used to predetermine sample size. The figure legends contain the statistical details for each experiment. Statistical analyses were performed using GraphPad Prism (8.0). Data were presented as mean values ± SEM. Statistical significance was determined by considering a significance level of *P* < 0.05. For all mouse studies, the assignment of mice to treatment groups was done randomly. No data were excluded from the analyses. The RNA-seq was performed using 3 biological replicates. The metabolite quantification and isotope tracing by LC–MS/MS were performed using 3 biological replicates.

### Study approval.

A statement of informed consent was obtained from the patients with CRC. No compensation was provided. The study protocol was approved by Ethics Committee of Xinhua Hospital Affiliated to Shanghai Jiao Tong University School of Medicine, Shanghai, China. The animal experiments were performed at the Fudan Animal Center in accordance with animal welfare guidelines, and the procedures were approved by the Ethics Committee of the Institutes of Biomedical Sciences, Fudan University.

### Data availability.

The RNA-seq data generated in this study have been deposited in the NCBI Gene Expression Omnibus under accession number GSE276917. Numerical values for all data points shown in the figures are provided in the Supporting Data Values file.

## Author contributions

YHL and HY designed the study. YHL conducted most experiments and drafted the manuscript. HZW and HXL conducted some key experiments and revised the manuscript. RQS, XL, XC, YZZ, and ZQH provided technical assistance. HY, DY, and YM wrote, reviewed, and edited the manuscript. CYL collected the clinical CRC samples. WW assisted in conducting END-seq experiments. ZY performed bioinformatics analysis. YHL, HZW and HXL analyzed data. HY, DY and YM acquired funding and supervised the study. YHL, HZW, and HXL are co–first authors. Author order among the co–first authors was determined by the extent of their contributions.

## Conflict of interest

The authors have declared that no conflict of interest exists.

## Funding support

- National Key Research and Development Program of China (2023YFC3404800 and 2025YFA1805600, HY).
- National Natural Science Foundation of China (82273203 and 82472821, HY).
- The Central Guidance Funds for Local Science and Technology Development (YDZX20233100001003, HY).

## Supplementary Material

Supplemental data

Unedited blot and gel images

Supplemental video 1

Supplemental video 2

Supplemental video 3

Supporting data values

## Version 1 06/01/2026

Electronic publication

## References

[1] Sun L (2013). Cyclic GMP-AMP synthase is a cytosolic DNA sensor that activates the type I interferon pathway. Science.

[2] Liu S (2015). Phosphorylation of innate immune adaptor proteins MAVS, STING, and TRIF induces IRF3 activation. Science.

[3] Wu J (2013). Cyclic GMP-AMP is an endogenous second messenger in innate immune signaling by cytosolic DNA. Science.

[4] Demaria O (2019). Harnessing innate immunity in cancer therapy. Nature.

[5] Fang L (2023). Methionine restriction promotes cGAS activation and chromatin untethering through demethylation to enhance antitumor immunity. Cancer Cell.

[6] Shen L (2021). Serine metabolism antagonizes antiviral innate immunity by preventing ATP6V0d2-mediated YAP lysosomal degradation. Cell Metab.

[7] Birsoy K (2015). An essential role of the mitochondrial electron transport chain in cell proliferation is to enable aspartate synthesis. Cell.

[8] Gui DY (2016). Environment dictates dependence on mitochondrial complex I for NAD+ and aspartate production and determines cancer cell sensitivity to metformin. Cell Metab.

[9] Sullivan LB (2015). Supporting aspartate biosynthesis is an essential function of respiration in proliferating cells. Cell.

[10] Wu B (2021). Mitochondrial aspartate regulates TNF biogenesis and autoimmune tissue inflammation. Nat Immunol.

[11] Doglioni G (2025). Aspartate signalling drives lung metastasis via alternative translation. Nature.

[12] Guo C (2023). SLC38A2 and glutamine signalling in cDC1s dictate anti-tumour immunity. Nature.

[13] Zhang Q (2022). AMPK directly phosphorylates TBK1 to integrate glucose sensing into innate immunity. Mol Cell.

[14] Tanaka Y, Chen ZJ (2012). STING specifies IRF3 phosphorylation by TBK1 in the cytosolic DNA signaling pathway. Sci Signal.

[15] Fitzgerald KA (2003). IKKepsilon and TBK1 are essential components of the IRF3 signaling pathway. Nat Immunol.

[16] McWhirter SM (2004). IFN-regulatory factor 3-dependent gene expression is defective in Tbk1-deficient mouse embryonic fibroblasts. Proc Natl Acad Sci U S A.

[17] Korangath P (2015). Targeting glutamine metabolism in breast cancer with aminooxyacetate. Clin Cancer Res.

[18] Xu T (2023). Reply to: GOT1 constrains T_H_17 cell differentiation, while promoting iT_reg_ cell differentiation. Nature.

[19] Hao Y (2016). Oncogenic PIK3CA mutations reprogram glutamine metabolism in colorectal cancer. Nat Commun.

[20] Xu W (2023). GOT1 constrains T_H_17 cell differentiation, while promoting iT_reg_ cell differentiation. Nature.

[21] Hu Q (2021). Genetically encoded biosensors for evaluating NAD^+^/NADH ratio in cytosolic and mitochondrial compartments. Cell Rep Methods.

[22] Stottrup NB (2010). Inhibition of the malate-aspartate shuttle by pre-ischaemic aminooxyacetate loading of the heart induces cardioprotection. Cardiovasc Res.

[23] Okazaki A (2017). Glutaminase and poly(ADP-ribose) polymerase inhibitors suppress pyrimidine synthesis and VHL-deficient renal cancers. J Clin Invest.

[24] Madala HR (2020). Nitrogen trapping as a therapeutic strategy in tumors with mitochondrial dysfunction. Cancer Res.

[25] Elliott IA (2019). Lysosome inhibition sensitizes pancreatic cancer to replication stress by aspartate depletion. Proc Natl Acad Sci U S A.

[26] Lane AN, Fan TW (2015). Regulation of mammalian nucleotide metabolism and biosynthesis. Nucleic Acids Res.

[27] Ruiz-Ramos A (2016). Structure and functional characterization of human aspartate transcarbamoylase, the target of the anti-tumoral drug PALA. Structure.

[28] Vodicka P (2025). Mitochondrial DNA damage, repair, and replacement in cancer. Trends Cancer.

[29] Riley JS (2018). Mitochondrial inner membrane permeabilisation enables mtDNA release during apoptosis. EMBO J.

[30] McArthur K (2018). BAK/BAX macropores facilitate mitochondrial herniation and mtDNA efflux during apoptosis. Science.

[31] White MJ (2014). Apoptotic caspases suppress mtDNA-induced STING-mediated type I IFN production. Cell.

[32] Kim J (2019). VDAC oligomers form mitochondrial pores to release mtDNA fragments and promote lupus-like disease. Science.

[33] Lei Y (2023). Cooperative sensing of mitochondrial DNA by ZBP1 and cGAS promotes cardiotoxicity. Cell.

[34] Muendlein HI (2022). ZBP1 promotes inflammatory responses downstream of TLR3/TLR4 via timely delivery of RIPK1 to TRIF. Proc Natl Acad Sci U S A.

[35] Jiao H (2020). Z-nucleic-acid sensing triggers ZBP1-dependent necroptosis and inflammation. Nature.

[36] Wang H (2017). cGAS is essential for the antitumor effect of immune checkpoint blockade. Proc Natl Acad Sci U S A.

[37] Yum S (2021). TBK1 recruitment to STING activates both IRF3 and NF-κB that mediate immune defense against tumors and viral infections. Proc Natl Acad Sci U S A.

[38] Corrales L (2015). Direct activation of STING in the tumor microenvironment leads to potent and systemic tumor regression and immunity. Cell Rep.

[39] Gonugunta VK (2017). Trafficking-mediated STING degradation requires sorting to acidified endolysosomes and can be targeted to enhance anti-tumor response. Cell Rep.

[40] Zhang L (2023). STING is a cell-intrinsic metabolic checkpoint restricting aerobic glycolysis by targeting HK2. Nat Cell Biol.

[41] Deng L (2014). STING-Dependent cytosolic DNA sensing promotes radiation-induced type I interferon-dependent antitumor immunity in immunogenic tumors. Immunity.

[42] Leone RD (2019). Glutamine blockade induces divergent metabolic programs to overcome tumor immune evasion. Science.

[43] Ma Z (2023). AhR diminishes the efficacy of chemotherapy via suppressing STING dependent type-I interferon in bladder cancer. Nat Commun.

[44] Wan Z (2023). Overcoming pancreatic cancer immune resistance by codelivery of CCR2 antagonist using a STING-activating gemcitabine-based nanocarrier. Mater Today (Kidlington).

[45] Fu A, Danial NN (2018). Grasping for aspartate in tumour metabolism. Nat Cell Biol.

[46] Garcia-Bermudez J (2018). Aspartate is a limiting metabolite for cancer cell proliferation under hypoxia and in tumours. Nat Cell Biol.

[47] Sullivan LB (2018). Aspartate is an endogenous metabolic limitation for tumour growth. Nat Cell Biol.

[48] Deng L (2020). p53-mediated control of aspartate-asparagine homeostasis dictates LKB1 activity and modulates cell survival. Nat Commun.

[49] Mei X (2021). RIPK1 regulates starvation resistance by modulating aspartate catabolism. Nat Commun.

[50] Qi L (2021). Aspartate availability limits hematopoietic stem cell function during hematopoietic regeneration. Cell Stem Cell.

[51] Hu M (2021). ATM inhibition enhances cancer immunotherapy by promoting mtDNA leakage and cGAS/STING activation. J Clin Invest.

[52] Ghosh M (2023). p53 engages the cGAS/STING cytosolic DNA sensing pathway for tumor suppression. Mol Cell.

[53] Yang Y (2021). ZBP1-MLKL necroptotic signaling potentiates radiation-induced antitumor immunity via intratumoral STING pathway activation. Sci Adv.

[54] Feng X (2020). ATR inhibition potentiates ionizing radiation-induced interferon response via cytosolic nucleic acid-sensing pathways. EMBO J.

[55] Lonobile C (2025). The mitochondrial permeability transition pore in platelets: mechanisms, physiological roles, and therapeutic perspectives. Antioxidants (Basel).

