# Supplementary material for: Aspartate deficiency amplifies cGAS-STING signaling in antitumor immunity
Source: J Clin Invest. 2026 Jun 1;136(11):e199716. doi: 10.1172/JCI199716 (PMC13221234; doi:10.1172/JCI199716)

Supplementary information

Supplementary Methods

Chemicals and metabolites. DMXAA (Selleck S1537), HT-DNA (Sigma), 2'3'-cGAMP (Invivogen 1441190), Digitonin (Sigma D5628), 2-DG (Selleck S4701), Oxamate (Selleck S6871), AOA (Selleck S4989), FHIN (GLPBIO GC32878), DEBM (Sigma 112038), DMM (Sigma 136441), Oligomycin (Selleck S1478), Rotenone (Selleck S2348), Piericidin A (Cayman 15379), BPTES (Selleck S7753), Rapamycin (Selleck S1039), Metformin (Selleck S1950), Dimethyl D-(+)-Malate (TCI, M1347), Dimethyl α-ketoglutarate (Sigma, 349631), Succinate (TCI, S0104), MMF (TCI, S2413), Arginine (Sigma A5006), Citrulline (Sigma C7629), ASA (Argino-succinate) (Sigma A5707), Thymidine (Sigma T1895), ddC (Absin abs811894), VBIT-4 (selleck S3544), Oxaliplatin (selleck S1224), Gemcitabine (selleck S1149), Necrostatin-1 (Selleck S8037), GSK'872 (Selleck S8465).

Cell culture, HT-DNA transfection and DMXAA stimulation. L929 cells, BJ-5ta and RAW264.7 cells were purchased from the ATCC. B16-F10 cells and MC38 cells were contributed by laboratory of M. Luo. HT-1080 and LLC cells were obtained from the Cell Bank of the Chinese Academy of Sciences. These cell lines were grown in DMEM (Meilunbio) containing 10% FBS (Gibco), penicillin (100 U/ml) and streptomycin (100 μg/ml). All cells were maintained at 37 °C and 5% CO_2 and were tested for mycoplasma. Cells were seeded in 6-well plate and HT-DNA was transfected into cells using Lipofectamine 2000 (Thermo Fisher) at a concentration of 0.5μg/ml. Cells were treated with DMXAA at a concentration of 50μM.

RNAi. Cells were seeded at equal densities and grown to confluency over a period of 24 h. Opti-MEM and lipofectamine RNAiMax (Thermo) were used for reverse transfection of short interfering RNA (siRNA) for 48h. The si*GOT2* (5'-GCCTTCACTATGGTCTGCAAA-3') and si*GPT2* (5'-CGGCATTTCTACGATCCTGAA-3') were used in this study.

Quantitative real-time PCR (qRT-PCR). Cells were cultured as mentioned above and total RNA was collected in lysis buffer (EZBioscience) and extracted using EZ-press RNA Purification Kit (B0004D) according to the manufacturer's instructions. RNA was subjected to cDNA synthesis using 4× EZscript Reverse Transcription Mix II (with gDNA Remover) (EZB-RT2GQ). The cDNA was diluted 10 times and then performed the real-time qPCR reaction containing SYBR ChamQ Universal SYBR qPCR Master Mix (Vazyme) and gene-specific primers listed in Supplemental Table S1. The reactions were performed in a QuantStudioTM 6 Flex Real-Time PCR System (Applied Biosystems).

Generation of knockdown cell pools. The shRNA sequences targeting *Cad*, *Vdac1/3*, *Ripk1*, *Ripk3* and *Mlkl* were ligated into the plko.1 vector. The shRNA construct was co-transfected with packaging (psPAX2) and envelope (pMD2.G) vectors into HEK293T cells. Lentivirus from supernatant was harvested at 24h and then mixed with 8μg/ml polybrene to infect L929 cells. The stable cells were selected in 10ug/ml puromycin for 2 days.

CRISPR-cas9 mediated gene knockout. sgRNA sequences targeting mouse cGAS, STING, IRF3, ZBP1, BAX and BAK listed in TableS2 were ligated into plasmid lentiCRISPRv2 (Addgene, deposited by Feng Zhang of MIT) digested by BsmbI (NEB). The construct was packaged into a lentivirus. L929 cells were then infected with lentivirus and selected in 10ug/ml puromycin for 2 days.

Western Blot. Cells were cultured in six-well plates under the indicated treatment conditions. After treatment, the culture medium was discarded, and cells were washed with cold PBS. Cells were then lysed with SDS sample loading buffer and boiled at 95 °C for 8 min. Protein samples were separated by SDS–PAGE and transferred onto NC membranes. The membranes were blocked with skim milk at room temperature for 1 h and subsequently incubated with primary (1:1000) and secondary (1:500) antibodies. The immunoreacted products were visualized using ECL reagent and autoradiography by GE Typhoon FLA 9500. Primary antibody lists : STING (D2P2F) (Cell Signaling Cat# 13647) TBK1 (Cell Signaling Cat# 3504) IRF3 (Cell Signaling Cat# 4302) p-TBK1(Ser172) (D52C2) (Cell Signaling Cat# 5483) p-IRF3(Ser396) (D6O1M) (Cell Signaling Cat# 29047) Actin (Proteintech Cat# 66009-1) TOM20 (Novus Cat# NBP1-81556) Gapdh (6C5) (Santa Cruz Cat# sc-32233) Histone H3 (Cell

Signaling Cat# 9715S) Bax (Cell Signaling Cat# 2772) Bak (D4E4) (Cell Signaling Cat# 12105) Stat1 (Cell Signaling Cat# 14994) p-Stat1 (58D6) (Cell Signaling Cat# 9167) Zbp1 (Adipogen Cat# AG-20B-0010) RIP (D94C12) (Cell Signaling Cat# 3493) RIP3 (D4G2A) (Cell Signaling Cat# 95702) MLKL (E7V4W) (Cell Signaling Cat# 37705).

Co-immunoprecipitation. The cytosolic fraction was obtained by lysing the cells with NP-40 lysis buffer (Beyotime). Protein A agarose beads (Millipore 16125) and IRF3, RIPK1/RIPK3 antibodies were used for co-IP from cytosolic fractions. 20μL of sample was mixed with 2× SDS sample loading buffer and boiled at 95°C for 8 minutes. The samples were loaded on SDS-PAGE gels for electrophoresis.

Cellular immunofluorescence. BJ-5ta cells were fixed in 4% Paraformaldehyde (Serivcebio) for 10min at room temperature followed by 3 x 5 minute washes in PBS. Then cells were permeabilized with 0.1% (v/v) Triton X-100 in PBS for 10 minutes at room temperature followed by 3 x 5 minute washes in PBS and blocked in 3% BSA for an hour. Cells were then incubated with corresponding primary antibodies overnight at 4°C. Cells were washed 3 x 5 minute washes in PBS and incubated with secondary antibody for an hour at room temperature followed by 3 x 5 minute washes. To visualize the nuclei, cells were co-stained with DAPI (Sigma D9542). Immunofluorescence pictures were taken by Leica SP8 laser scanning confocal microscope and analyzed by Image J. Primary antibody list: DNA (Millipore #CBL186), TOM20 (Novus Cat# NBP1-81556) IRF3 (D614C) (Cell Signaling Cat# 11904).

NADH/NAD ratio detection. NAD+ levels were determined by using NAD+/NADH assay kit with WST-8 (Beyotime) according to the manufacturer's instructions. In brief, about 1×10^6 cells were added 200 μl of NAD+/NADH extraction solution with a gently pipette, then centrifuged at 12,000g at 4°C for 10 minutes. Supernatant was transferred to a 96-well plate as the sample to measure total NAD+ and NADH. 50-100μl of the sample was heated for 30 minutes in a 60°C water bath and 20μl supernatant was transferred to a 96-well plate as the sample to measure NADH. After adding the alcohol dehydrogenase working solution, samples were incubated at 37°C in the dark for 10 minutes. Then added chromogenic solution to each well and incubated at 37°C in the dark for 30 minutes.

Absorbance at 450 nm was measured and analyzed on a plate reader (BioTek).

SIM-Ultimate two-color imaging. Cells were pretreated with AOA for 6 hours, or with 5μM ABT-737 and 1μM S63845 (Selleck) for 20 minutes. Subsequently, the cells were incubated in DMEM containing SYBR Gold (10,000×) and PK Orange (1,000×) for 15 minutes. After staining, cells were washed three times with PBS and imaged at 37 °C in a humidified incubator with 5% CO_2 using an Olympus SIM-Ultimate microscope.

Apoptotic cell death analysis. Apoptotic cell death was assessed by flow cytometry with FITC-Annexin V (Vazyme, Cat# A211-02) and live/dead (Fixable Viability Stain 780, BD Biosciences, catalog no. 565388). The dilution of the staining reagents was performed according to the manufacturer's instructions.

cGAMP Detection. MC38 tumor cells were treated with gemcitabine or oxaliplatin in 6-well plate, and cultured for 24 hours. Then cells were collected and lysed using MPER™ Mammalian Protein Extraction Reagent (Thermo Scientific, Cat# 78503) at 4°C for 20 min. Then cGAMP in cell lysates was determined using 2'3'-cGAMP ELISA Kit (Cayman Chemical, Cat# 501700) according to standard protocols.

IFN-beta and CXCL10 measurement by ELISA. Cytokine levels in mouse serum were measured with Mouse IFN-beta Quantikine ELISA Kit (Bio-techne) and Mouse IP-10 ELISA Kit (CXCL10) (Abcam) according to the manufacturer's instructions.

Immunohistochemistry. The tissue specimens were fixed overnight in 10% formalin and then were dehydrated in increasing concentrations of ethanol, followed by clearing of alcohol by xylene. The specimens were subsequently embedded in paraffin wax and sectioned continuously at a thickness of 4μm. Hematoxylin and eosin (H&E) staining was performed according to standard protocols. For

immunohistochemistry, the sections were deparaffinized with xylene for 15 min three times and rehydrated in 100%, 85% and 75% ethanol for 5 min. Then, tissue sections were incubated in citrate buffer for antigen retrieval. The sections were then placed into 3% hydrogen peroxide and incubated at room temperature for 25 min to block endogenous peroxidase activity. After three times washing, tissue sections were blocked with 3% BSA for 30min at room temperature and stained with primary antibodies including anti-CD3 (GB111337), anti-CD8 (GB12068) and anti-granzyme B (GB12092) and incubated at 4°C overnight. The sections were then incubated with HRP-conjugated secondary antibodies at room temperature for 1 h. After three washes with PBS, DAB solution (G1211, Servicebio) was added and the slides were counterstained with hematoxylin.

[U-13C]-aspartate tracing analysis. Samples were analyzed by Q Exactive quadrupole orbitrap high resolution mass spectrometry coupled with a Dionex Ultimate 3000 RSLC (HPG) ultra-performance liquid chromatography (UPLC-Q-Orbitrap-HRMS) system (Thermo Fisher Scientific), with a HESI ionization source. The injection volume was 2 μL. Samples were separated with an ACQUITY UPLC BEH Amide column (100 mm × 2.1 mm, 1.7-μm particle size; Waters). The mobile phase consisted of 25 mM ammonium formate and 20 mM ammonium hydroxide in water (A), and acetonitrile (B), the gradient elution was set as follows: 0–1.0 min, 95% B; 1.0–11.0 min, 95%–45% B; 11.0–12.0 min, 45% B; 12.1–17 min, 95% B. The flow rate was 0.3mL/min and column temperature 45°C.

All MS experiments were performed in positive and negative ion modes using a heated ESI source. The source and ion transfer parameters applied were as followed: spray voltage 3.5 kV (positive) 2.8kV (negative). For the ionization mode, the sheath gas, aux gas, capillary temperature and heater temperature were maintained at 40, 10 (arbitrary units), 320 °C and 350 °C, respectively. The S-Lens RF level was set at 50. The Orbitrap mass analyzer was operated at a resolving power of 70,000 in full-scan mode (scan range: 200-1200 m/z; automatic gain control (AGC) target: 1e6) and of 17,500 in the Top 3 data-dependent MS2 mode (stepped normalized collision energy: 10, 20 and 30; injection time: 50 ms; isolation window: 1.2 m/z; AGC target: 1e5) with a dynamic exclusion setting of 5.0 seconds.

Cytosolic mtDNA/mtRNA extraction and detection. As described in Supplementary Figure 7A, 5×10^6 L929 cells were equally divided into two aliquots. One aliquot was boiled with 50 mM NaOH at 95°C for 30 min followed by adding 1 M Tris-HCl (pH 8.0) to neutralize the pH and then centrifuged at 12000 g for 10 min. Another aliquot was resuspended in 300μL buffer containing 50 mM HEPES (pH 7.4), 150 mM NaCl and 20μg/mL digitonin. The resuspensions were incubated at 4°C for 10 min to allow plasma membrane permeabilization and then centrifuged at 980 g for 3 min three times. The cytosolic supernatants were centrifuged at 12000 g for 10 min to avoid remaining cellular debris, yielding cytosolic preparations free of nuclear, mitochondrial and endoplasmic reticulum contamination. DNA was then purified from these pure cytosolic fractions using TIANamp Genomic DNA Kit (TIANGEN). Quantitative real-time PCR was performed for both whole-cell extracts and cytosolic fractions using nuclear DNA primers (*Tert*) and mtDNA primers (*Dloop1-3*, *Cytb*, *ND1* and *ND4*), and the Ct values obtained for mtDNA abundance for whole-cell extracts served as normalization controls for the mtDNA values determined from the cytosolic fractions. To determine the presence of mtRNA in the cytosol, qPCR was performed using primers specific for mitochondrial D-loop on cDNA, which had been reverse-transcribed from RNA isolated from the cytosolic fraction (mtRNA).

END-seq library preparation. END-seq was performed according to a previously described protocol(1). Briefly, L929 cells were treated as mentioned above. About 10 million cells were embedded in agarose plugs and subjected to protein and RNA digestion. The DNA within the plugs was blunted, A-tailed and ligated with a biotin-labelled hairpin adaptor. Subsequently, the agarose plugs were melted, and DNA was extracted and sheared by sonication. After C1 bead purification, the DNA fragments were ligated to a second adaptor and amplified for sequencing with Illumina sequencing primers.

Bioinformatics analysis. The genome-wide transcriptome data quantified as fragments per kilobase per million were downloaded from TCGA. We used the data from 12 cancer types, all of which had profiled enough numbers of samples for both cancer and normal tissues. Data include Bladder Urothelial Carcinoma (BLCA), Breast Cancer (BRCA), Colon Adenocarcinoma (COAD), Esophageal Carcinoma (ESCA), Head and Neck Squamous Cell Carcinoma (HNSC), kidney clear cell carcinoma (KIRC), lung adenocarcinoma (LUAD), Lung Squamous Cell Carcinoma (LUSC), Mesothelioma

(MESO), Ovarian Cancer (OV), rectum adenocarcinoma (READ) and Uterine Corpus Endometrial Carcinoma (UCEC). We identified and evaluated the levels of immune infiltrates of $CD8^+$ T cell by the Tumor immune estimation resource (TIMER) algorithm, which is a resource for systematic estimation of the abundances of six immune cell types (B cell, $CD4^+$ T cell, $CD8^+$ T cell, neutrophil, macrophage and dendritic cell) by using transcriptome profile(2). We characterized the overall activity of the aspartate/pyrimidine synthesis pathway by calculating the average expression of genes involved in this pathway in each tumor sample including GOT2, GPT2, CAD, DHODH, and UMPS. The Pearson correlation analysis was performed to determine the associations between aspartate/pyrimidine synthesis activity and $CD8^+$ T cell fraction.

References

1. Canela A, Sridharan S, Sciascia N, Tubbs A, Meltzer P, Sleckman BP, et al. DNA Breaks and End Resection Measured Genome-wide by End Sequencing. *Mol Cell.* 2016;63(5):898-911.
2. Li T, Fu J, Zeng Z, Cohen D, Li J, Chen Q, et al. TIMER2.0 for analysis of tumor-infiltrating immune cells. *Nucleic Acids Res.* 2020;48(W1):W509-W14.

Supplemental Figure Legends

Supplemental Figure 1. The aminotransferase inhibitor AOA induces and enhances ISGs in cells.

(**A**) MC38 cells were treated with inhibitors from (Figure 1A) followed by 0.5μg/ml HT-DNA transfection for 6h, cells were harvested for qPCR analysis of *Ifnb.* (**B**-**D**) RAW264.7 (mouse macrophage cell line), HT-1080 (human fibrosarcoma cell line), B16-F10 (mouse melanoma cell line) were treated with 0.5mM AOA and/or HT-DNA (0.5μg/ml) for 6 h, cells were harvested for qPCR analysis of ISGs expression. (**E-G**) Secreted IFN-B protein levels in culture supernatants were measured by ELISA. (**H-J**) Western blot detected P-IRF3, P-TBK1 levels in different cell line treated as indicated. (**K**) Western blot analysis in L929 cell monoclones transduced with the indicated gene-specific guide RNA (gRNA) or scrambled (Scr) gRNA control to determine the knockout efficiency. (**L**) cGAS-KO/STING-KO/IRF3-KO L929 cell lines were treated with 0.5 mM AOA and/or HT-DNA (0.5μg/ml) for 6 h, cells were harvested for qPCR analysis of ISGs expression. (**M**-**O**) *Rigi* or *Ifih1* knockdown MC38 cells were treated with AOA and/or HT-DNA for 6 h, followed by qPCR analysis of *Rigi* (**M**) or *Ifih1* (**N**) knockdown efficiency and ISGs expression (**O**). Data are represented as means ± SEM. Representative data are shown from two or three independent experiments. Statistical analysis was performed by unpaired *t* test (**B**-**G, O**) and 1-way ANOVA (**M, N**) or 2-way ANOVA (**L**) followed by Tukey's test. *$P < 0.05$; **$P < 0.01$; ***$P < 0.001$; ****$P < 0.0001$.

Supplemental Figure 2. Co-depletion of GOT2 and GPT2 slightly induced ISG expression and exhibited amplification of DNA-stimulated ISG expression.

(**A** and **B**) The relative *IFNB* and *CXCL10* mRNA expression in the control BJ-5ta cells versus *GOT1*

siRNA-silencing BJ-5ta cells (**A**) or *GOT2* siRNA-silencing BJ-5ta cells (**B**). (**C**) The relative *GPT2* mRNA expression in the control BJ-5ta cells versus *GOT2* siRNA-silencing BJ-5ta cells. (**D**) The relative *IFNB* and *CXCL10* mRNA expression in the control BJ-5ta cells versus *GPT2* siRNA-silencing BJ-5ta cells. (**E**) The relative ISG expression in the control MC38 cells versus *Gpt2* knockdown MC38 cells after indicated treatment. (**F**) The relative ISG expression in the control MC38 cells versus *Got2* and *Gpt2* double knockdown MC38 cells after indicated treatment. (**G**) Western blot detected P-IRF3, P-STAT1, GOT2 and GPT2 levels in MC38 cells treated as in (**F**). (**H** and **I**) Relative *IFNB* mRNA levels (**H**) and effector protein expression (**I**) in control BJ-5ta cells and BJ-5ta cells with *GPT2/GOT2* double knockdown following the indicated treatments. Data are represented as means ± SEM. Representative data are shown from two or three independent experiments. Statistical analysis was performed by unpaired *t* test (**A**-**F**) or 2-way ANOVA followed by Tukey's test (**H**).

Supplemental Figure 3. Aspartate deficiency augments cGAS-STING-mediated type I interferon response.

(**A**) Relative aspartate and asparagine levels in L929 cells pre-treated with 0.5 mM AOA for 1 h followed by HT-DNA (0.5μg/ml) transfection for 6 h or not compared with Mock group (n=3). (**B**) Relative aspartate levels in L929 cells after indicated treatment compared with Mock (shScr) group. (**C-G**) BJ-5ta, HT-1080, RAW264.7, B16-F10 and MC38 cells were treated with 0.5 mM AOA for 1 h followed by DMXAA (50μM) stimulation or HT-DNA (0.5μg/ml) transfection or not for 6 h in the absence or presence of 20 mM aspartate, and then cells were harvested for qPCR analysis of IFN response gene expression. (**H**) KEGG pathways enrichment of the up-regulated genes. (**I-M**) Western blot detected P-IRF3, P-TBK1 levels in different cell lines treated as in **C**-**G**. Data are represented as

means ± SEM. Representative data are shown from two or three independent experiments. Statistical analysis was performed by unpaired *t* test (**A**) or 1-way ANOVA followed by Tukey's test (**B**-**G**). **P* < 0.05; ***P* < 0.01; ****P* < 0.001; *****P* < 0.0001.

Supplemental Figure 4. The effect of AOA on enhancing *ISG*s cannot be reversed by most of examined metabolites.

(**A**) Relative abundances of metabolites in TCA and urea cycle were measured in L929 cells treated as indicated by LC-MS. (**B-E**) BJ-5ta were treated with or without 1mM AOA for 1 h followed by HT-DNA (0.5μg/ml) stimulation for 6 h in the absence or presence of indicated metabolites, and then cells were harvested for qPCR analysis of type I IFN expression. (**F-J**) L929 cells were treated with or without 0.5 mM AOA for 1 h followed by HT-DNA (0.5μg/ml) stimulation for 6 h in the absence or presence of indicated metabolites, and then cells were harvested for qPCR analysis of *Cxcl10* expression. (**K**) A schematic representation showing AOA induced ROS production and imbalanced NAD+/NADH ratio. (**L**) Intracellular NAD+/NADH ratio were measured by NAD+/NADH detection kit. L929 cells were treated as indicated. (**M**) L929 were treated with 0.5 mM AOA for 1 h followed by HT-DNA (0.5μg/ml) transfection for 6 h in the absence or presence of NAC (N-acetylcysteine), and then cells were harvested for qPCR analysis of ISGs expression. Data are represented as means ± SEM. Representative data are shown from two or three independent experiments. Statistical analysis was performed by unpaired *t* test (**A**) or 1-way ANOVA followed by Tukey's or Bonferroni's test (**B**-**M**). **P* < 0.05; ***P* < 0.01; ****P* < 0.001; *****P* < 0.0001.

Supplemental Figure 5. The effect of AOA on enhancing *ISG* expression is hindered by

pyrimidine nucleosides.

(**A** and **B**) L929 (**A**) or MC38 (**B**) cells were treated with 0.5 mM AOA for 1 h followed by HT-DNA (0.5 μg/ml) stimulation for 6 h in the absence or presence of pyrimidine nucleosides (+100μM, ++200μM cytidine, thymidine and uridine), and then cells were harvested for qPCR analysis of ISGs expression. (**C**) Verifying *Cad* knockdown efficiency by qPCR in L929 cells. Data are represented as means ± SEM. Representative data are shown from two or three independent experiments. Statistical analysis was performed by 1-way ANOVA followed by Tukey's test (**A**-**C**). *P < 0.05; **P < 0.01; ***P < 0.001; ****P < 0.0001.

Supplemental Figure 6. The effect of AOA on enhancing ISG expression is not associated with DNA damage or cell apoptosis.

(**A** and **B**) Western blot detected γH2AX and P-IRF3 levels in L929 (**A**) and BJ-5ta cells (**B**) treated as indicated. (**C**) MC38 cells were treated with AOA for 6 h, followed by collection of whole-cell lysates (WCL) or isolation of mitochondrial (Mito) and nuclear (Nuc) fractions. Western blot validation of subcellular fractionation. (**D**-**F**) Mass spectrometry analysis of relative aspartate levels in WCL (**D**), mitochondrial (**E**), and nuclear fractions (**F**) (n=3). (**G**) Western blot analysis of BAX and BAK in L929 cells to determine the knockout efficiency. (**H**) *Bax* and *Bak* knockout L929 cells were treated with 0.5 mM AOA and/or HT-DNA (0.5μg/ml) for 6 h, cells were harvested for qPCR analysis ISGs expression. (**I** and **J**) Representative flow cytometric images (**I**) and quantitative analysis (**J**) of the percentage of FVS780$^+$Annexin V$^+$ cells in L929 cells treated with different dose of AOA. Representative data are shown from two independent experiments. Statistical analysis was performed by unpaired *t* test (**D**-**F**) or 1-way ANOVA followed by Tukey's test (**H** and **J**). *P < 0.05; **P < 0.01; ***P < 0.001; ****P <

0.0001.

Supplemental Figure 7. AOA triggers mtDNA release to amplify cGAS-STING activation.

(**A**) Schematic of the subcellular fractionation protocol. (**B**) Western blot detected α-Tubulin (cytosol), TOM20 (OMM), PDH (Matrix), COX IV (IMM) and Histone H3 (nuclei) to validate pure cytosolic fraction in MC38 cells treated as indicated. (**C**) MC38 cells were treated with AOA in the absence or presence of 20mM aspartate followed by HT-DNA transfection and then cells were harvested for qPCR analysis of mtDNA levels in cytosolic fractions. (**D** and **E**) MC38 cells (**D**) or L929 cells (**E**) were treated with AOA in the absence or presence of 20 mM aspartate and then cells were harvested for qPCR analysis of mtDNA levels in cytosol. (**F** and **G**) The relative cytosolic mtDNA levels in *Got2* and *Gpt2* shRNA-silencing and control MC38 cells (**F**) or siRNA-silencing and control L929 cells (**G**). (**H**) Relative mtDNA copy number of wild-type MC38 cells exposed to ddC for 7 days. (**I**) ISGs expression were analyzed by qPCR after indicated treatment. Representative data are shown from two or three independent experiments. (**J**) MC38 cells were treated AOA for 6 h, followed by qPCR analysis of mtRNA levels in cytosolic fractions. Statistical analysis was performed by unpaired *t* test (**F**-**H**, **J**) and 1-way ANOVA (**C**-**E**) or 2-way ANOVA (**I**) followed by Tukey's test. *$P < 0.05$; **$P < 0.01$; ***$P < 0.001$; ****$P < 0.0001$.

Supplemental Figure 8. Aspartate deficiency enhances cGAS-STING signaling through VDAC-mediated mtDNA release.

(**A**) Representative Immunofluorescence images of mitochondrial (TOM20, red) or dsDNA (green) in BJ-5ta cells treated with AOA in the absence or presence of VBIT-4 for 6 h. Scale bars, 10μm. Right:

quantification of cytosolic dsDNA in BJ-5ta cells with indicated treatment (n=48 fields per group from 3 biological replicates). (**B**) L929 cells were treated with 10μM VBIT-4 (inhibitor of voltage-dependent anion channel (VDAC) oligomerization) for 48 h and then treated with AOA and/or HT-DNA for 6 h. Cells were harvested for qPCR analysis of ISGs expression. (**C**) Scr and *Vdac1/3* knockdown L929 cells were treated as indicated, followed qPCR analysis of ISGs expression. (**D** and **E**) L929 cells (**D**) and MC38 cells (**E**) were treated as indicated, P-IRF3 and P-STAT1 levels were detected by western blot. (**F** and **G**) MC38 cells (**F**) and B16-F10 cells (**G**) were treated with 10μM VBIT-4 for 48 h and then treated with AOA and/or HT-DNA for 6 h. ISGs expression were analyzed by qPCR. Representative data are shown from two or three independent experiments. Statistical analysis was performed by 1-way ANOVA (**A**, **B**, **F**, **G**) or 2-way ANOVA (**C**) followed by Tukey's test. $*P < 0.05$; $**P < 0.01$; $***P < 0.001$; $****P < 0.0001$.

Supplemental Figure 9. The strengthening effect of mtDNA on the cGAS-STING pathway is independent of MLKL-mediated necroptotic signaling.

(**A** and **B**) qPCR analysis (**A**) and western blots (**B**) of Scr and *Zbp1*$^{-/-}$ L929 cells with indicated treatment. (**C**) L929 cells were treated with Necrostatin-1 (Nec-1, 10 mM) or GSK'872 (2.5 mM) for 48h, followed by HT-DNA stimulation for 6 hrs with or without AOA for qPCR analysis. (**D**) Endogenous coIP in L929 cells with indicated treatment. (**E** and **F**) qPCR analysis (**E**) and western blots (**F**) of Scr and *Mlkl*-knockdown L929 cells with indicated treatment. Data are represented as means ± SEM. Representative data are shown from two or three independent experiments. Statistical analysis was performed by 2-way ANOVA followed by Tukey's test (**A**, **C** and **E**). $*P < 0.05$; $**P < 0.01$; $***P < 0.001$; $****P < 0.0001$.

Supplemental Figure 10. AOA augments the effect of cGAMP on inducing type I IFN responses.

(**A-C**) Body weight of animals in Figure 6. (**D**) Gating strategies of FACS. (**E** and **F**) AOA enhances cGAMP-mediated antitumor response. Quantification analysis of the percentage of $CD8^+$ T cells in living cells and surface CD69 expression (**E**), IFNγ and TNFα production (**F**) on $CD8^+$ T cells isolated from tumors of B16-F10 tumor bearing mice. FACS analysis was performed on day 14 after inoculation with 2×10^5 tumor cells (n=7-9). Data are represented as means ± SEM. Representative data are shown from two or three independent experiments. Statistical analysis was performed by unpaired t test (**E** and **F**). *P < 0.05; **P < 0.01; ***P < 0.001; ****P < 0.0001.

Supplemental Figure 11. AOA does not directly alter $CD8^+$ T cell metabolism or function

(**A–C**) C57BL/6 mice were intraperitoneally injected with PBS or AOA (5 mg/kg) for 18 h, followed by FACS sorting of splenic $CD8^+$ T cells for metabolomic analysis. Schematic of the experimental design (**A**), heatmap of metabolite changes in $CD8^+$ T cells (n=3) (**B**) and relative aspartate levels in $CD8^+$ T cells (**C**). (**D–I**) $CD8^+$ T cells were sorted from spleens of C57BL/6 mice and treated in vitro with AOA (1 mM) for 1 h followed by cGAMP (5μM) stimulation for 6 h, then subjected to metabolomic or FACS analysis. Schematic of the experimental design (**D**), heatmap of metabolite changes in $CD8^+$ T cells(n=3) (**E**) and relative aspartate levels in $CD8^+$ T cells (**F**). FACS analysis of CD69 (**G**), IFNγ (**H**), and TNFα (**I**) expression in $CD8^+$ T cells. Data are represented as means ± SEM. Representative data are shown from two or three independent experiments. Statistical analysis was performed by unpaired t test (**C** and **F**, **G-I**). *P < 0.05; **P < 0.01; ***P < 0.001; ****P < 0.0001.

Supplemental Figure 12. AOA enhances $CD8^+$ T cell responses through type I interferon signaling

(**A–D**) $CD8^+$ T cells were sorted from spleens of C57BL/6 mice and cultured in MC38-derived conditioned medium for 24 h, followed by FACS analysis. Schematic of the experimental design (**A**). ELISA quantification of IFNB in MC38-derived conditioned medium (**B**). FACS analysis of CD69 expression (**C**) and IFNγ production (**D**) in $CD8^+$ T cells. (**E** and **F**) Tumor volume (**E**) and Kaplan–Meier survival curves (**F**) of C57BL/6 mice inoculated with 8×10^5 MC38 cells (n = 6). Mice received anti-CD8 antibody (10 mg/kg, i.p.) or isotype control on days 0, 3, 6, 9, and 12 and were treated with cGAMP (3μg/mouse) combined with AOA (5 mg/kg, i.p.) or PBS on days 7, 10, and 13. (**G** and **H**) Tumor volume (**G**) and Kaplan–Meier survival curves (**H**) of *Ifnar1*-KO C57BL/6 mice inoculated with 8×10^5 MC38 cells (n = 6). Mice were treated with cGAMP (3μg/mouse) combined with AOA (5 mg/kg, i.p.) or PBS on days 7, 10, and 13. (**I** and **J**) Tumor volume (**I**) and Kaplan–Meier survival curves (**J**) of C57BL/6 mice inoculated with 8×10^5 MC38 cells (n = 6). Mice received 20mg/kg of anti-IFNAR1 antibody or isotype control intraperitoneally on days 0, 3, 6, 9, and 12 and were treated with cGAMP (3μg/mouse) combined with AOA (5 mg/kg, i.p.) or PBS on days 7, 10, and 13. Data are represented as means ± SEM. Representative data are shown from two or three independent experiments. Statistical analysis was performed by 1-way ANOVA (**B-D**) and 2-way ANOVA followed by Tukey's test (**E**, **G**, **I**) or log-rank test (**F, H** and **J**). *P < 0.05; **P < 0.01; ***P < 0.001; ****P < 0.0001.

Supplemental Figure 13. AOA synergizes with chemotherapy-mediated antitumor immunity in the AOM/DSS CRC model

(**A**) Schematic of the AOM/DSS-induced CRC mouse model, treated with oxaliplatin or/and AOA. Oxaliplatin was administered every 4 days combined with daily injections of AOA for four injections. (**B**) Body weight dynamics of AOM/DSS-induced murine colorectal cancer model. (**C** and **D**) Representative colon images (**C**) and tumor number (**D**). Scale bars, 1 cm. (**E** and **F**) $CD8^+$ T cells producing IFNγ (**E**) and TNFα (**F**) in the lamina propria were analyzed on day 85 after AOM injection (n = 6 per group). Data are represented as means ± SEM. Representative data are shown from two or three independent experiments. Statistical analysis was performed by 1-way ANOVA followed by Bonferroni's test (**D-F**). *P < 0.05; **P < 0.01; ***P < 0.001; ****P < 0.0001.

Supplemental Figure 14. AOA synergizes with radiotherapy to enhance ISG induction and suppress tumor growth

(**A**) MC38 cells were irradiated with 6 Gy and treated with AOA for 24 or 48 h, followed by qPCR analysis of *Ifnb* and ISGs. (**B** and **C**) Tumor volume (**B**) and Kaplan–Meier survival curves (**C**) of C57BL/6 mice inoculated with 8×10^5 MC38 cells (n = 6). Mice received 6 Gy irradiation on days 11, 14, and 17 and daily AOA (5 mg/kg, i.p.) or PBS from days 11 to 19. (**D**) Body weight of mice in (**B**). Data are represented as means ± SEM. Representative data are shown from two or three independent experiments. Statistical analysis was performed by unpaired *t* test (**B** and **D**) or 1-way ANOVA followed by Tukey's test (**A**) and log-rank test (**C**). *P < 0.05; **P < 0.01; ***P < 0.001; ****P < 0.0001.

Supplemental Figure 15. Lower aspartate correlates with enhanced anti-tumor immunity in cancer patients

(**A**) Correlation analysis for aspartate-related pyrimidine synthesis pathway activity score level versus cGAS, STING1, IRF3 and ISG15 in human KIRC, KIRP and LIHC from TCGA Pan Cancer Atlas. R and P represent Pearson's correlation coefficients and 2-tailed P values. Abbreviation: KIRC (n = 524), Kidney renal clear cell carcinoma; KIRP (n = 284), Kidney renal papillary cell carcinoma; LIHC (n = 363), Liver hepatocellular carcinoma. (**B**) The correlation analysis of aspartate-related pyrimidine synthesis pathway activity score with $CD8^+$ T cell infiltration for TCGA cancer type. Each red line represents a regression line with Spearman's correlation coefficient (R). Abbreviation: BLCA (n = 404), Bladder Urothelial Carcinoma; BRCA (n = 1058), Breast invasive carcinoma; COAD (n = 435), Colon adenocarcinoma; ESCA (n = 152), Esophageal carcinoma; HNSC (n = 494), Head and Neck squamous cell carcinoma; KIRC (n = 524), Kidney renal clear cell carcinoma; LUAD (n = 497), Lung adenocarcinoma; LUSC (n = 489), Lung squamous cell carcinoma; MESO (n = 80), Mesothelioma; OV (n = 353), Ovarian serous cystadenocarcinoma; READ (n = 156), Rectum adenocarcinoma; UCEC (n = 536), Uterine Corpus Endometrial Carcinoma; (**C**) LC-MS analysis of aspartate in human colorectal tumor tissue (CRC, n = 20 patients). (**D**) Representative IHC and H&E staining of tumor tissue in human colorectal carcinomas, showing lymphocyte cell infiltration and pathology. Scale bars, 200μm. (**E**) The correlations of aspartate levels with CD3, CD8 or granzyme B expression in colorectal tumor tissue (CRC, n = 20 patients). (**F**) Diagram showing that intracellular aspartate deficiency blocks the CAD-mediated *de novo* pyrimidine synthesis pathway, resulting in the release of mitochondrial DNA (mtDNA), which amplifies cGAS-STING pathway response to agonists via the ZBP1-RIPK1/3 axis.

Table S1: List of primers used in qPCR

Gene	Forward Sequence	Reverse Sequence
Mouse-Actb	ATGCTCCCCGGGCTGTAT	CATAGGAGTCCTTCTGACCCATTC
Mouse-Ifnb	TCCGAGCAGAGATCTTCAGGAA	TGCAACCACCACTCATTCTGAG
Mouse-Cxcl10	GCCGTCATTTTCTGCCTCA	CGTCCTTGCGAGAGGGATC
Mouse-Ccl5	ACGTCAAGGAGTATTTCTACAC	GATGTATTCTTGAACCCACT
Mouse-Isg15	CATCCTGGTGAGGAACGAAAGG	CTCAGCCAGAACTGGTCTTCGT
Mouse-Ifi44	CTGATTACAAAAGAAGACATGACAGAC	AGGCAAAACCAAAGACTCCA
Mouse-Usp18	GAGAGGACCATGAAGAGGA	TAAACCAACCAGACCATGAG
Mouse-Ifit1	TACAGGCTGGAGTGTGCTGAGA	CTCCACTTTCAGAGCCTTCGCA
Mouse-Ifit3	GCTCAGGCTTACGTTGACAAGG	CTTTAGGCGTGTCCATCCTTCC
Mouse-mtDNA Dloop1	AATCTACCATCCTCCGTGAAACC	TCAGTTTAGCTACCCCCAAGTTTAA
Mouse-mtDNA Dloop2	CCCTTCCCATTTGGTCT	TGGTTTCACGGAGGATGG
Mouse-mtDNA Dloop3	TCCTCCGTGAAACCAACAA	AGCGAGAAGAGGGGCATT
Mouse-mtDNA Cytb	GCTTTCCACTTCATCTTACCATTTA	TGTTGGGTTGTTTGATCCTG
Mouse-mtDNA ND1	CTAGCAGAAACAAACCGGGC	CCGGCTGCGTATTCTACGTT
Mouse-mtDNA ND4	AACGGATCCACAGCCGTA	AGTCCTCGGGCCATGATT
Mouse-nucDNA Tert	CTAGCTCATGTGTCAAGACCCTCTT	GCCAGCACGTTTCTCTCGTT
Mouse-Cad	CTGCCCGGATTGATTGATGTC	GGTATTAGGCATAGCACAAACCA
Mouse-Vdac1	CCCACATACGCCGATCTTGG	GTGGTTTCCGTGTTGGCAGA
Mouse-Vdac3	CAAAGGGTATGGGTTTGGCAT	TTGGTCTCTAGGTTGCCTGAT
Mouse-Got2	GGACCTCCAGATCCCATCCT	GGTTTTCCGTTATCATCCCGGTA
Mouse-Gpt2	AACCATTCACTGAGGTAATCCGA	GGGCTGTTTAGTAGGTTTGGGTA
Human-ACTB	TCCCTGGAGAAGAGCTACG	GTAGTTTCGTGGATGCCACA
Human-IFNB	AGGACAGGATGAACTTTGAC	TGATAGACATTAGCCAGGAG
Human-CXCL10	TGGCATTCAAGGAGTACCTC	TTGTAGCAATGATCTCAACACG
Human-CCL5	GCTGTCATCCTCATTGCTACTG	TGGTGTAGAAATACTCCTTGATGTG
Human-ISG54	CTGAACCGAGCCCTGCCGAAC	GCTGCCTCGTTTTGCCCTTTGAG

Table S2: sgRNA and shRNA oligos used in this paper

Gene	Sequence	Comments
Cad-1	CCGAGTCTTGTCTGAACCTAA	shRNA
Cad-2	GCCTCCACGAAAGGTTCTAAT	shRNA
Vdac1	GAGTTGATAAATACCACGTTA	shRNA
Ripk1	CCTGAATGACATCAATGCAAA	shRNA
Ripk3	GCTATGGTTAATCTTCGTAAT	shRNA
Mlkl-1	GCAGGATTTGAGTTAAGCAAA	shRNA
Mlkl-2	CGGACAGCAAAGAGCACTAAA	shRNA
Rigi-1	CCACAGTTGATCCAAATGATA	shRNA
Rigi-2	ACTGGAACAGGTCGTTTATAA	shRNA
Ifih1-1	CCACAGAATCAGACACAAGTT	shRNA
Ifih1-2	CTACAAATCAACGACACGAT	shRNA
Vdac3	CGAGAGGATTGAAACGTCAAT	shRNA
Cgas	ATATTCTTGTAGCTCAATCC	sgRNA
Sting	GAAGGCCAAACATCCAACTG	sgRNA
Irf3	CCAGTGGTGCCTACACCCCG	sgRNA
Bax	GTTTCATCCAGGATCGAGCA	sgRNA
Bak	GGAACTCTGTGTCGTAGCGC	sgRNA
Zbp1-1	GAAGATCTACCACTCACGTC	sgRNA
Zbp1-2	AGTCCTTTACCGCCTGAAGA	sgRNA

Supplemental Figure 1

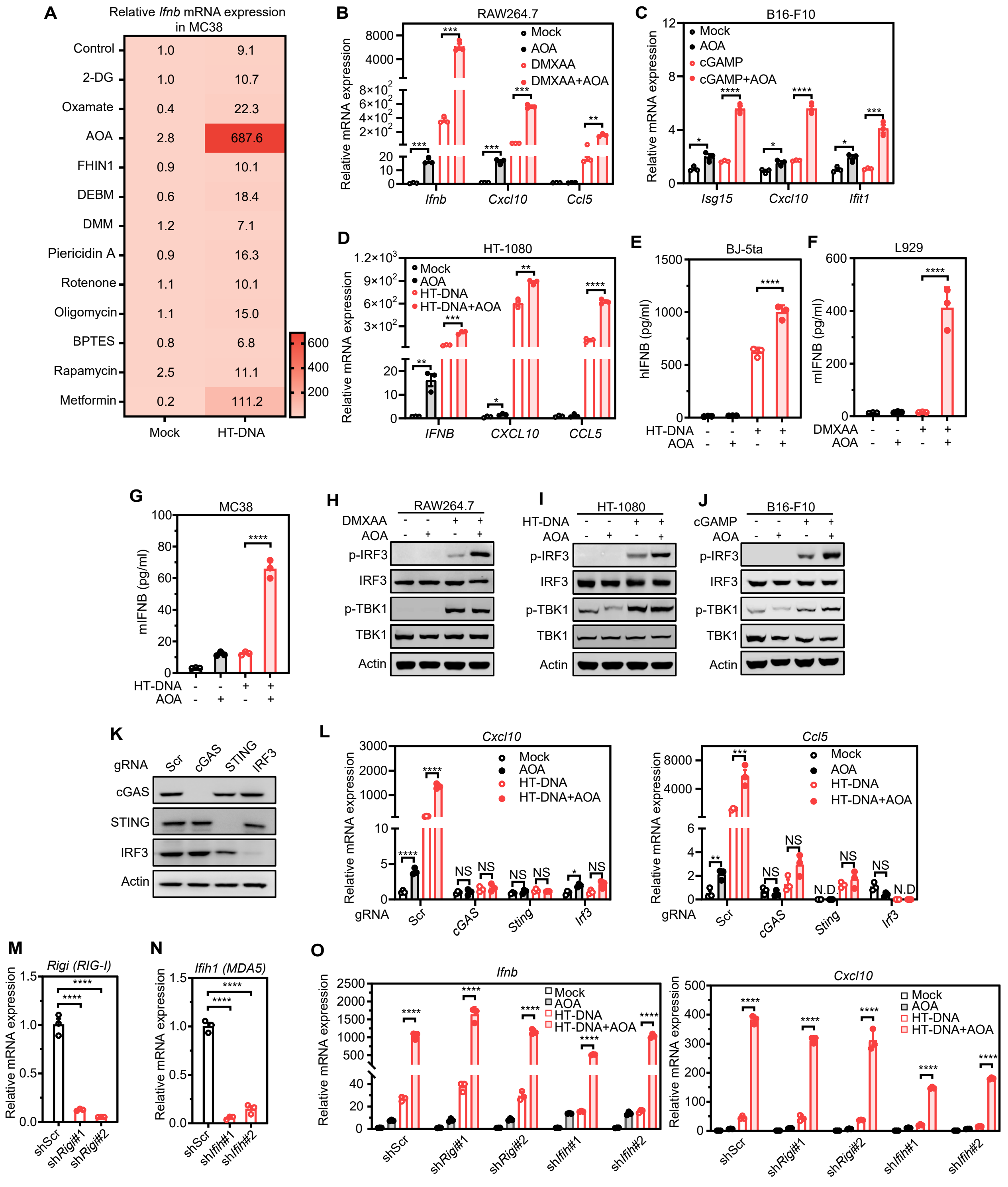

Supplemental Figure 2

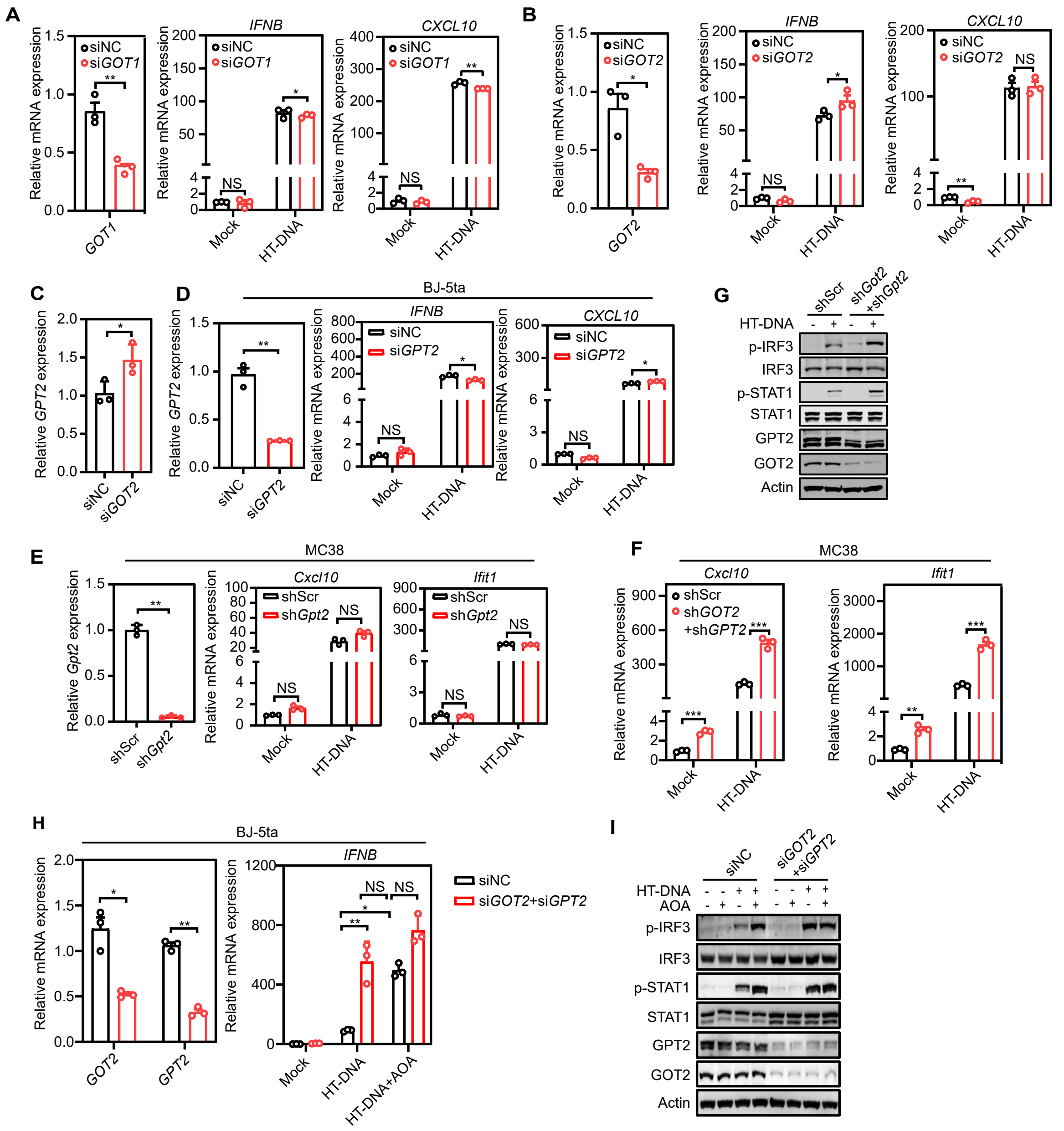

Supplemental Figure 3

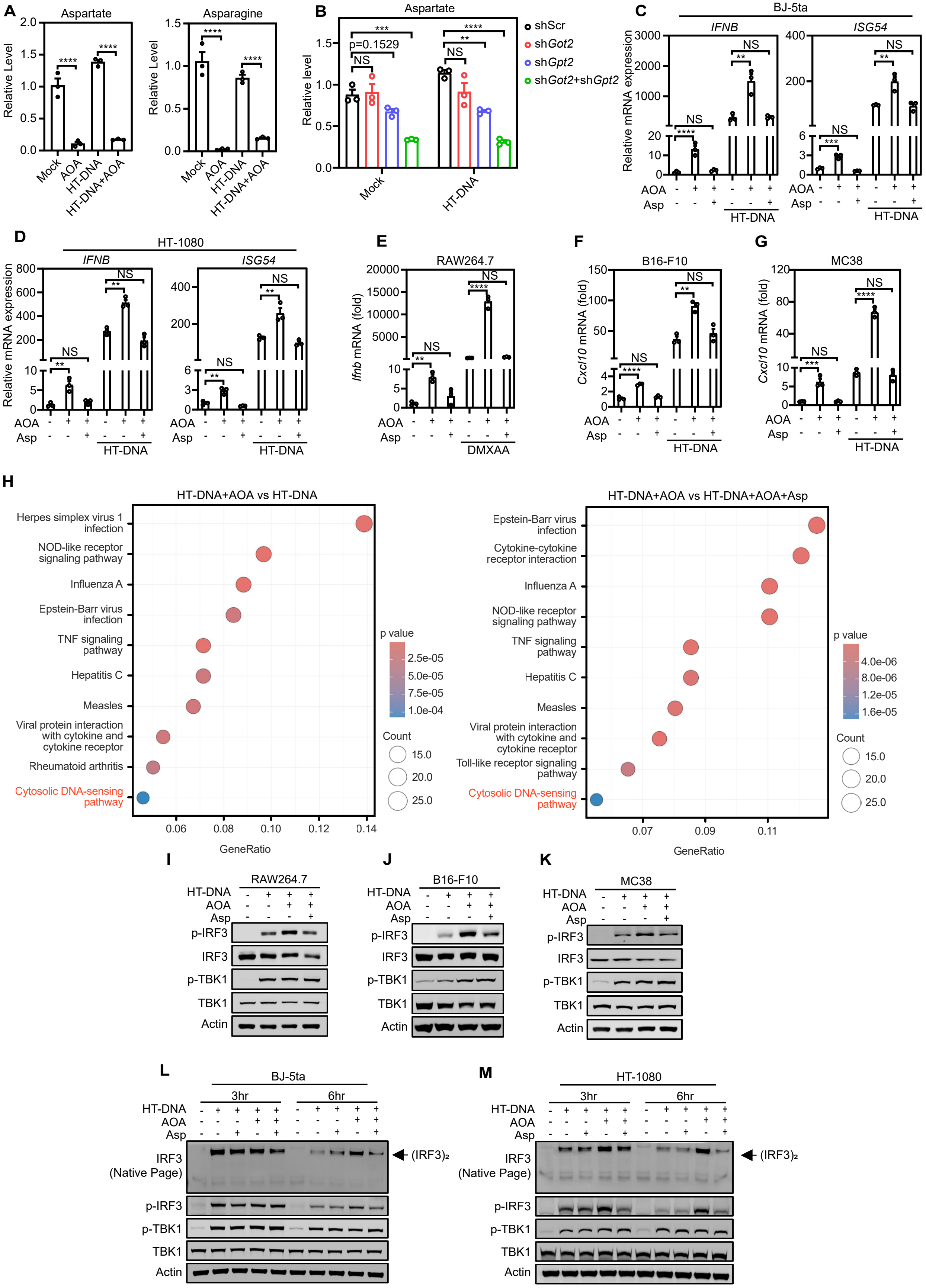

Supplemental Figure 4

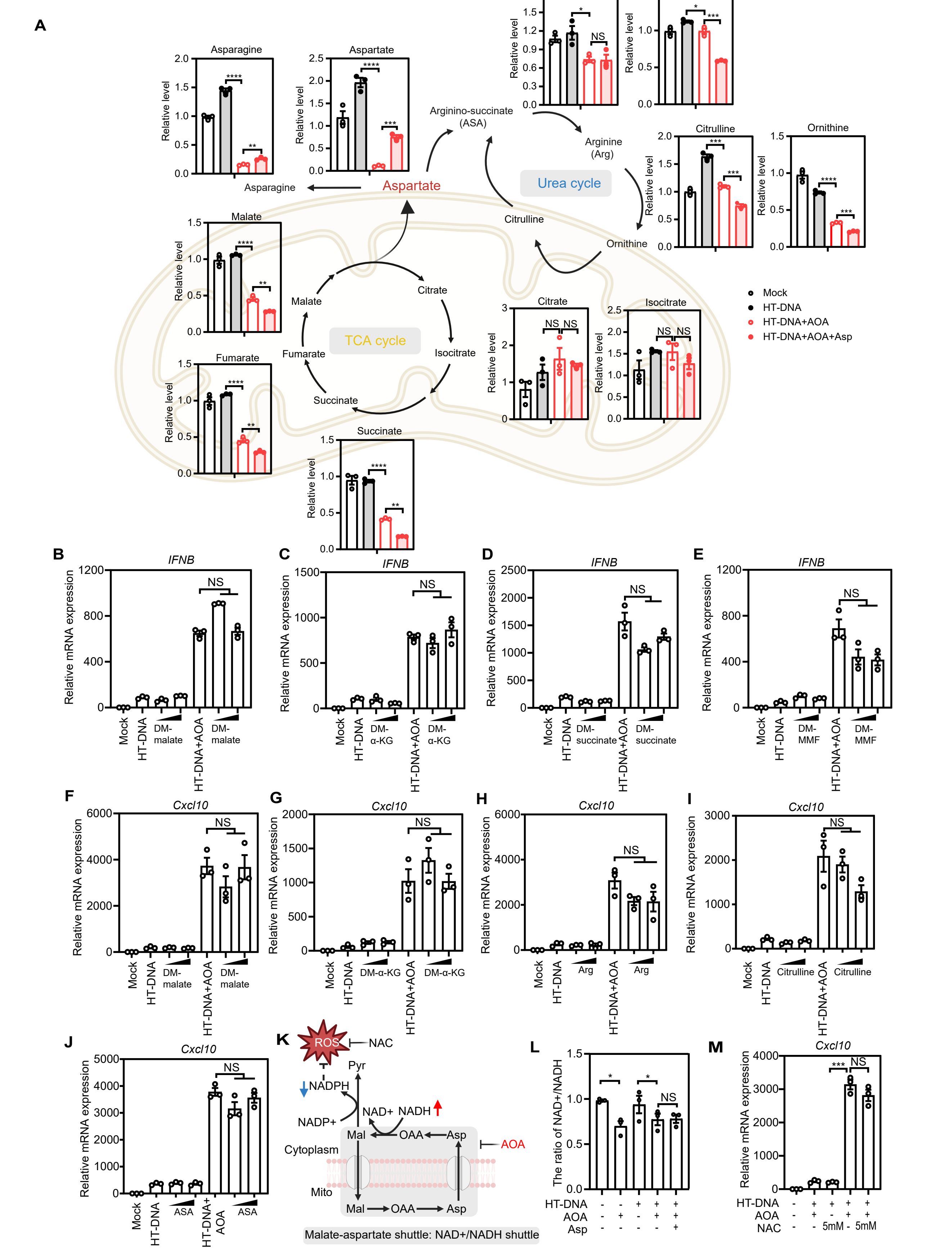

Supplemental Figure 5

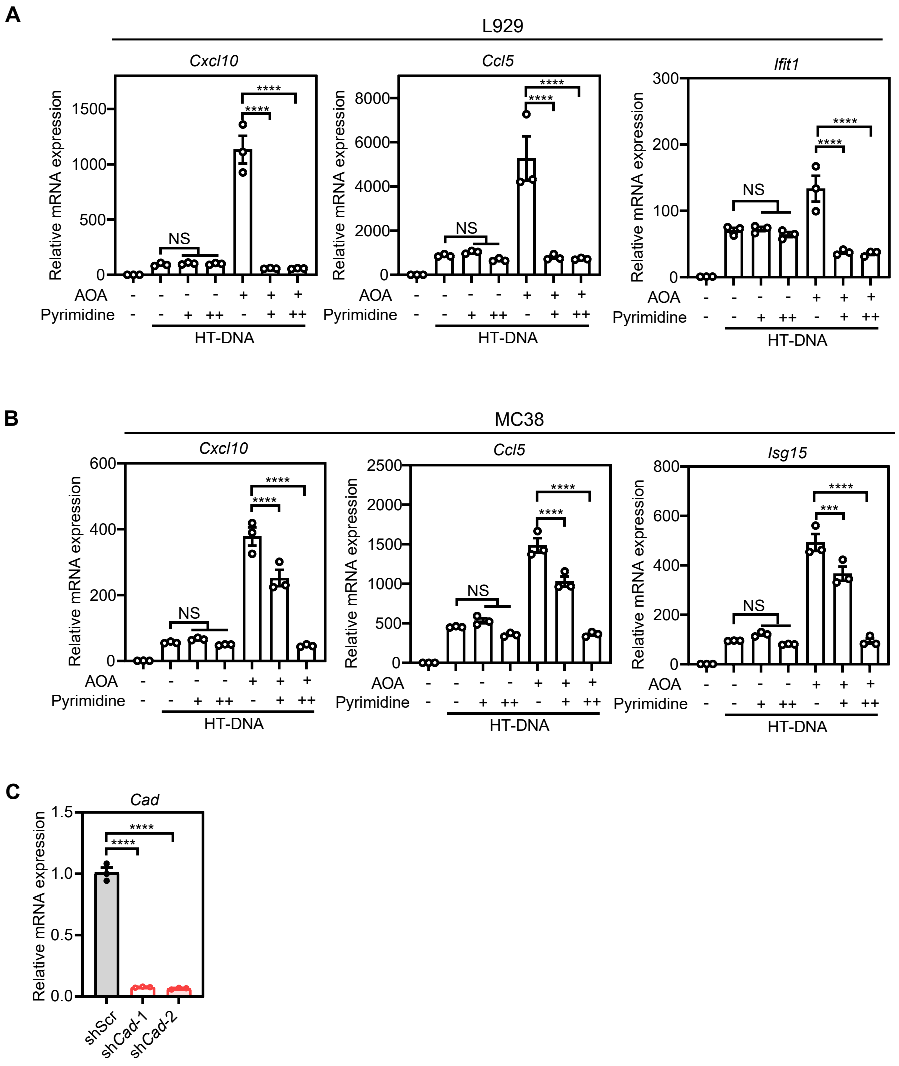

Supplemental Figure 6

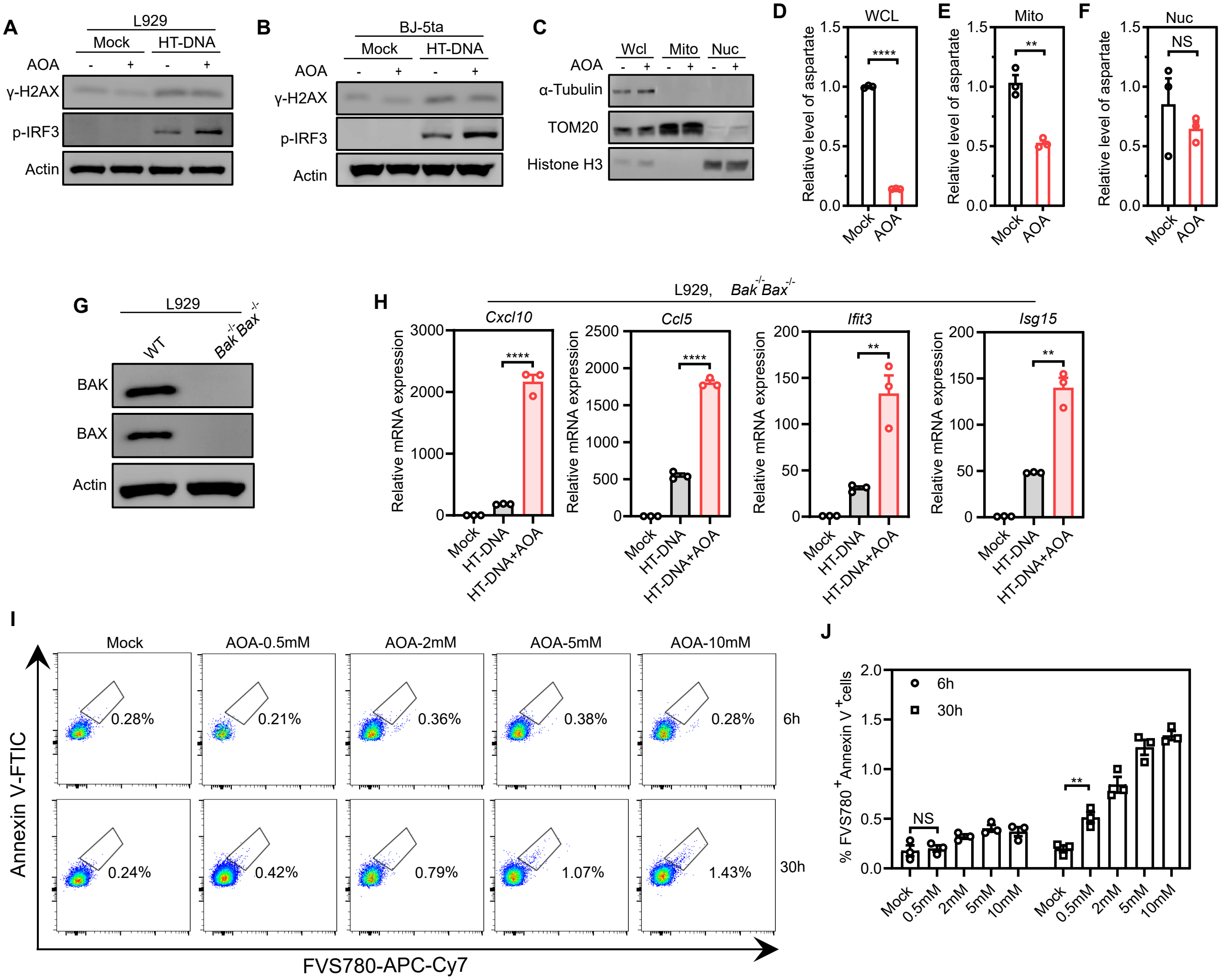

Supplemental Figure 7

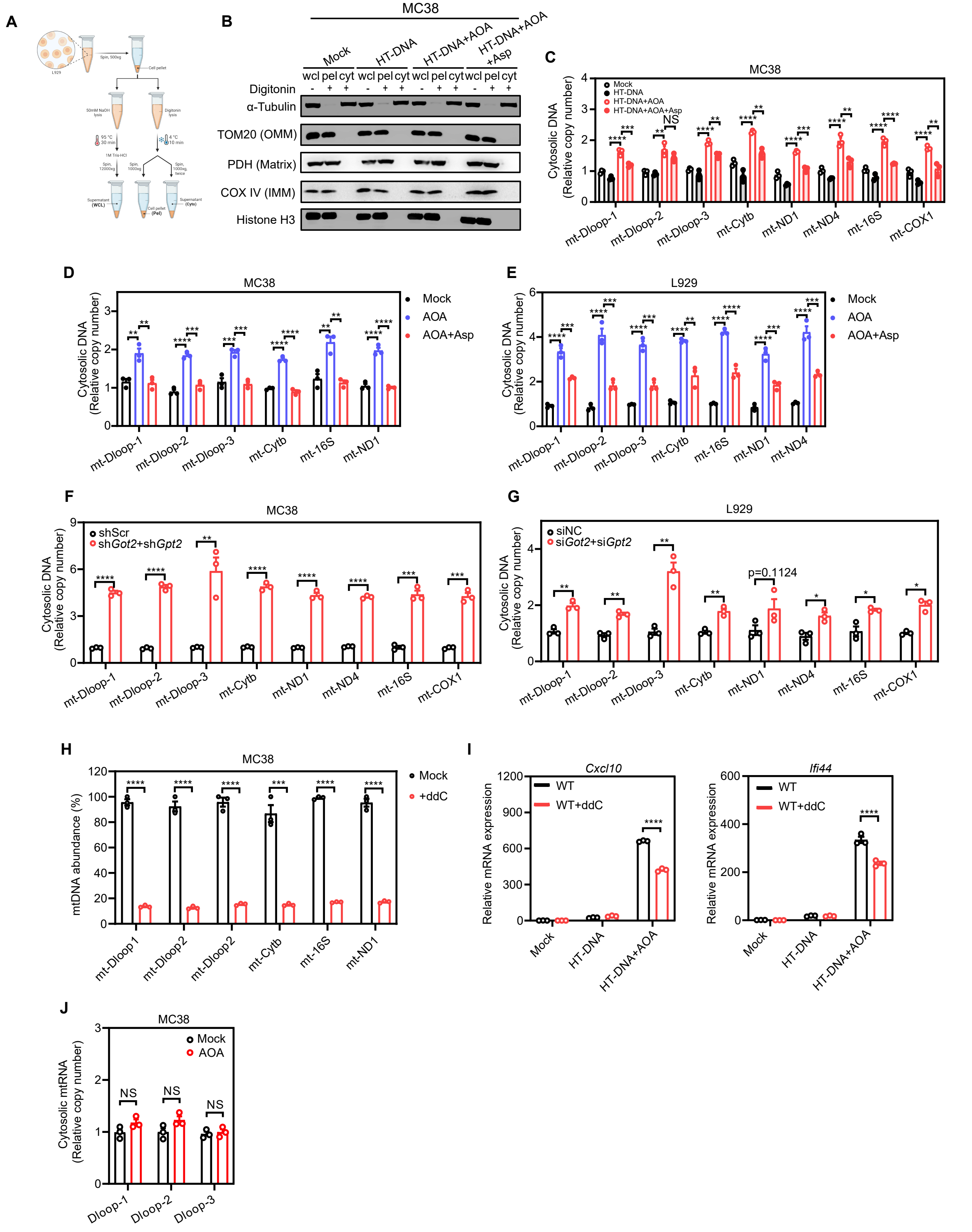

Supplemental Figure 8

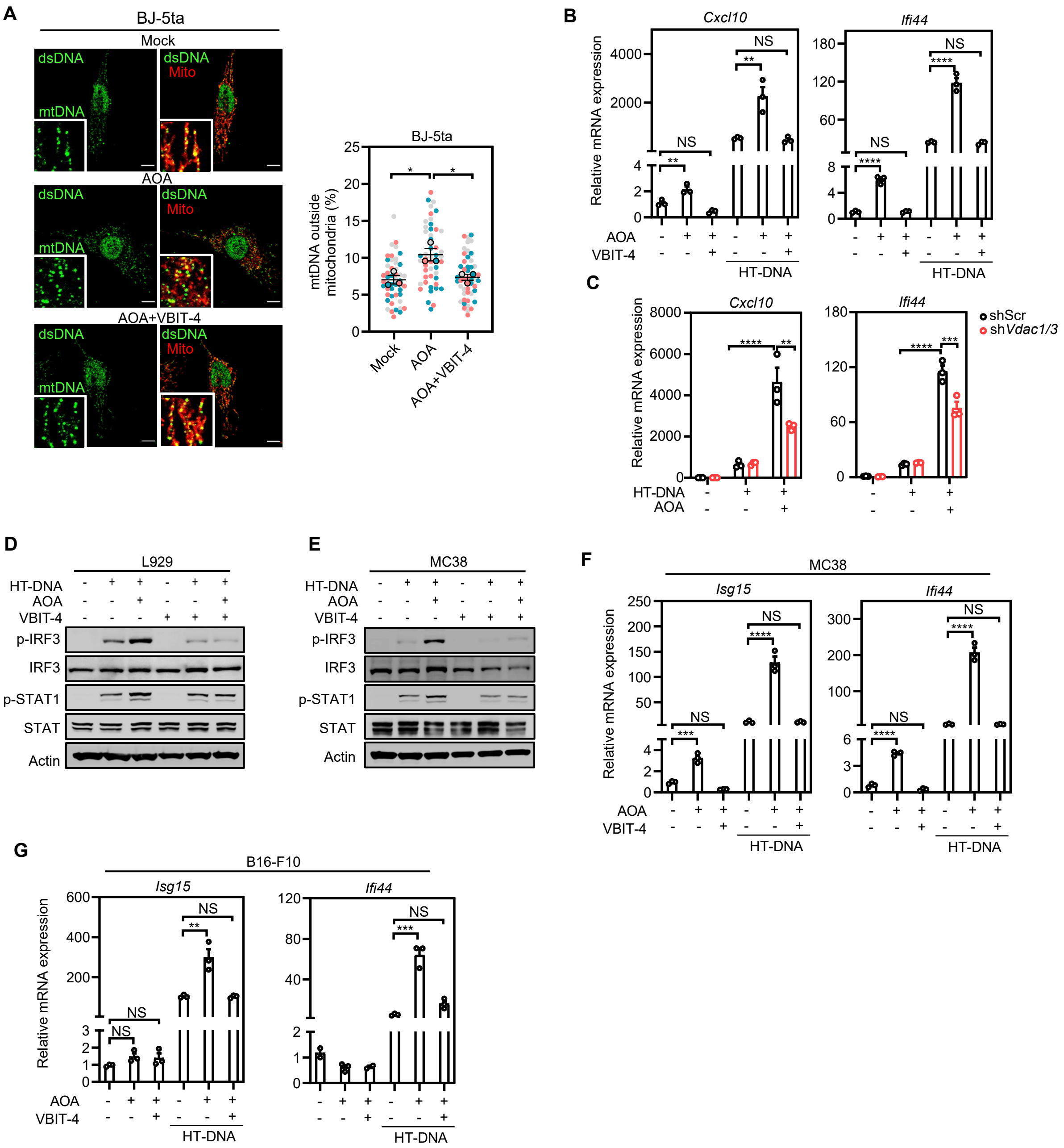

Supplemental Figure 9

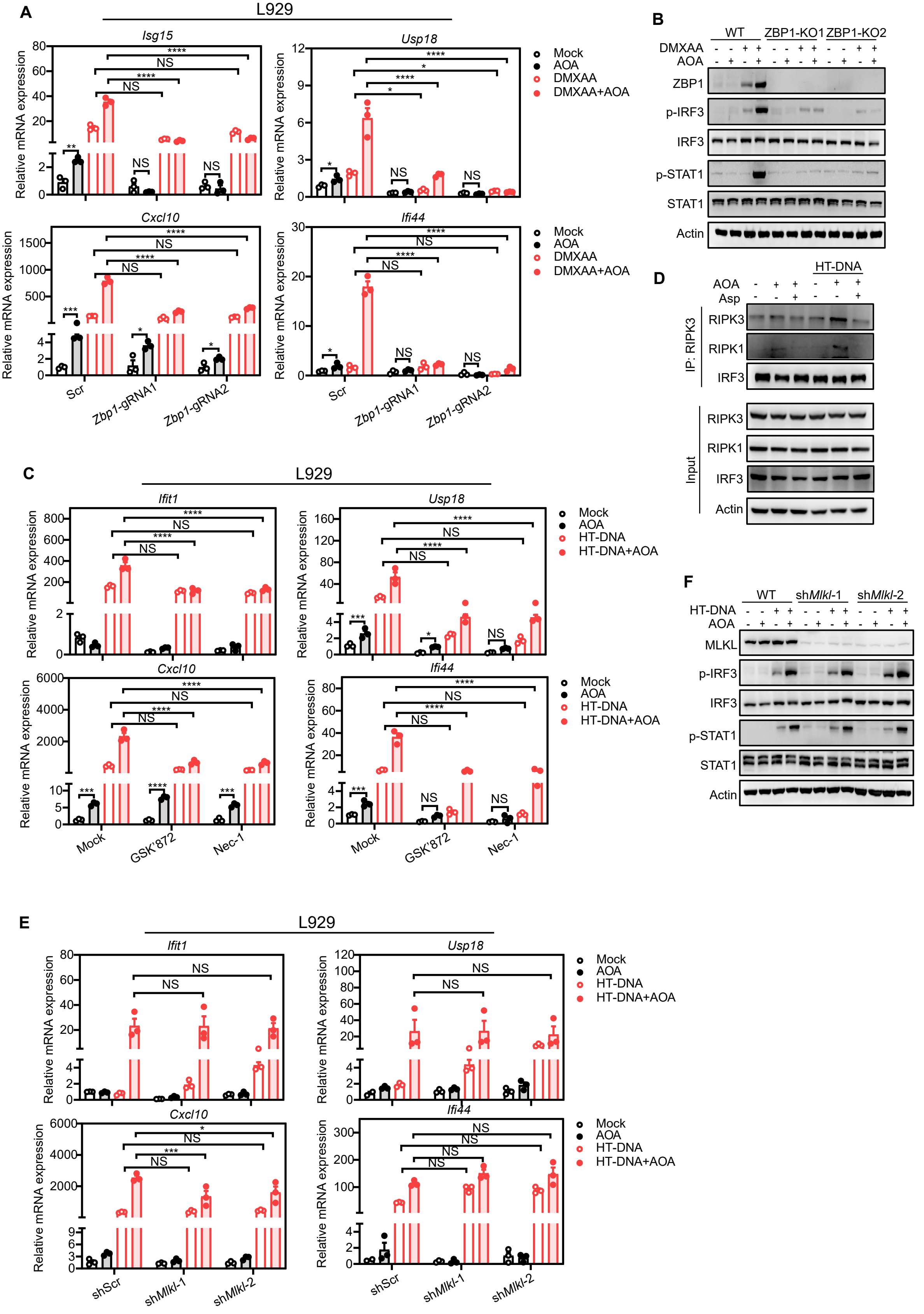

Supplemental Figure 10

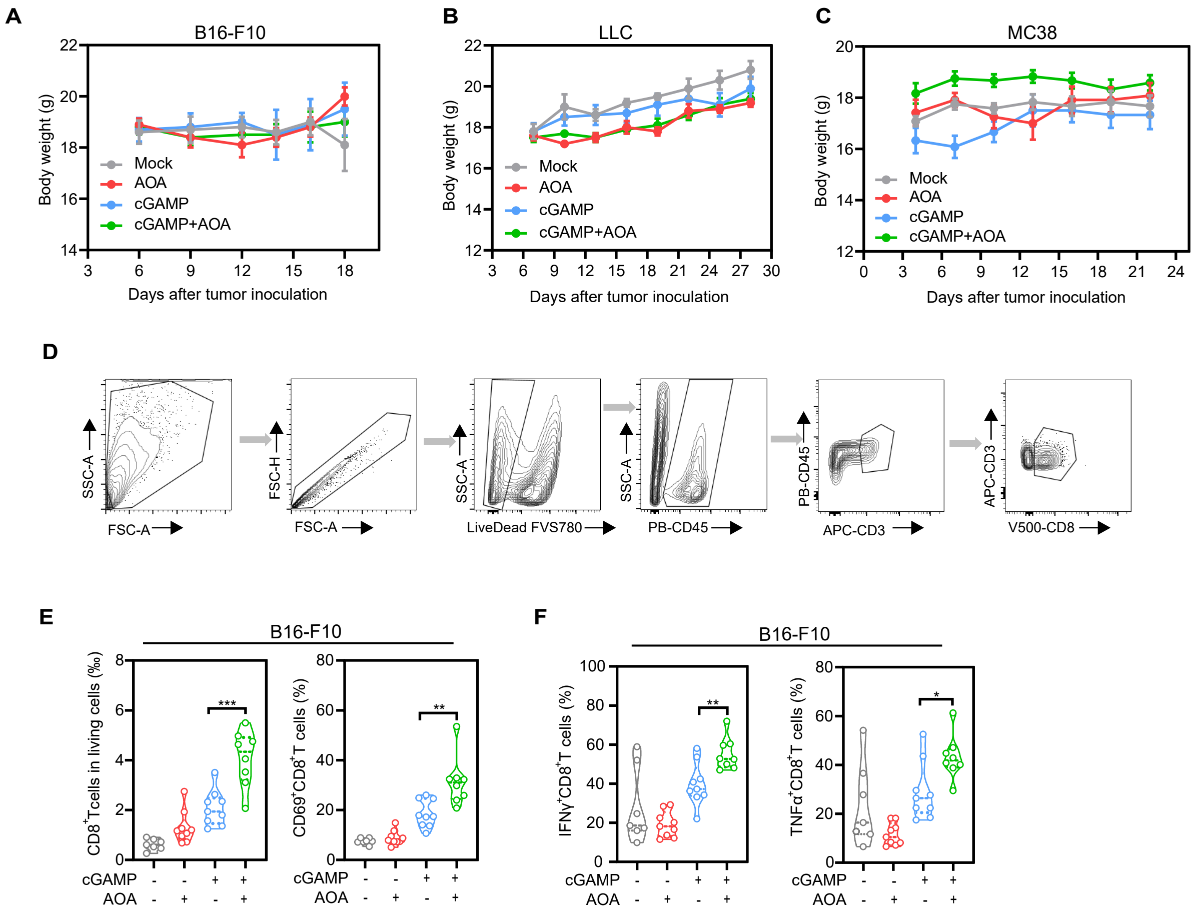

Supplemental Figure 11

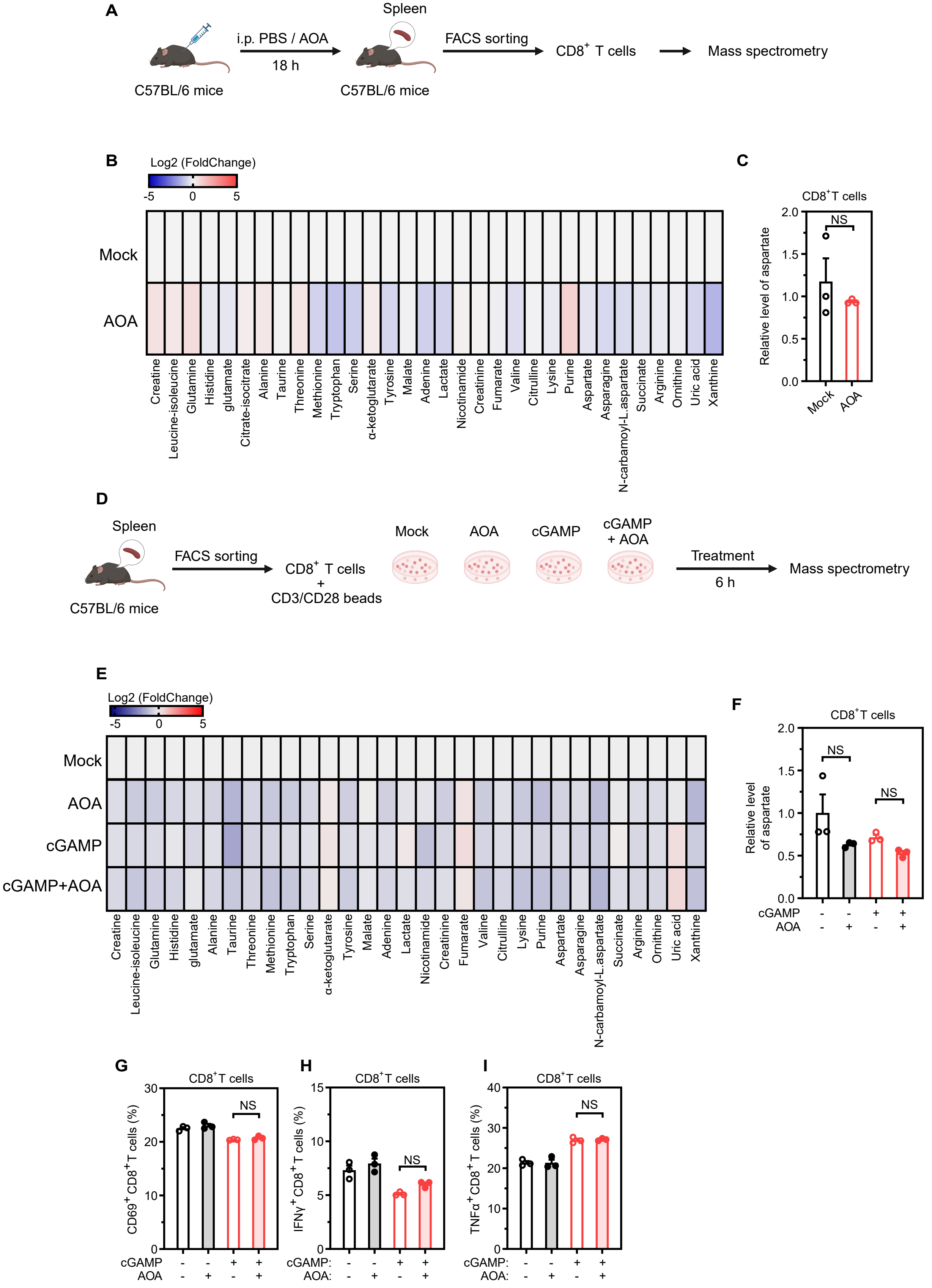

Supplemental Figure 12

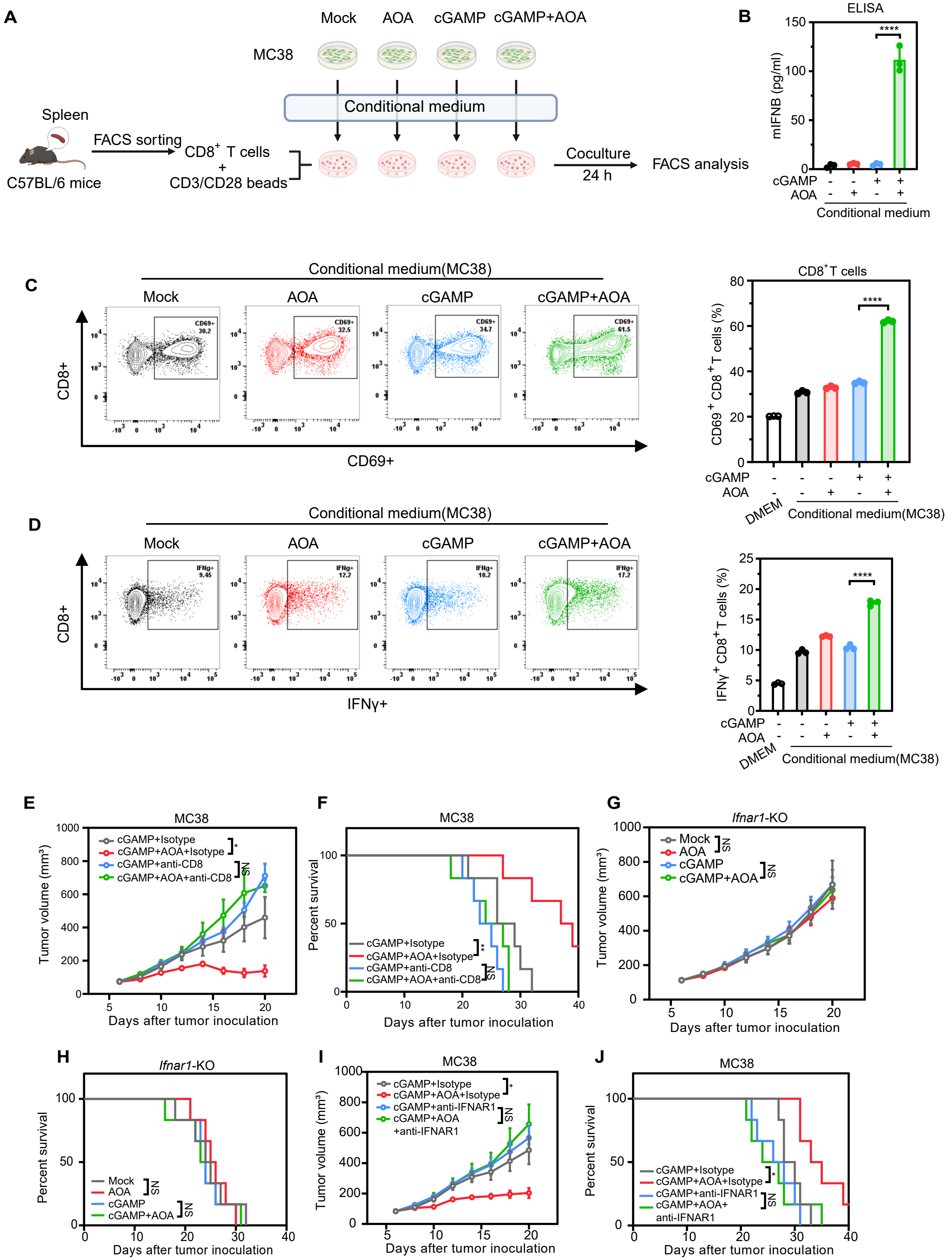

Supplemental Figure 13

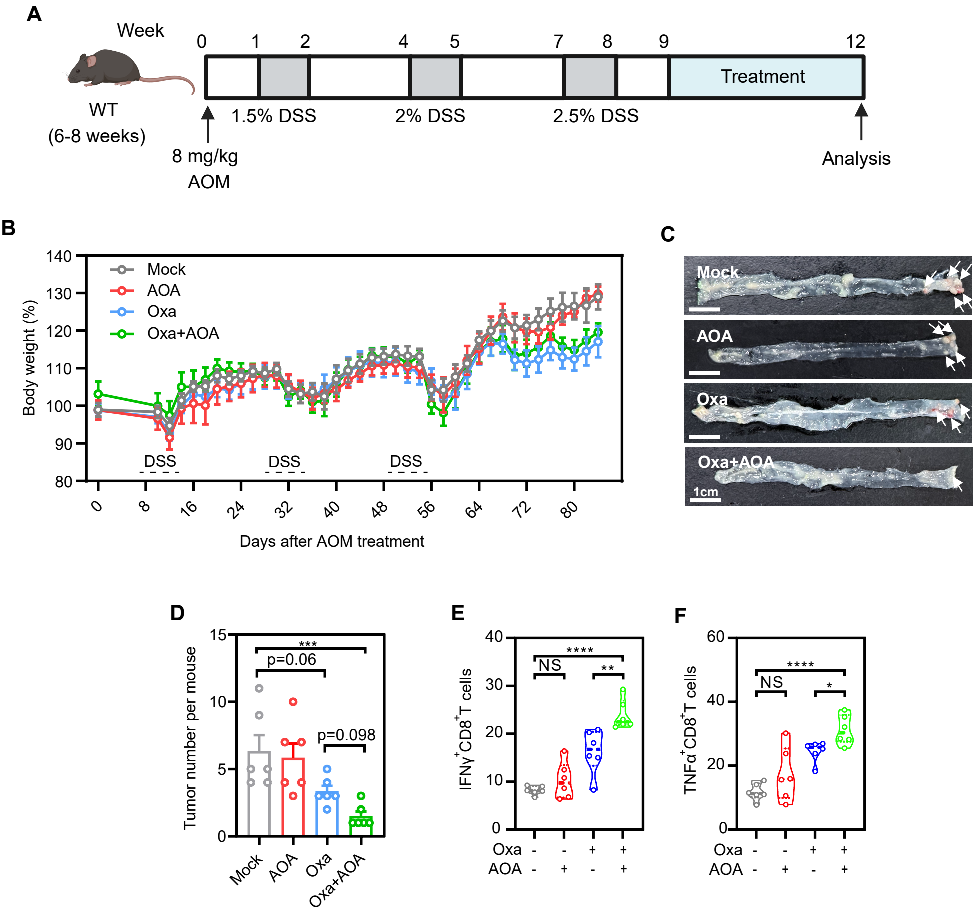

Supplemental Figure 14

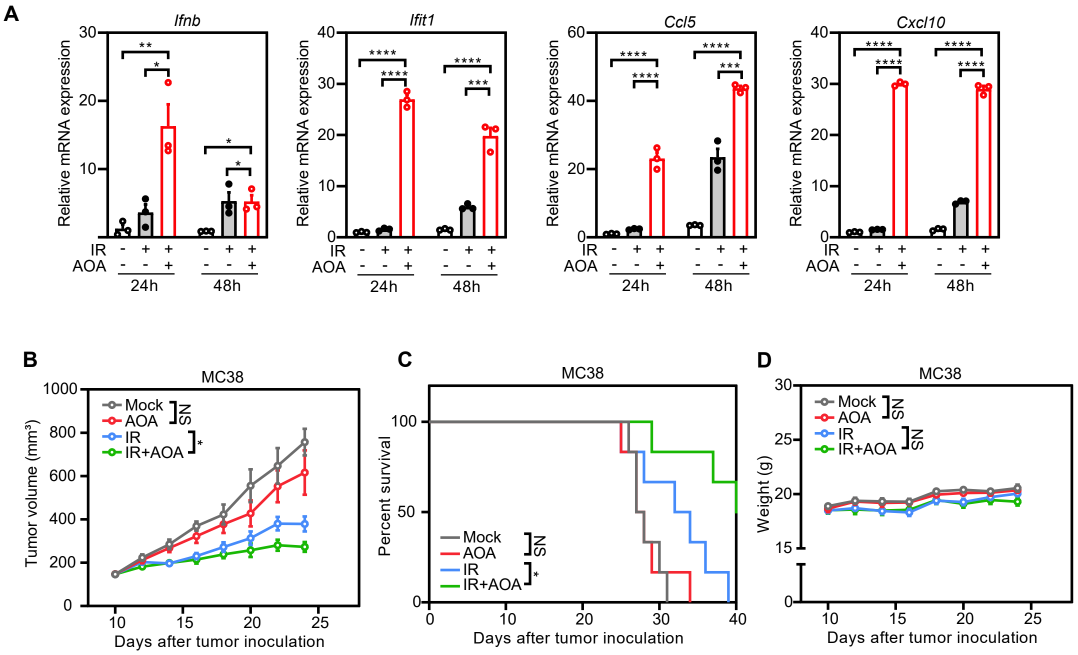

Supplemental Figure 15

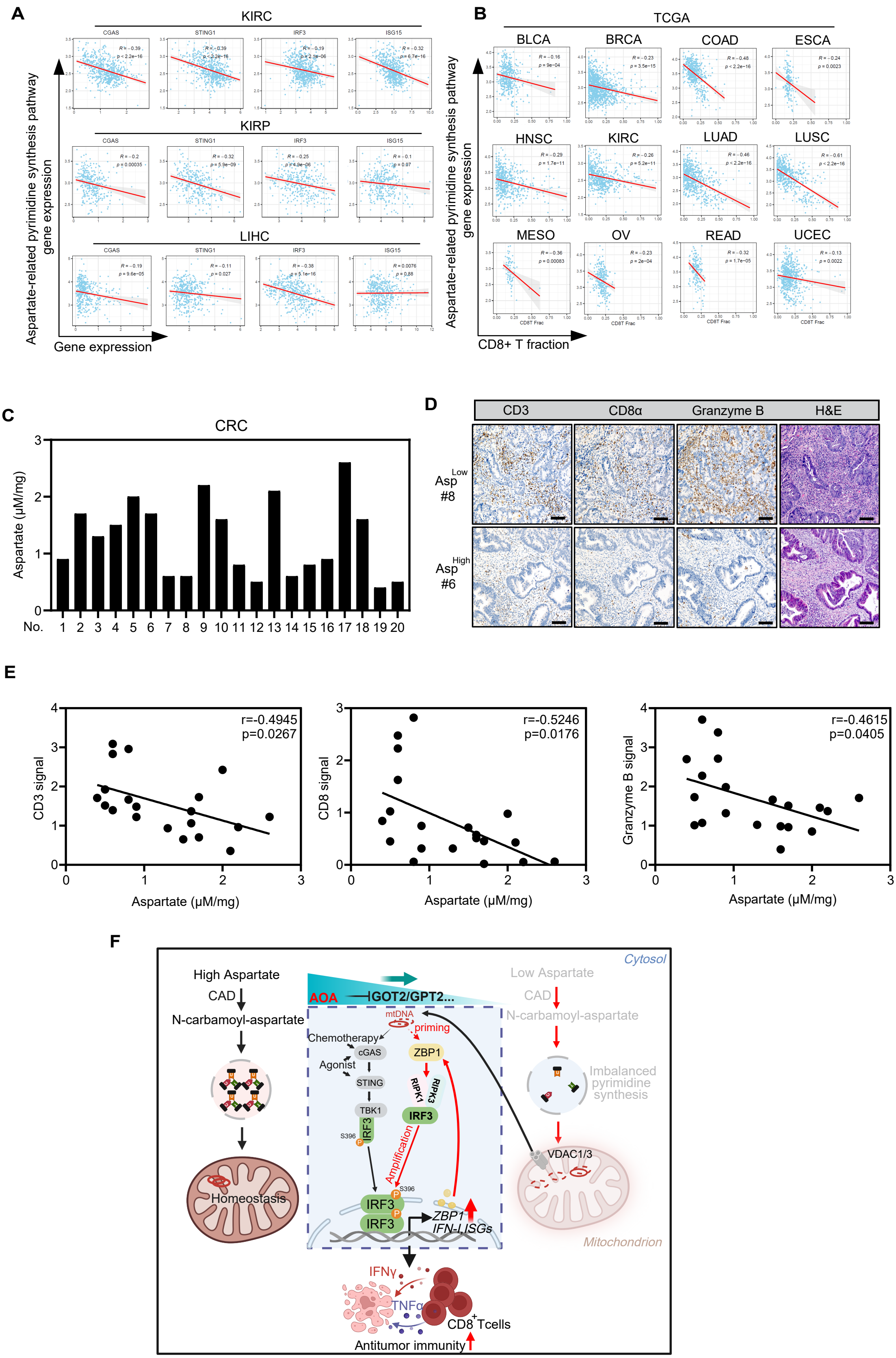

Supplement: Supplemental data [file jci-136-199716-s020.pdf]
