# Supplementary material for: Aspartate deficiency amplifies cGAS-STING signaling in antitumor immunity
Source: J Clin Invest. 2026 Jun 1;136(11):e199716. doi: 10.1172/JCI199716 (PMC13221234; doi:10.1172/JCI199716)

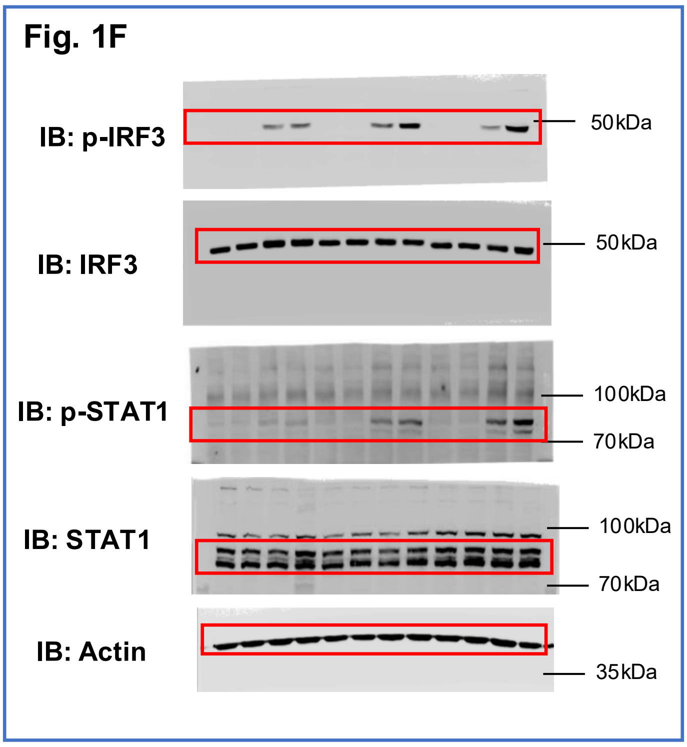

Fig. 1F
IB: p-IRF3
50kDa
IB: IRF3
50kDa
IB: p-STAT1
100kDa
70kDa
IB: STAT1
100kDa
70kDa
IB: Actin
35kDa

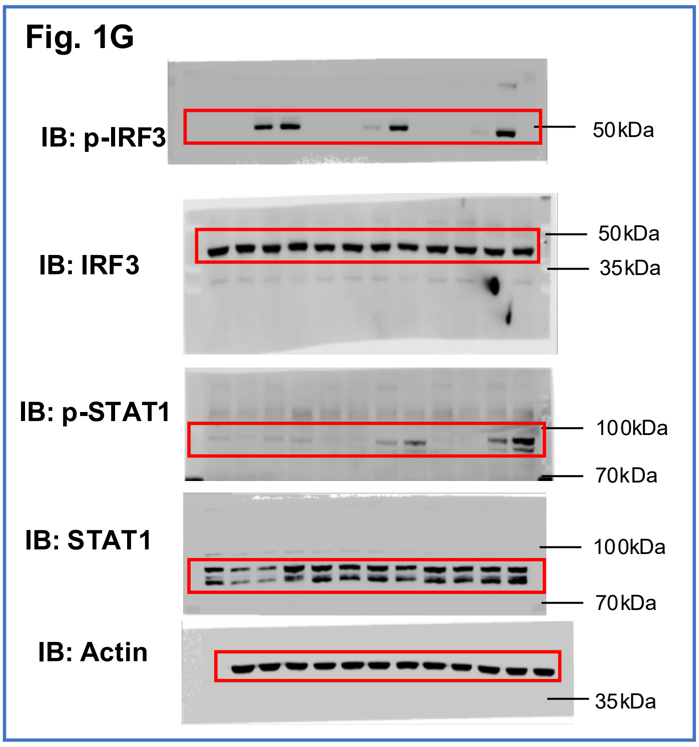

Fig. 1G
IB: p-IRF3
50kDa
IB: IRF3
50kDa
35kDa
IB: p-STAT1
100kDa
70kDa
IB: STAT1
100kDa
70kDa
IB: Actin
35kDa

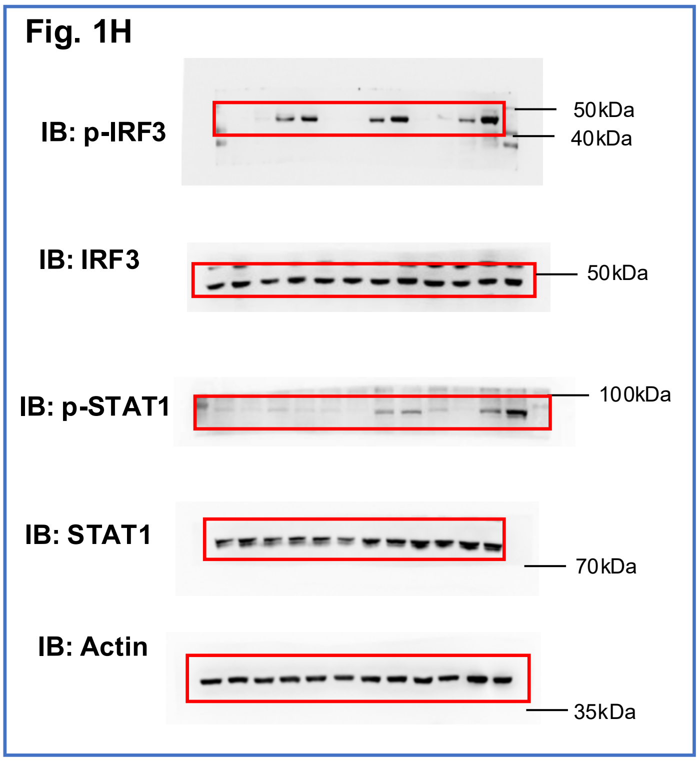

Fig. 1H
IB: p-IRF3
50kDa
40kDa
IB: IRF3
50kDa
IB: p-STAT1
100kDa
IB: STAT1
70kDa
IB: Actin
35kDa

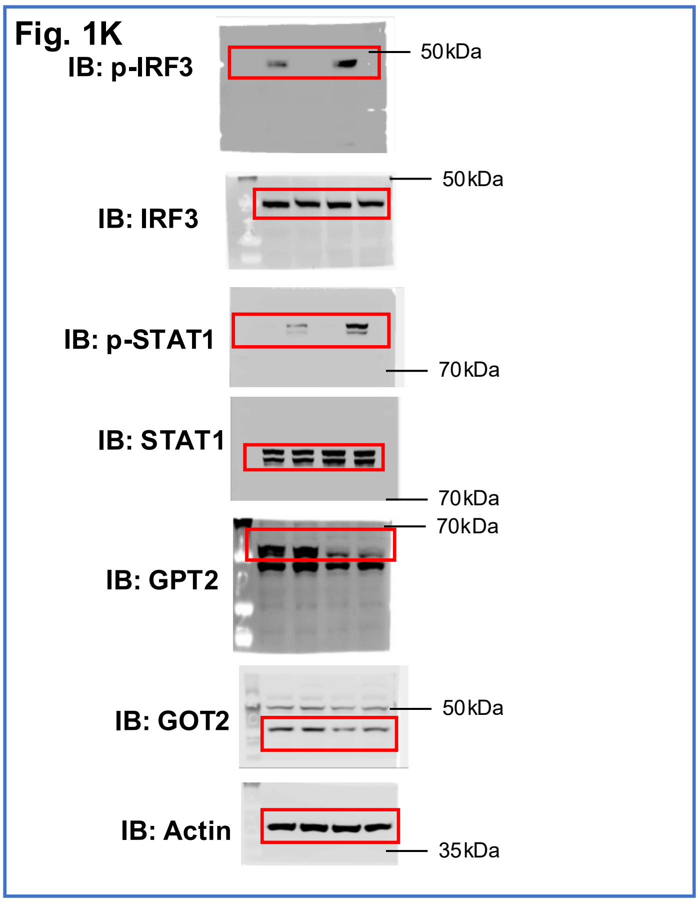

Fig. 1K
IB: p-IRF3
50kDa
IB: IRF3
50kDa
IB: p-STAT1
70kDa
IB: STAT1
70kDa
70kDa
IB: GPT2
IB: GOT2
50kDa
IB: Actin
35kDa

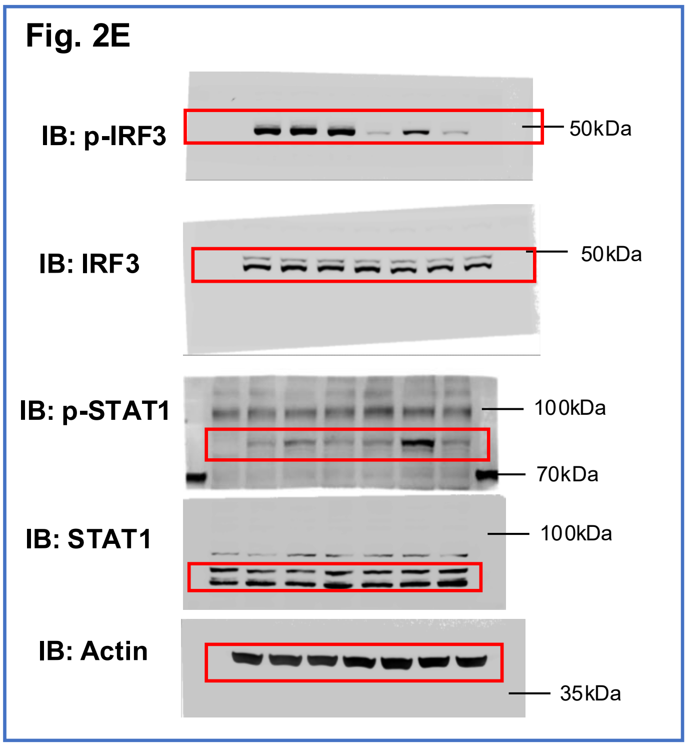
Fig. 2E
IB: p-IRF3
50kDa
IB: IRF3
50kDa
IB: p-STAT1
100kDa
70kDa
IB: STAT1
100kDa
IB: Actin
35kDa

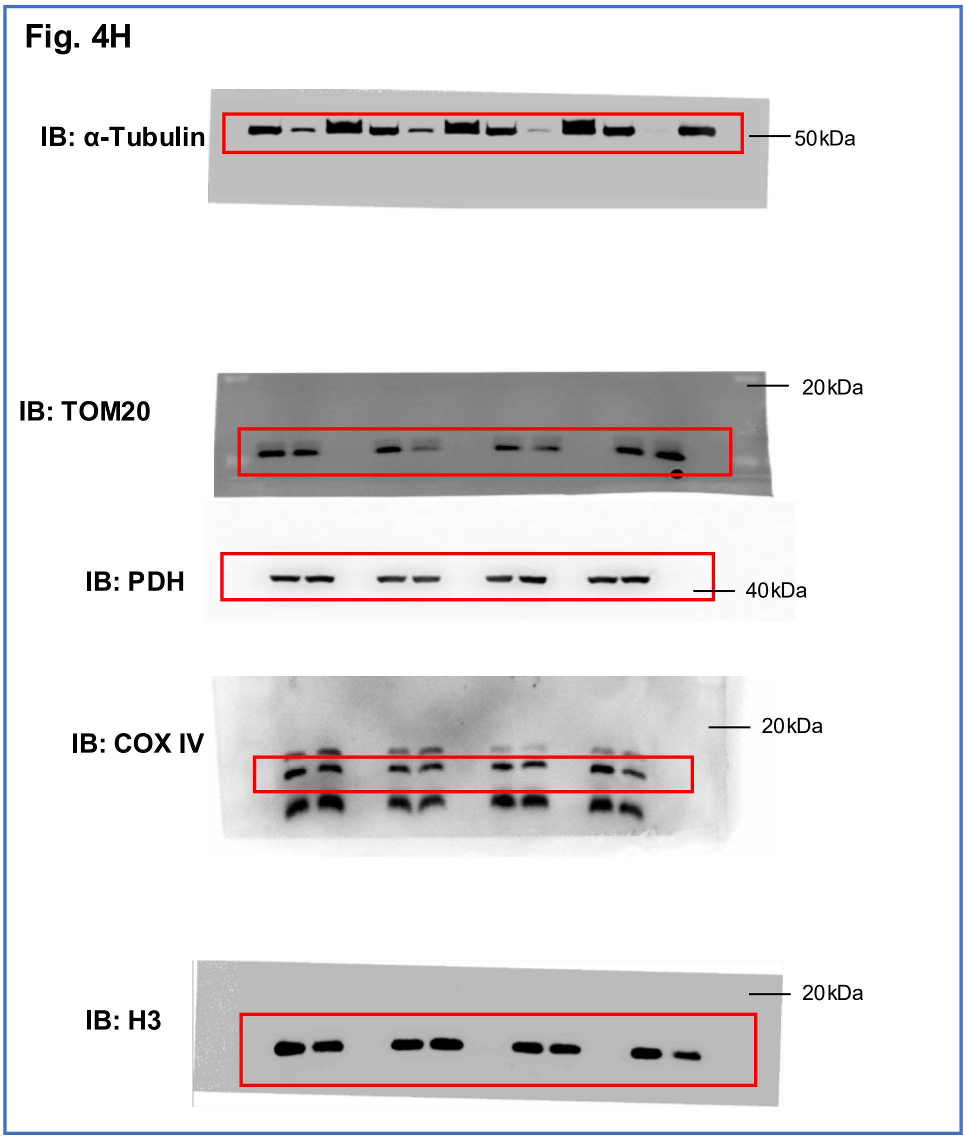
Fig. 4H
IB: α-Tubulin
50kDa
IB: TOM20
20kDa
IB: PDH
40kDa
IB: COX IV
20kDa
IB: H3
20kDa

Fig. 5A

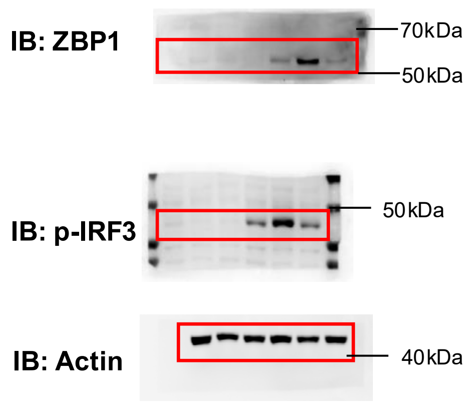

Fig. 5C

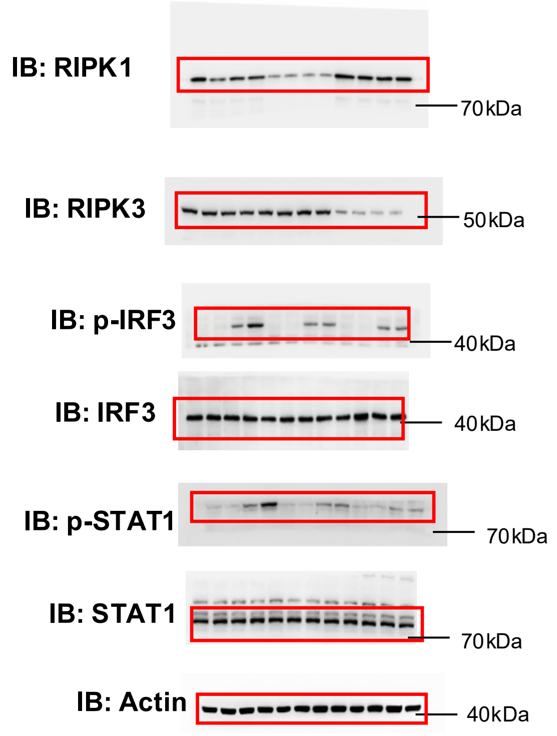

Fig. 5D（Top）

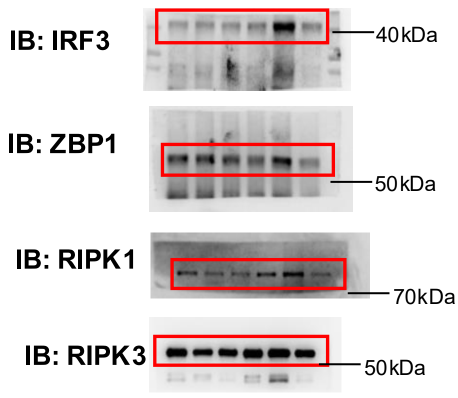

Fig. 5D（Middle）

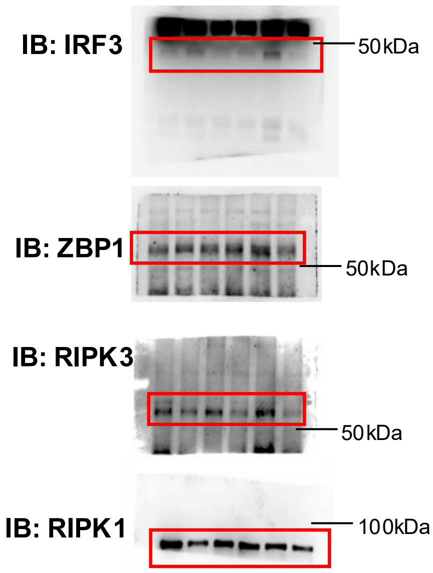

Fig. 5D（Bottom）

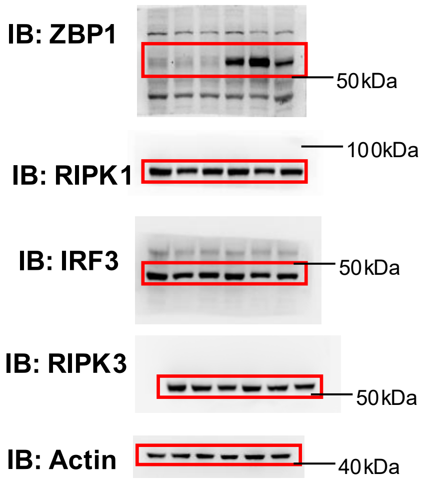

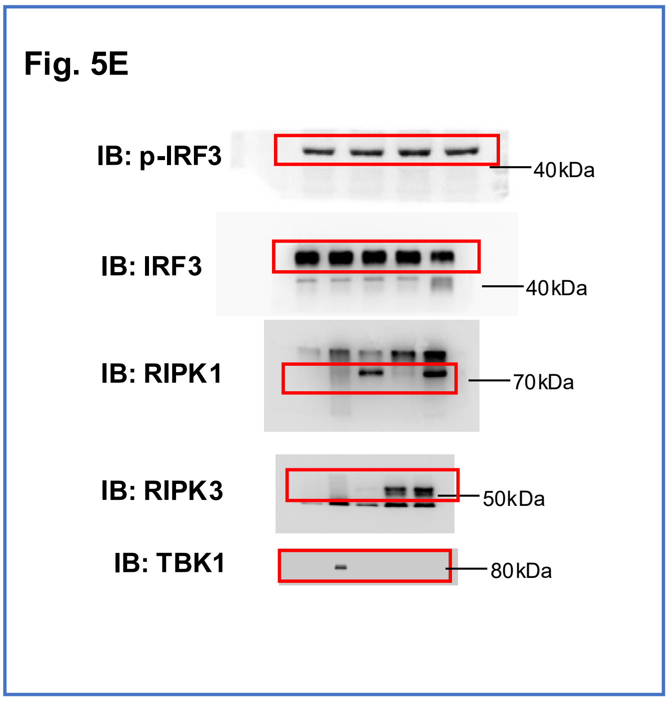
Fig. 5E
IB: p-IRF3
40kDa
IB: IRF3
40kDa
IB: RIPK1
70kDa
IB: RIPK3
50kDa
IB: TBK1
80kDa

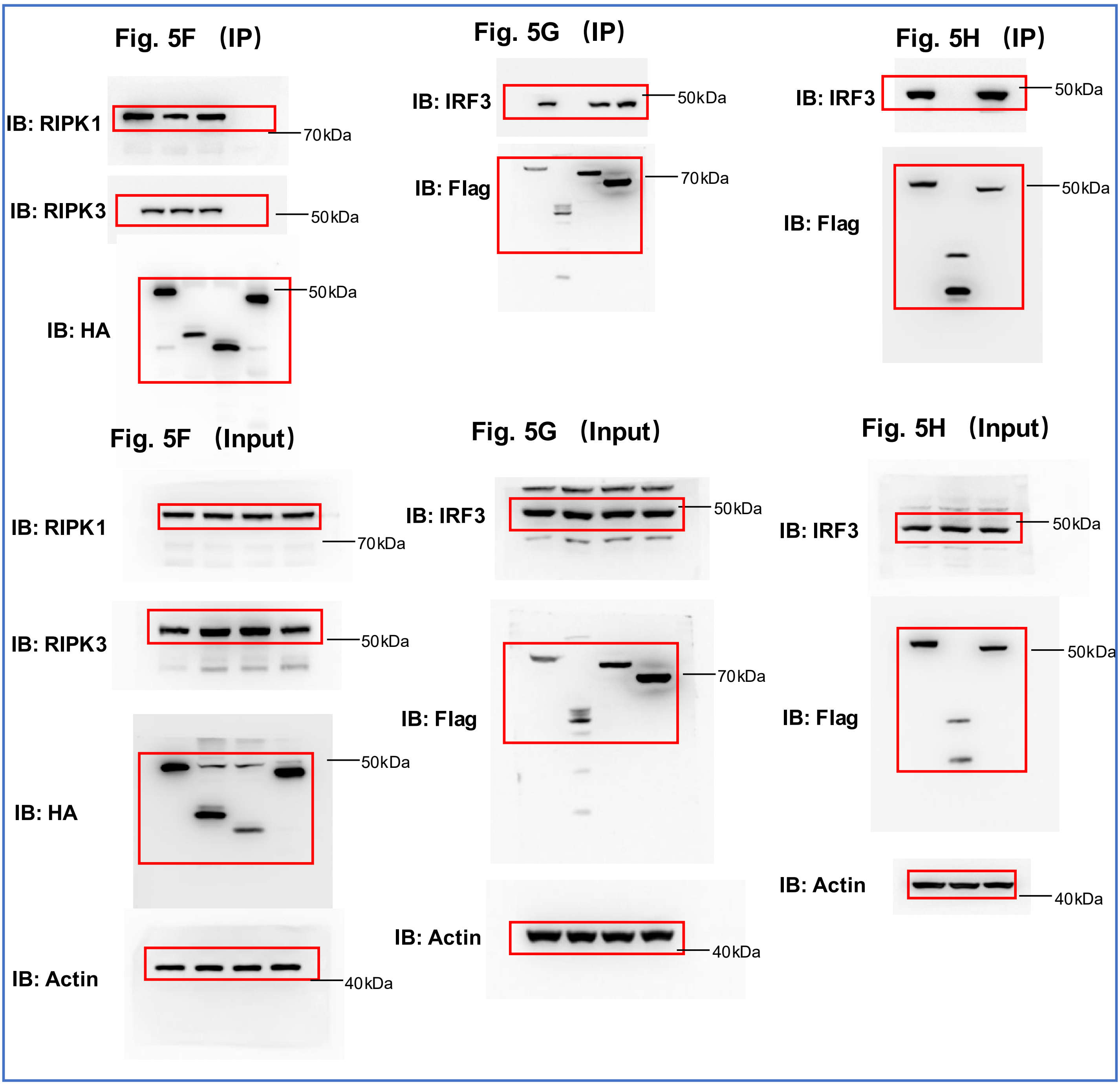
Fig. 5F (IP)
IB: RIPK1
70kDa
IB: RIPK3
50kDa
IB: HA
50kDa
Fig. 5G (IP)
IB: IRF3
50kDa
IB: Flag
70kDa
Fig. 5H (IP)
IB: IRF3
50kDa
IB: Flag
50kDa
Fig. 5F (Input)
IB: RIPK1
70kDa
IB: RIPK3
50kDa
IB: HA
50kDa
IB: Actin
40kDa
Fig. 5G (Input)
IB: IRF3
50kDa
IB: Flag
70kDa
IB: Actin
40kDa
Fig. 5H (Input)
IB: IRF3
50kDa
IB: Flag
50kDa
IB: Actin
40kDa

Fig. 7A

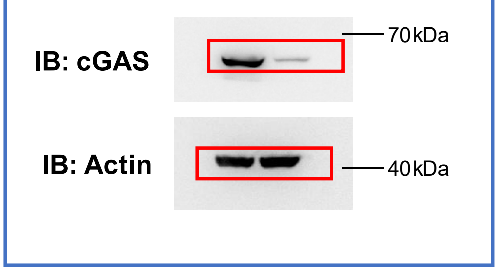

Fig. 7F

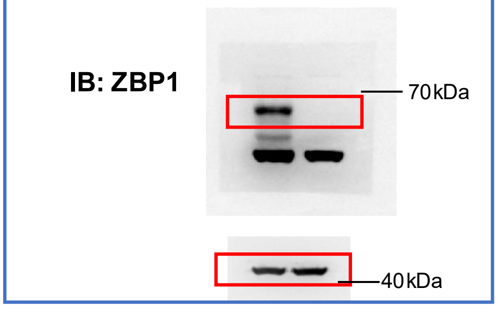

Fig. 7K

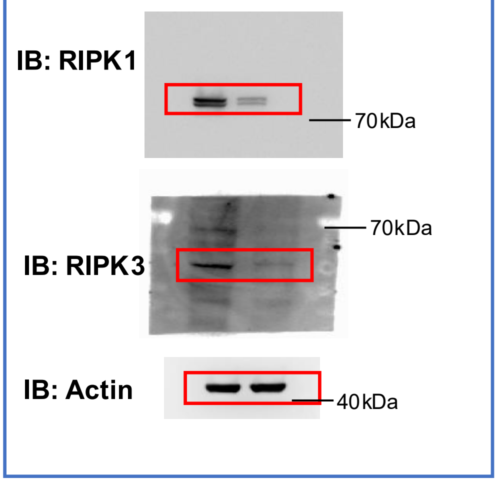

Fig. 7P

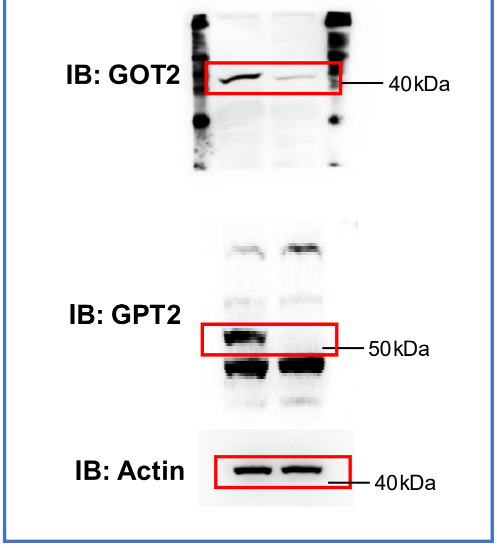

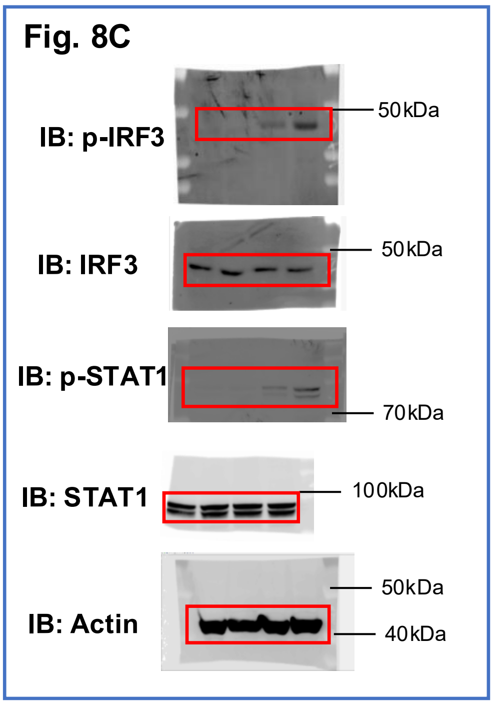

Fig. 8C
IB: p-IRF3
50kDa
IB: IRF3
50kDa
IB: p-STAT1
70kDa
IB: STAT1
100kDa
IB: Actin
50kDa
40kDa

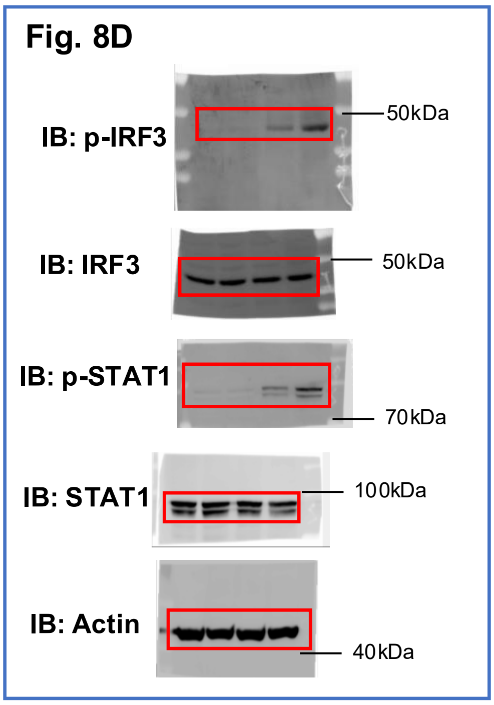

Fig. 8D
IB: p-IRF3
50kDa
IB: IRF3
50kDa
IB: p-STAT1
70kDa
IB: STAT1
100kDa
IB: Actin
40kDa

Sup Fig. 1H

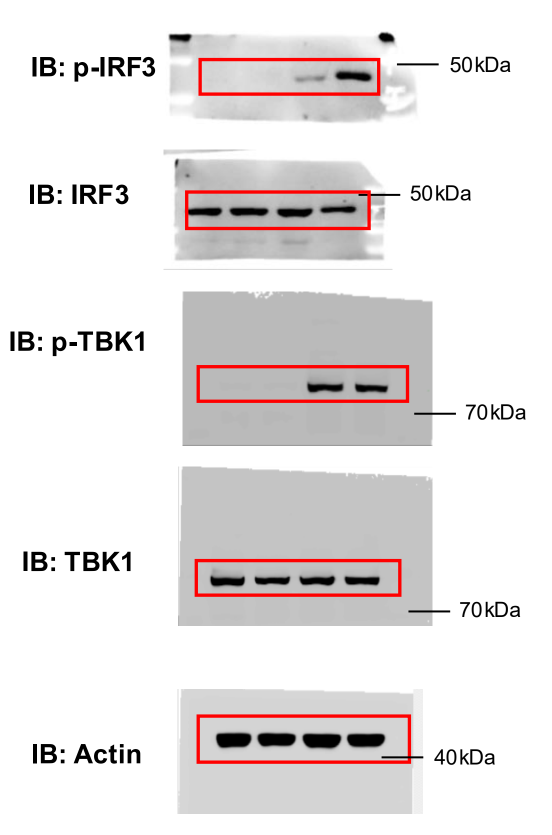

Sup Fig. 1I

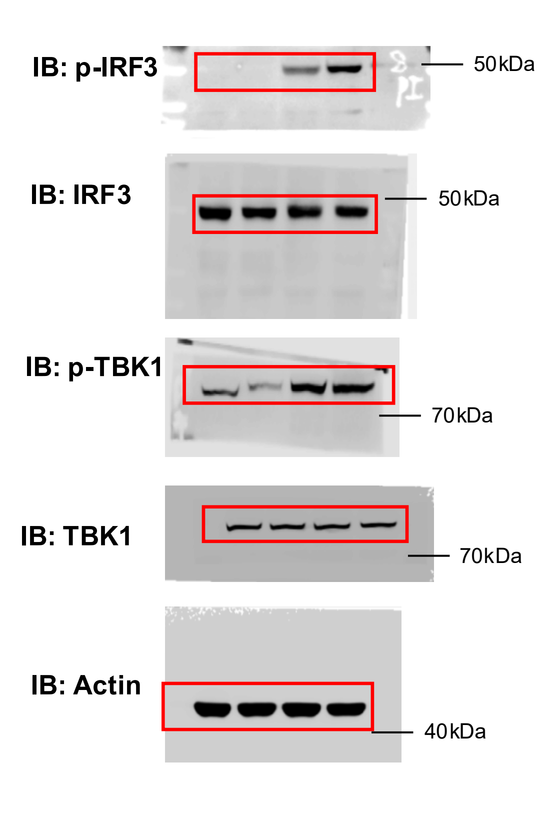

Sup Fig. 1J

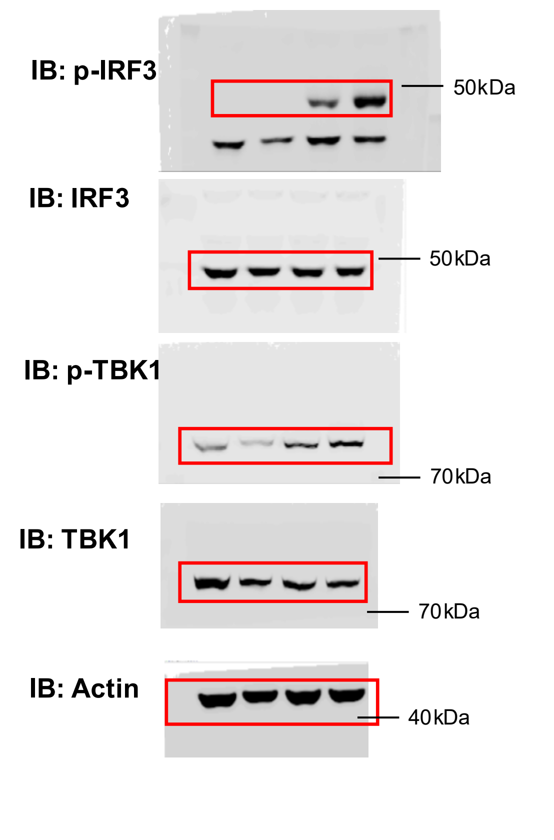

Sup Fig. 1K

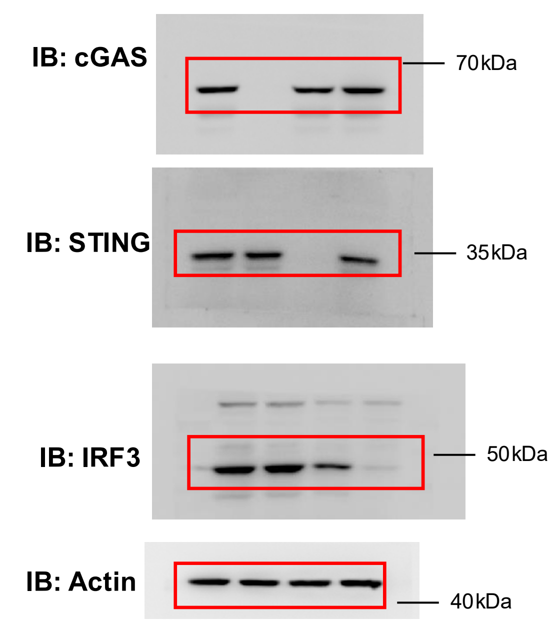

Sup Fig. 2G

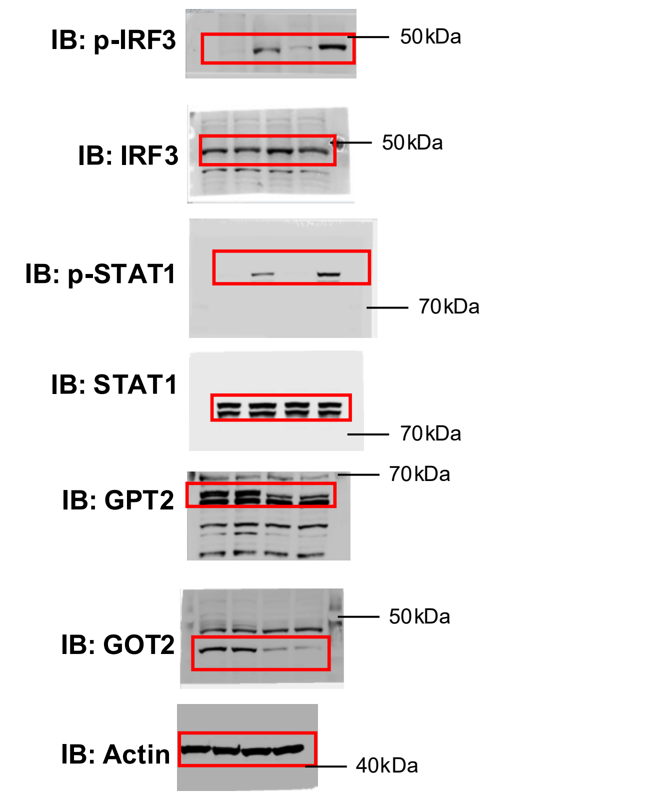

Sup Fig. 2I

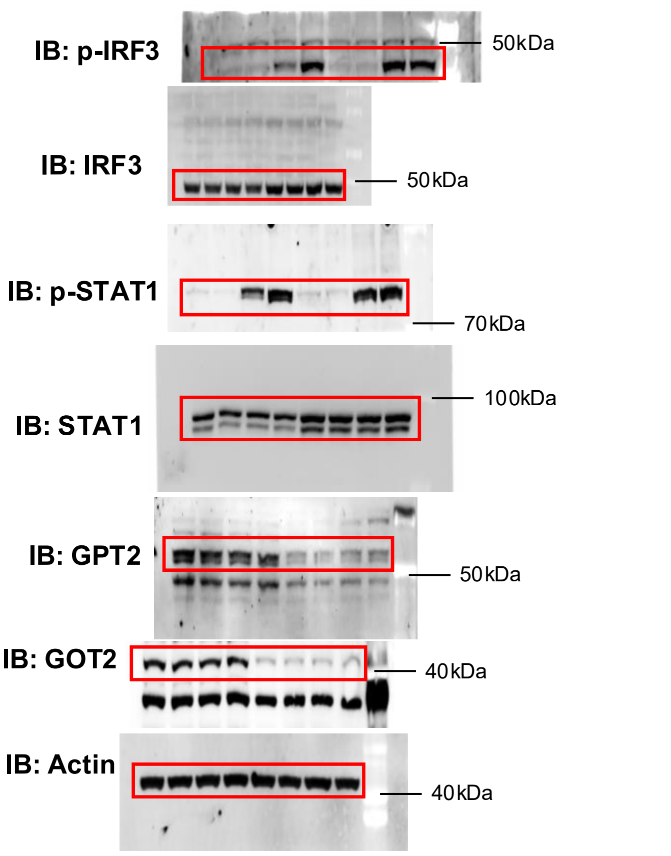

Sup Fig. 3I

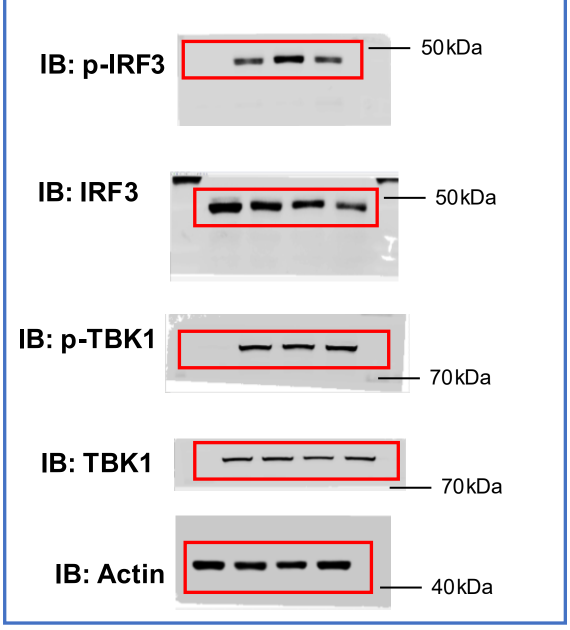

Sup Fig. 3J

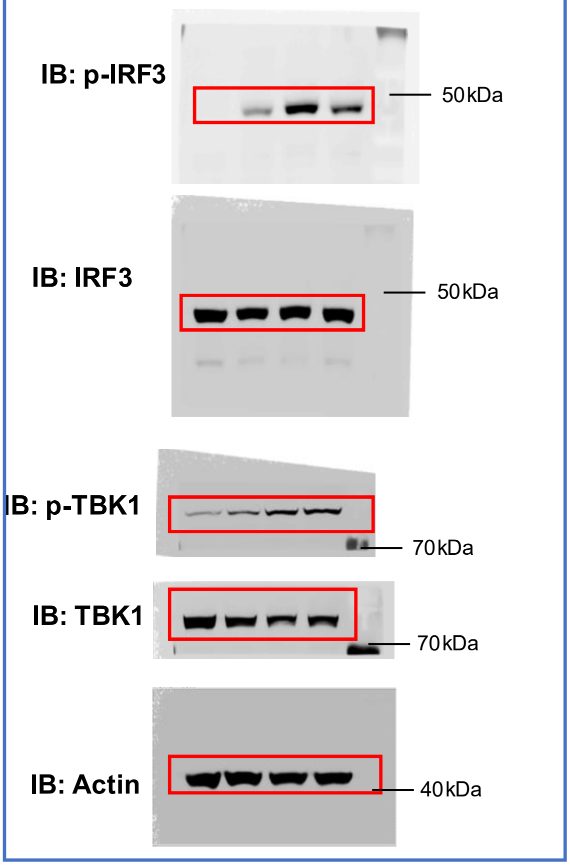

Sup Fig. 3K

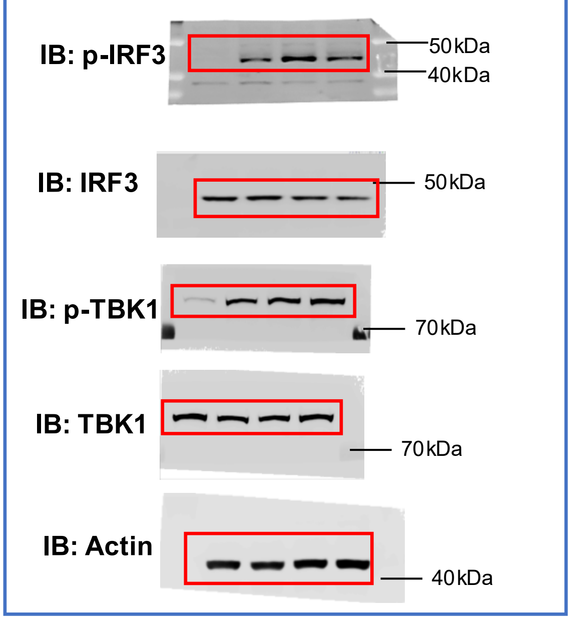

Sup Fig. 3L

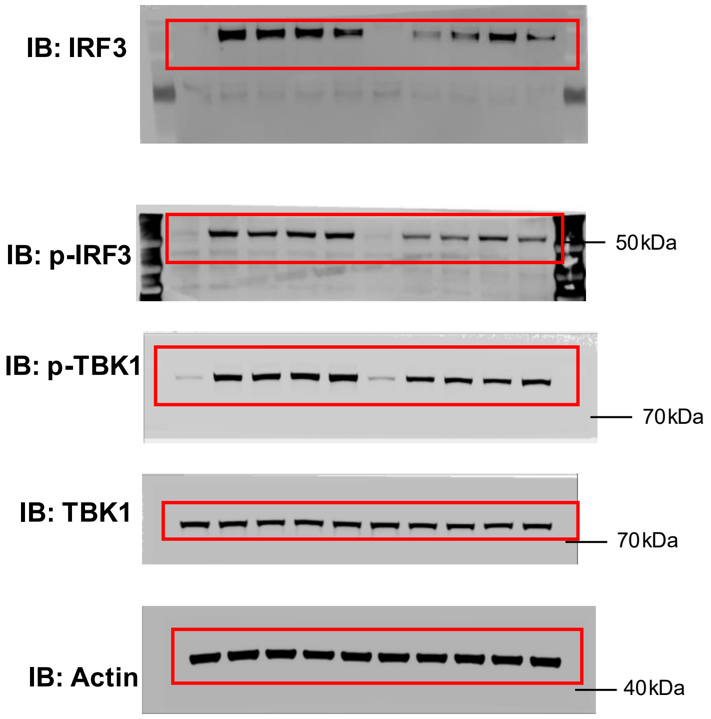

Sup Fig. 3M

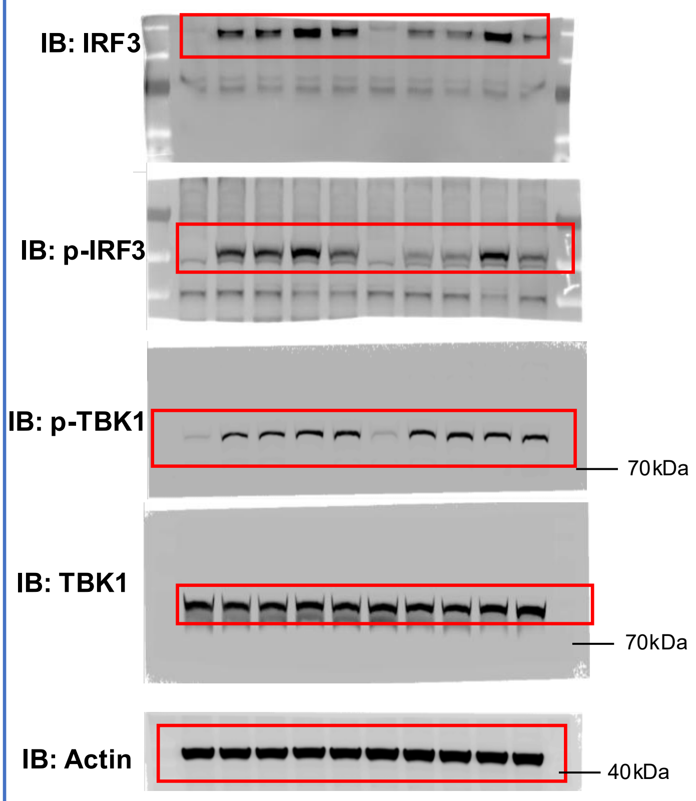

Sup Fig. 6A

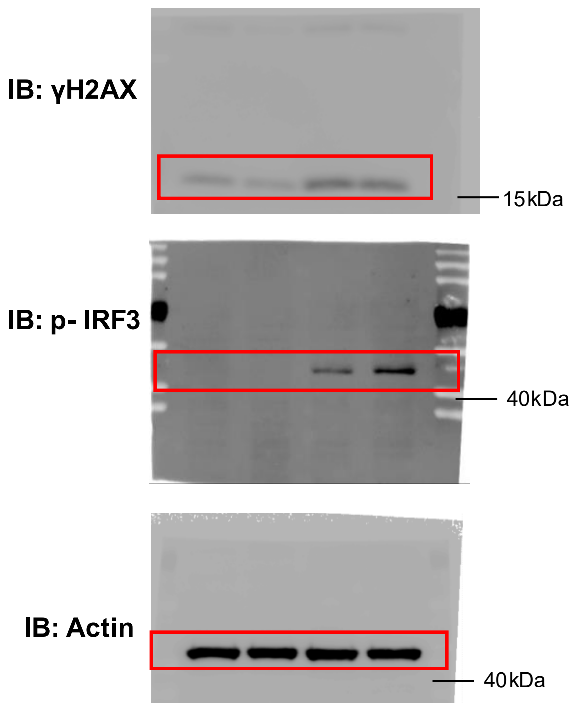

Sup Fig. 6B

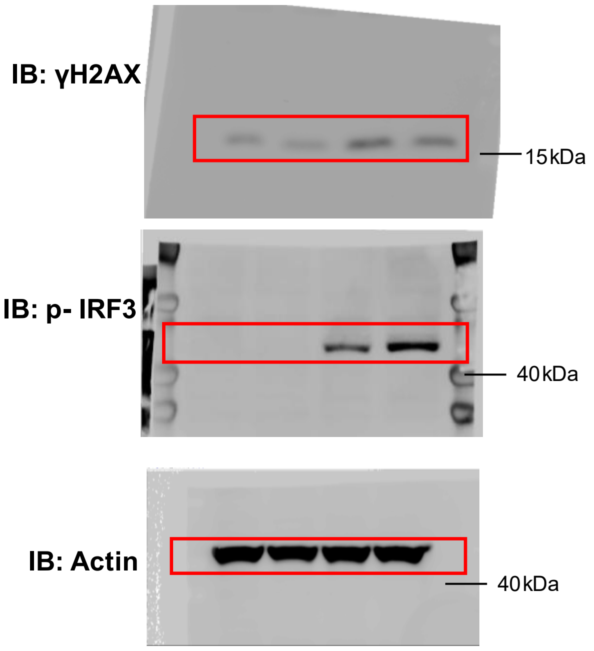

Sup Fig. 6C

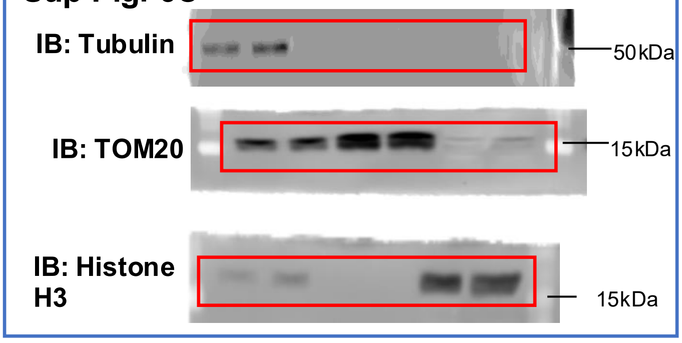

Sup Fig. 6G

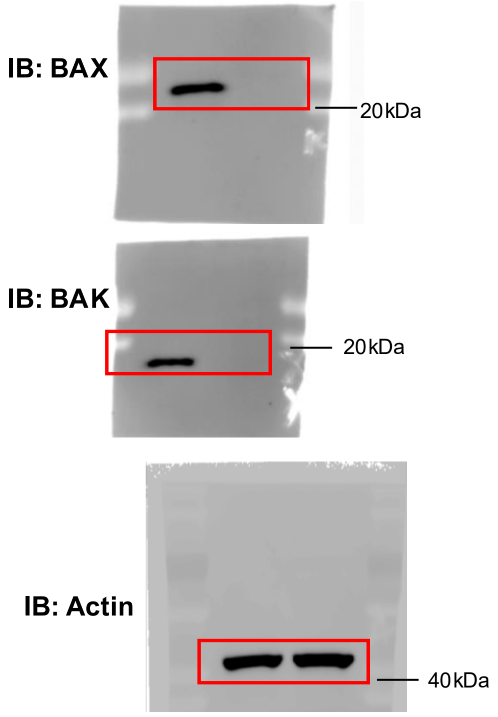

Sup Fig. 7B

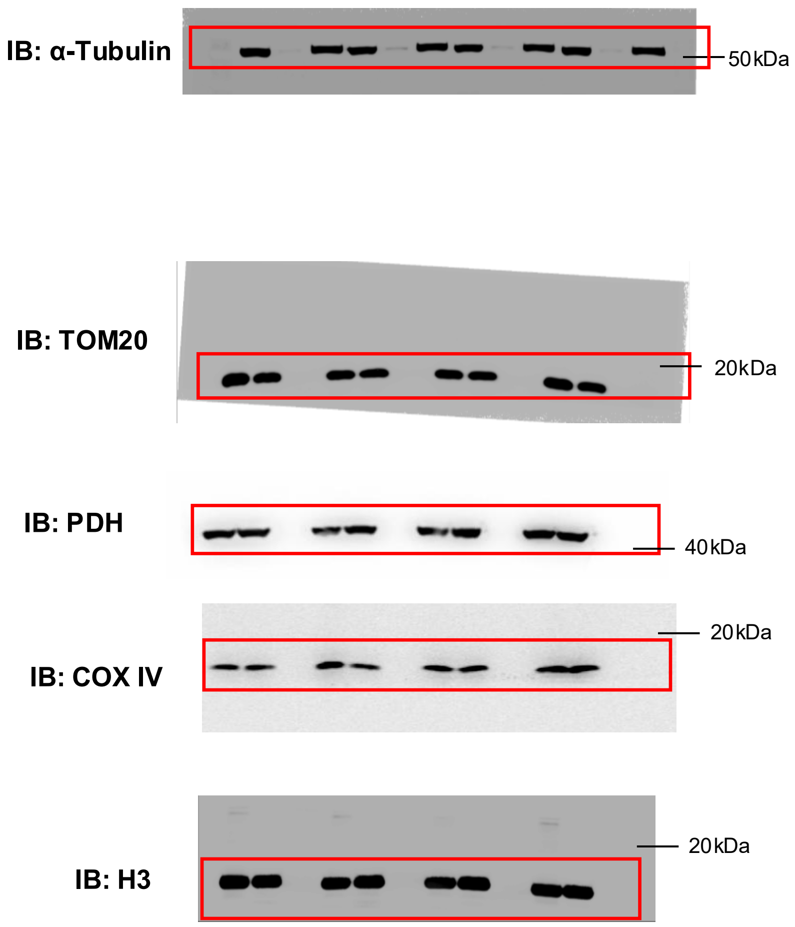

IB: α-Tubulin
50kDa
IB: TOM20
20kDa
IB: PDH
40kDa
IB: COX IV
20kDa
IB: H3
20kDa

Sup Fig. 8D

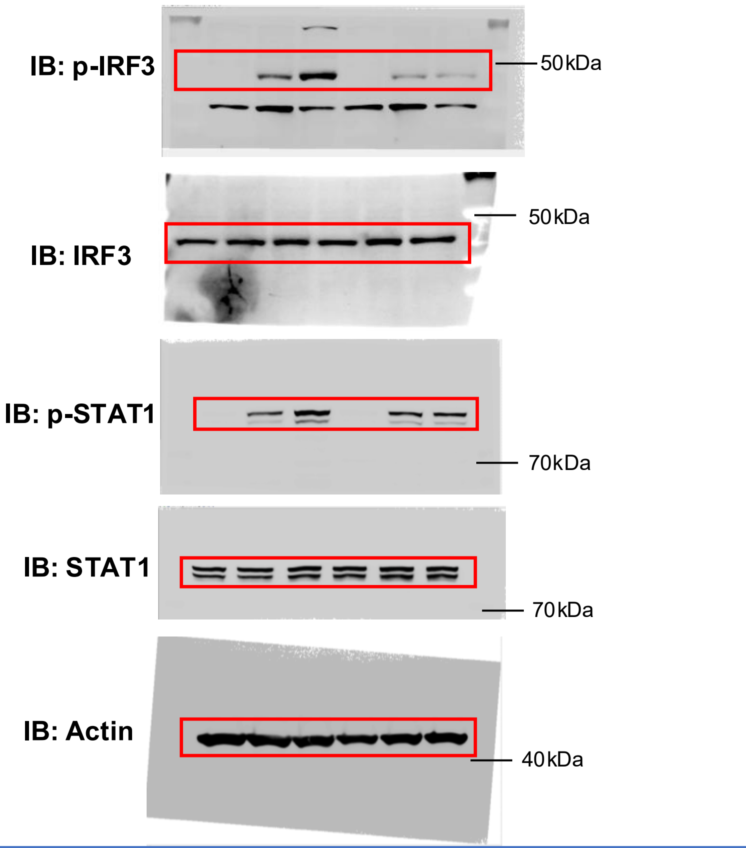

Sup Fig. 8E

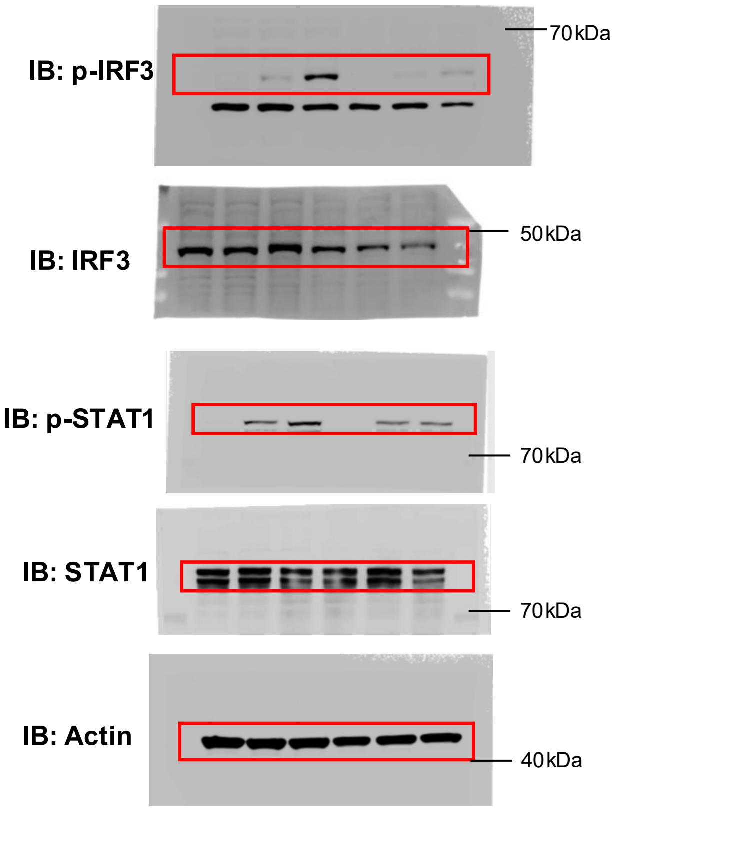

Sup Fig. 9B

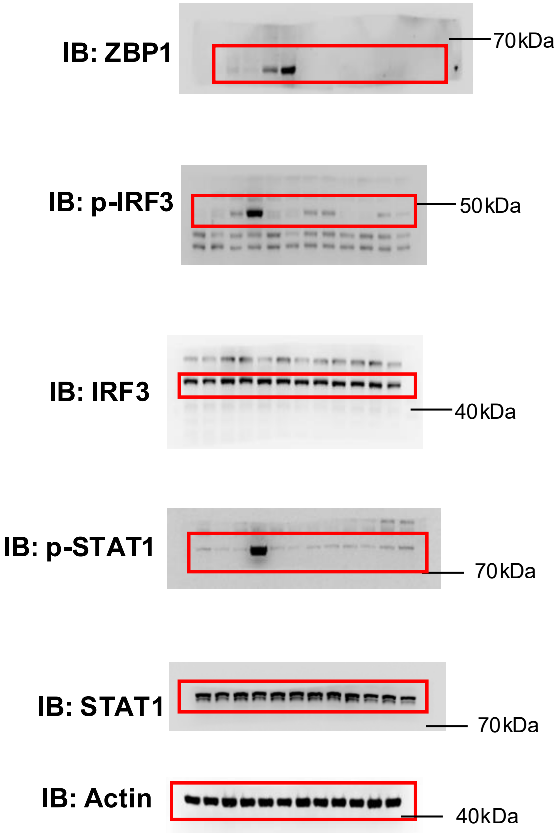

Fig. 9D（IP）

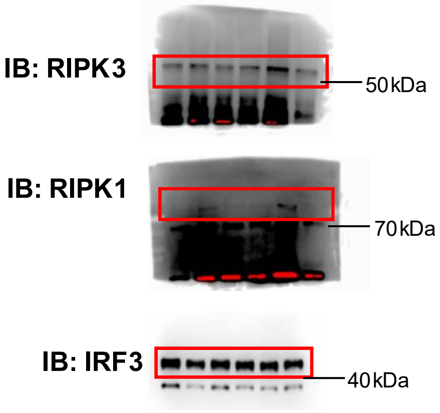

Fig. 9D（Input）

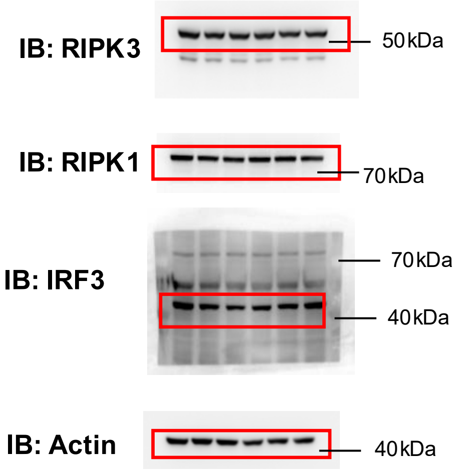

Sup Fig. 9F

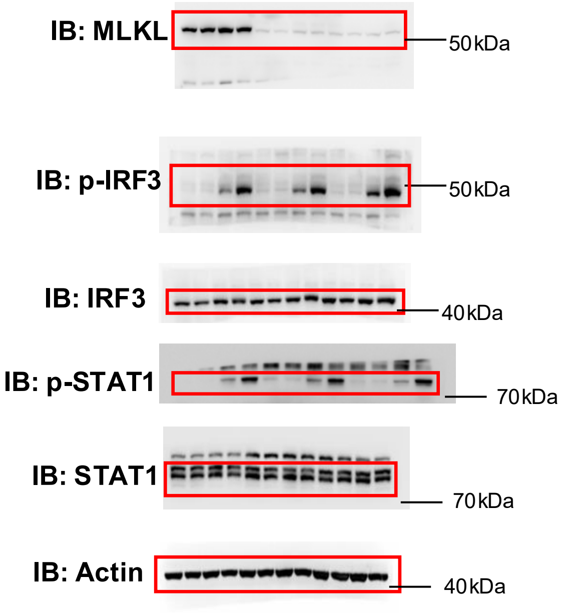

Supplement: Unedited blot and gel images [file jci-136-199716-s021.pdf]
